# Design and Synthesis of New GS-6207 Subtypes for Targeting HIV-1 Capsid Protein

**DOI:** 10.3390/ijms25073734

**Published:** 2024-03-27

**Authors:** Thamina Akther, William M. McFadden, Huanchun Zhang, Karen A. Kirby, Stefan G. Sarafianos, Zhengqiang Wang

**Affiliations:** 1 Center for Drug Design, College of Pharmacy, University of Minnesota, Minneapolis, MN 55455, USA; akthe002@umn.edu; 2 Center for ViroScience and Cure, Laboratory of Biochemical Pharmacology, Department of Pediatrics, Emory University School of Medicine, Atlanta, GA 30322, USA; will.mcfadden@emory.edu (W.M.M.); huanchun.zhang@emory.edu (H.Z.);

**Keywords:** HIV-1 capsid, GS-6207, PF74, molecular modeling, synthesis

## Abstract

HIV-1 capsid protein (CA) is the molecular target of the recently FDA-approved long acting injectable (LAI) drug lenacapavir (GS-6207). The quick emergence of CA mutations resistant to GS-6207 necessitates the design and synthesis of novel sub-chemotypes. We have conducted the structure-based design of two new sub-chemotypes combining the scaffold of GS-6207 and the N-terminal cap of PF74 analogs, the other important CA-targeting chemotype. The design was validated via induced-fit molecular docking. More importantly, we have worked out a general synthetic route to allow the modular synthesis of novel GS-6207 subtypes. Significantly, the desired stereochemistry of the skeleton C^2^ was confirmed via an X-ray crystal structure of the key synthetic intermediate **22a**. Although the newly synthesized analogs did not show significant potency, our efforts herein will facilitate the future design and synthesis of novel subtypes with improved potency.

## 1. Introduction

**Figure 1:**
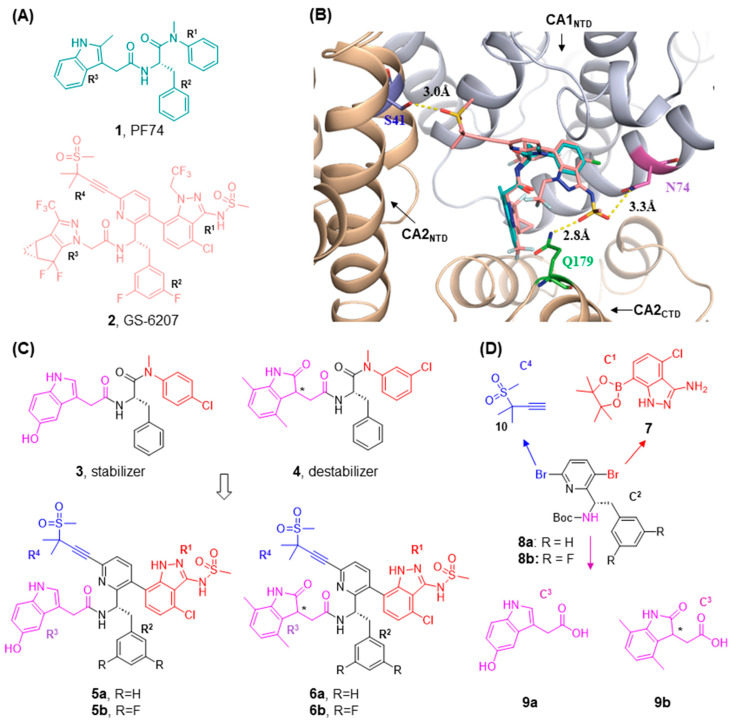
Design of novel HIV CA-targeting chemotypes. (**A**) Chemical structures of **1**, PF74, and **2**, GS-6207. (**B**) Docking of the two best-known CA inhibitors **1**, PF74 (cyan), and **2**, GS-6207 (crimson), into the presumed binding pocket (PDB: 4XFZ). Overlay shows that both chemical scaffolds bind similarly. (**C**) Chemical profiling of **1**, PF74, identified analog **3** as a potent stabilizer and analog **4** as a unique destabilizer. Molecular hybridization led to the design of hybrids **5** and **6**. (**D**) Modular synthetic approach entailing the key skeleton C^2^ (**8a**,**b**) and sequential reactions with C^1^ (**7**, Suzuki), C^4^ (**10**, Sonogashira), and C^3^ (**9a**,**b**, amide coupling). Asterisks (*) indicate stereocenters with undefined configurations.

The multifunctional capsid protein (CA) of HIV-1 has become an increasingly attractive target for developing novel antiviral drugs [1,2,3,4,5]. Although multiple small-molecule binding sites at both the N-terminal domain of CA (CA_NTD_) and the C-terminal domain (CA_CTD_) are known [6], the pocket used by host factors NUP153 [7,8], CPSF6 [9,10,11], and Sec24C [2,12] to facilitate nuclear entry and integration is particularly druggable. Notably, the two most prominent CA inhibitor types, represented by PF74 [11,13,14,15] (**1**) and GS-6207 [16,17] (**2**), both bind to this pocket to compete against NUP153, CPSF6 [10,11,18], and Sec24C [2,12] in addition to impacting the capsid stability. As a result, these CA binders confer potent antiviral phenotypes with dose-dependent multimodal mechanisms of action [15,16]. PF74 is a phenylalanine-derived peptidomimetic with an aniline cap at the C-terminus and an indole-3-acetic acid cap at the N-terminus (Figure 1A). Despite its potent and mechanistically interesting antiviral phenotypes, PF74 is not a viable antiviral lead due to the prohibitively low metabolic stability [19]. We have chemically profiled PF74 extensively with the synthesis of a large number of analogs [20], and along with others, have explored different PF74 subtypes for enhanced metabolic stability [6,19,21,22,23,24,25,26,27,28,29,30,31,32]. GS-6207 (lenacapavir), on the other hand, is extraordinarily stable toward oxidative metabolism, presumably owing to its high fluorine content (Figure 1A). The unusually high metabolic stability, along with its exceptional antiviral potency, renders GS-6207 a landmark antiviral drug recently approved as an LAI [33]. However, CA mutations highly resistant to GS-6207 [34,35,36], particularly M66I, Q67H, K70R, and N74D, have been selected in vitro and in patients, suggesting that novel subtypes need to be designed and synthesized to curb resistance. Such endeavors are expected to be more challenging than those typically encountered in developing new subtypes due to the structural complexity of GS-6207 and its snug-fit binding into the presumed pocket. We report herein our efforts in the design and synthesis of two GS-6207 subtypes featuring the generic backbone of GS-6207 and the indole-acetic acid moiety of PF74 (Figure 1C).

## 2. Results and Discussion

### 2.1. Design of Novel PF74/GS-6207 Molecular Hybrids ***5*** and ***6***

The design of the molecular hybrids is based on the shared binding mode of **1**, PF74, and **2**, GS-6207 (Figure 1B). Although vastly different in molecular complexity and functional group density, the two molecules bind to the same pocket at the interface of two adjacent CA promoters (CA1 and CA2). **2**, GS-6207, is well superimposed with **1**, PF74, through the backbone and the R^2^ and R^3^ moieties, with shared key molecular interactions in the N-terminal domain of CA1 (CA1_NTD_) and the C-terminal domain of the adjacent CA2 (CA2_CTD_) (Figure 1B). The additional interactions conferred by **2**, GS-6207, with CA2_NTD_ via the sulfone group of the R^4^ moiety and CA2_CTD_ via the methanesulfonamide group of the R^1^ moiety likely contribute substantially to its superior potency over **1**, PF74, and will be retained in our design. Interestingly, from our previous chemical profiling, **1**, PF74 analog **3** showed a drastically improved potency (>27-fold) and superb capsid stabilizing effect, as indicated by a large positive shift in the thermal shift assay (TSA) [6]. Another PF74 analog **4**, featuring a distinct indolone R^3^ moiety, displayed a highly unusual destabilizing effect (negative shift in TSA) [6]. An important aim of our redesign is to optimize both the stabilizer lead (**3**) and the destabilizer lead (**4**) through design and synthetic strategies aligned with improving the resistance profile of **2**, GS-6207. Based on these, molecular hybrids **5** and **6** were designed, as shown in Figure 1C. To enhance the synthetic accessibility, the design also features a slightly simplified R^1^ moiety for both **5** and **6** and undefined stereochemistry in the indolone ring of hybrid **6**. Finally, the newly designed hybrids will contain the R^2^ moiety of both **1**, PF74 (R = H), and **2**, GS-6270 (R = F). The main aim of this pilot design and synthesis is to develop a general and amenable synthesis to enable the future redesign and synthesis of structurally more elaborate subtypes of **2**, GS-6207.

### 2.2. Molecular Docking Analysis

**Figure 2:**
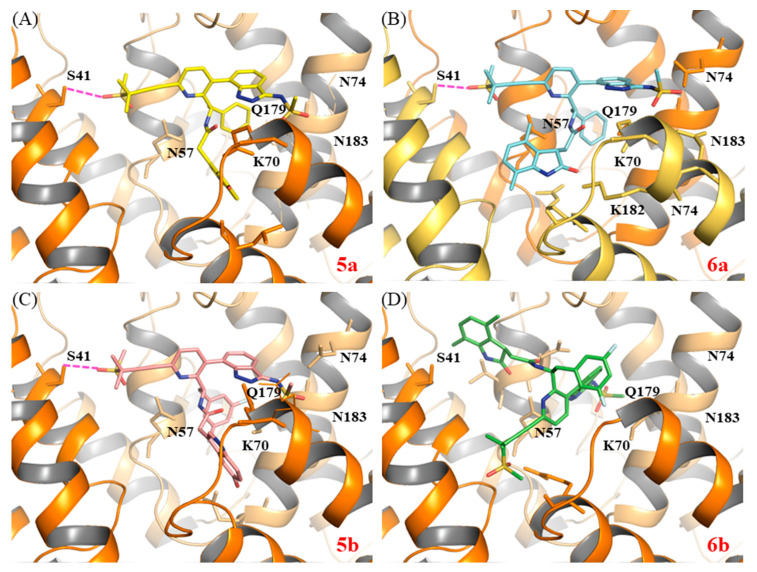
Docking of the four newly designed analogs into PF74-bound HIV-1 CA (PDB ID: 4XFZ) [37]. Control docking was conducted with **2**, GS-6207 (Glide score = −13.41 kcal/mol). Predicted binding modes of (**A**) compound **5a** (Glide score = −12.31 kcal/mol); (**B**) compound **6a** (Glide score = −12.73 kcal/mol); (**C**) compound **5b** (Glide score = −14.07 kcal/mol); and (**D**) compound **6b** (Glide score = −10.32 kcal/mol). Hydrogen bonding interactions are depicted as pink dashed lines. CA_NTD_ is shown in orange cartoon and adjacent CA_CTD_ in gold cartoon, with key residues around binding site shown as orange sticks. The nitrogen, oxygen, fluorine and chlorine atoms are colored blue, red, light blue, and green, respectively.

To computationally validate the design, we performed induced-fit molecular docking for all four analogs, **5a**, **5b**, **6a,** and **6b,** using the co-crystal structure of PF74-bound HIV-1 CA (PDB code: 4XFZ) [37]. A control docking was conducted with **2**, GS-6207 (docked pose shown in Figure 1B). Overall, these newly designed analogs all docked well into the **2**, GS-6207, binding site (Figure 2), with docking scores comparable to that of **2**, GS-6207. Major molecular interactions observed include (1) H-bonds between the sulfonamide oxygen and the side chain of Ser41 (CA2_NTD_) and Asn57 (CA1_NTD_); (2) H-bonds between the side chain of Asn57 (CA1_NTD_) and both the ring-nitrogen atom of the core and the amide N−H of the inhibitor; (3) H-bonds between the methanesulfonamide and the side chains of Asn183 (CA2_CTD_), Gln179 (CA2_CTD_), Lys70 (CA1_NTD_), and Asn74 (CA1_NTD_); and (4) H-bond between the side chain of Lys70 (CA1_NTD_) and the carbonyl oxygen of the inhibitor. For the newly incorporated indole or indolone moiety (R^3^), the free NH forms an H-bond with the side chain Arg173 (CA2_CTD_, **6a**), Gln63 (CA1_NTD_, **5a**), and Thr54 (CA1_NTD_, **6b**). In addition, the OH on the indole ring is H-bonded with the side chain Gln179 (CA2_CTD_, **6a**), Leu172 (CA2_CTD_, **5a**), and Gln63 (CA1_NTD_, **5b**). These docking results indicate that most of the molecular interactions conferring high potency of **2**, GS-6207, are retained in the newly designed hybrids.

### 2.3. Synthesis of Hybrids ***5*** and ***6***

The general synthetic approach is depicted in Figure 1D. The synthesis is highly modular based on four synthetic components (C^1^–C^4^) for installing R^1^–R^4^. C^2^ is the core with the proper functional group handles to allow the installation of R^1^, R^4^, and R^3^ via sequential reactions with C^1^ (Suzuki), C^4^ (Sonogashira), and C^3^ (amide coupling), respectively. This modular synthesis will support future synthetic needs of structural diversification in all four structure–activity relationship (SAR) regions (R^1^–R^4^), particularly the regions of R^1^, R^4^, and R^3^.

**Scheme 1:**
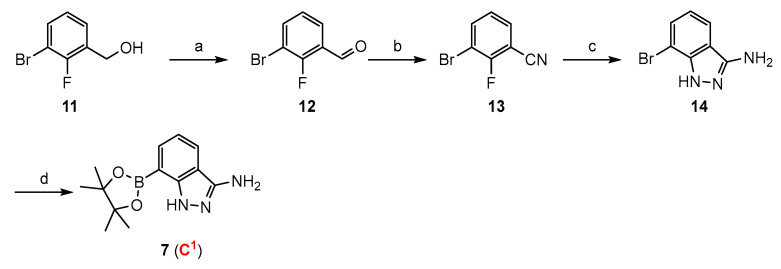
Synthesis of component C^1^ (compound **7**). Reagents and conditions: (a) MnO_2_, DCM, rt, 8 h, 87%; (b) H_2_NOH.H_2_O, Ac_2_O, AcOH, 75 °C, 6 h, 71%; (c) H_2_NNH_2_.H_2_O, EtOH, 90 °C, 4 h, 60%; (d) B_2_Pin_2_, Pd(PPh_3_)_2_Cl_2_, KOAc, 1,4-dioxane 110 °C, 18 h, 50%.

The synthetic route for the preparation of component C^1^ is shown in Scheme 1. MnO_2_ oxidation [38] of commercially available benzyl alcohol **11** afforded benzaldehyde intermediate **12**. The subsequent conversion to nitrile **13** was effected via the standard method of oxime formation and dehydration [39]. The nucleophilic aromatic substitution reaction [40] at the F site by hydrazine followed by the cyclization reaction produced aminoindazole **14**, which was subjected to the palladium-catalyzed borylation [41] with bis(catecholato)diboron to yield the representative C^1^, compound **7**.

**Scheme 2:**
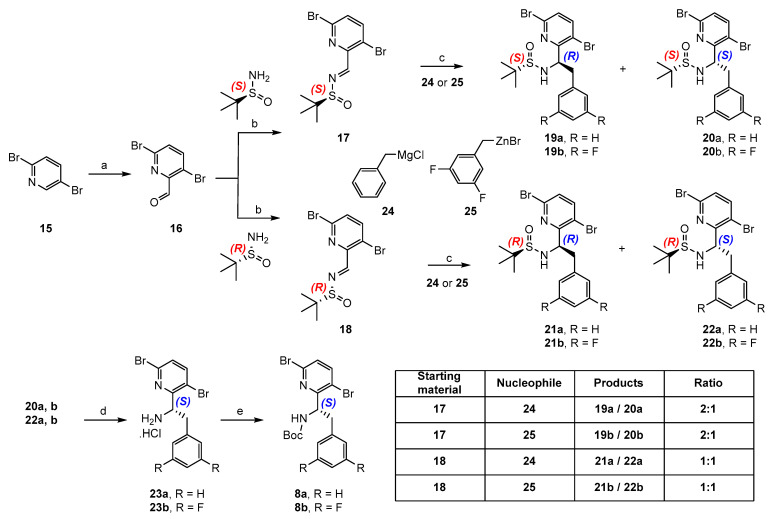
Synthesis of C^2^ (**8a** and **8b**). Reagents and conditions: (a) TMPMgCl.LiCI, THF, DMF, −20 °C, 8 h, 56%; (b) Cs_2_CO_3_, NMP, rt, 4 h, 60%; (c) dry THF, −78 °C, 2 h, 65%; (d) 4N HCl/dioxane, MeOH, rt, 3 h, 76%; (e) (Boc)_2_O, NEt_3_, DCM, rt, 2 h, 80%.

The synthesis of the core component C^2^ began with the commercially available 2,5-dibromopyridine **15** (Scheme 2). Deprotonative formylation [42] of **15** with TMPMgCl.LiCl in dry THF followed by the addition of DMF afforded aldehyde intermediate **16**, which set the stage for the key asymmetric induction via a chiral auxiliary. The chiral auxiliary was introduced via condensation of aldehyde **16** with chiral non-racemic (*S*)- and (*R*)-*tert*-butanesulfinamides to produce (*S*)-*tert*-butanesulfinyl imine **17** and (*R*)-*tert*-butanesulfinyl imine **18**, respectively (Scheme 2) [43].

**Figure 3:**
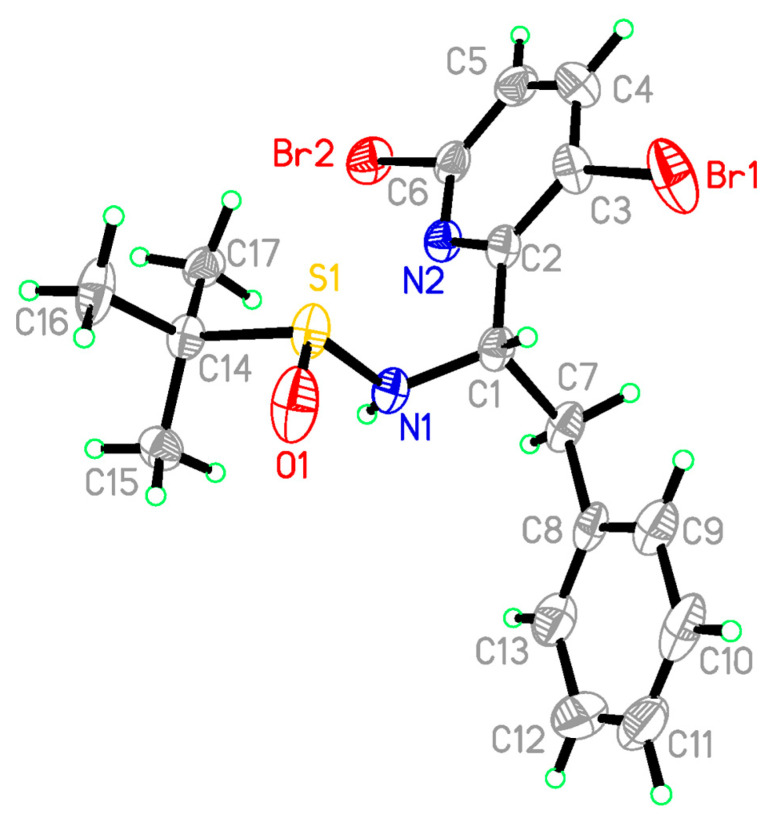
X-ray crystal structure of **22a**.

Both auxiliaries were used to determine the preferred auxiliary/nucleophile combination for producing the desired stereochemical outcome (S) for C^2^ (**8a** and **8b**). Specifically, in four different reactions shown in the table (Scheme 2), commercially available benzyl magnesium chloride **24** or (3,5-difluorobenzyl)zinc bromide **25** was used as the nucleophile to react with both sulfinylimines **17** and **18** to afford four pairs of diastereomers (**19a**/**20a**, **19b**/**20b**, **21a**/**22a**, and **21b**/**22b**) in ratios of 1˗2 favoring the undesired diastereomers. From these experiments, it was clear that (*R*)-*tert*-butanesulfinyl imine **18** is preferred over the (*S*)-enantiomer **17** for inducing the desired (*S*) stereochemistry in C^2^ (**8a** and **8b**). The structure and absolute stereochemistry of intermediate **22a** were confirmed by single crystal X-ray diffraction analysis (Figure 3). The crystal selected for the study has chirality at C1 *S*. The chains of hydrogen bonds are parallel to the a-axis through the …O-S-N-H… fragment (Figure 3). Deprotection of the sulfinamide under HCl yielded intermediates **23a**,**b**, which were Boc-protected to afford compounds **8a**,**b** as the core C^2^.

**Scheme 3:**
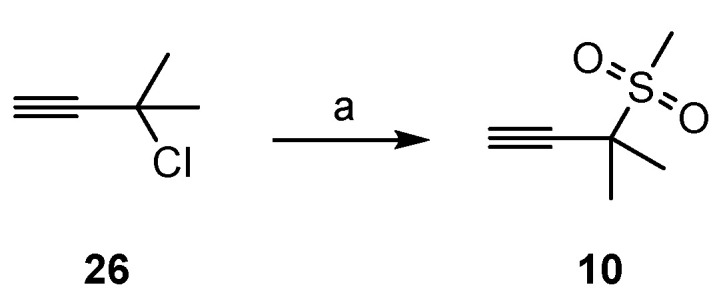
Synthesis of C^4^ (compound **10**). Reagents and conditions: (a) MeSO_2_Na, Cu(I)Cl, DMF, 60 °C, 18 h, 41%.

For this pilot design and synthesis, we used commercially available acids **9a**,**b** as component C^3^. The component C^4^ (compound **10**) was prepared in a single step as described in Scheme 3. *S*-alkylation of sodium methanesulfinate [44] (MeSO_2_Na) with commercially available chloride **26** yielded compound **10** (Scheme 3).

**Scheme 4:**
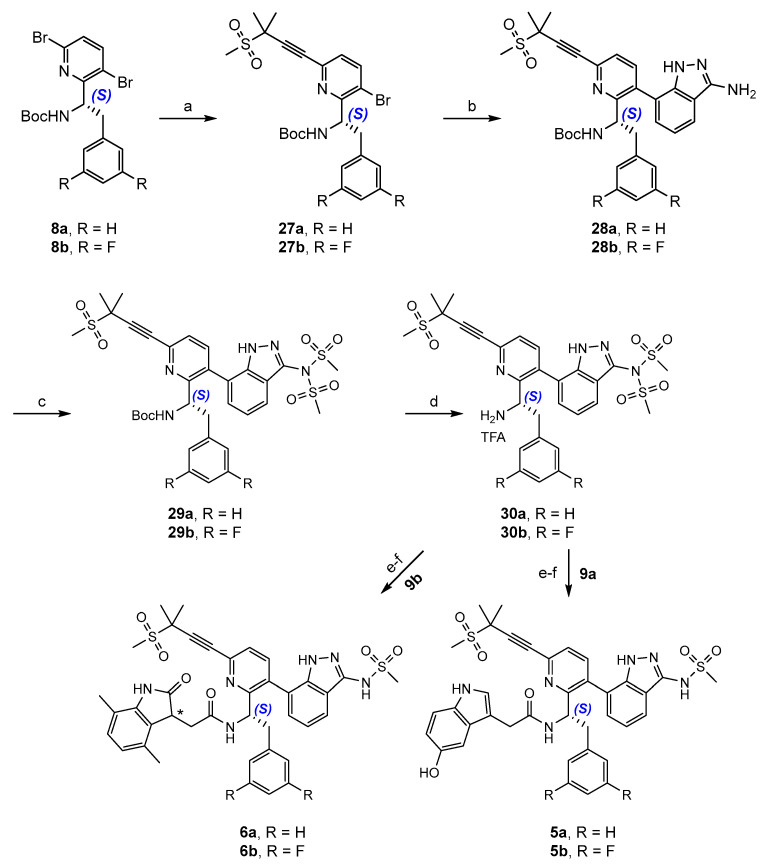
The modular synthesis of newly designed hybrids **5a**,**b** and **6a**,**b**. Reagents and conditions: (a) **10**, Pd(PPh_3_)_2_Cl_2_, CuI, Et_3_N, Dry THF, rt, 6 h, 65%; (b) **7**, Pd(dppf)_2_Cl_2_, K_2_CO_3_, Dioxane/H_2_O, 110 °C, overnight, 61%; (c) Methanesulfonyl chloride, TEA, DCM, rt, 12 h, 73%; (d) TFA, DCM, rt, 12 h; (e) HATU. i-Pr_2_NEt, DMF, rt, 1 h; (f) 2N NaOH, MeOH, rt, 1 h, 41%. Asterisks (*) indicate stereocenters with undefined configurations.

With all four components in hand, the overall modular synthesis was carried out based on the core component C^2^ (compounds **8a**,**b**), as depicted in Scheme 4. The synthesis started with the Sonogashira coupling [45] of component C^2^ (compounds **8a**,**b**) with component C^4^ (compound **10**) to produce intermediates **27a**,**b**. The subsequent Suzuki coupling [46] of **27a**,**b** with component C^1^ (compounds **7**) under the catalysis of Pd(dppf)_2_Cl_2_ afforded intermediates **28a**,**b**. Protection of the free NH_2_ group in component C^1^ with mesylchloride gave bismesylated intermediates **29a**,**b**, which upon Boc deprotection under TFA produced advanced intermediates **30a**,**b**. Finally, the installation of component C^3^ was achieved under standard peptide coupling conditions with HATU as the coupling agent [47], followed by the removal of one mesyl group. As such, commercially available acids **9a**,**b** were incorporated into final compounds **5a**,**b** and **6a**,**b**. The final compounds, **6a**,**b**, were produced as a mixture of diastereomers, which were separated by silica gel column chromatography to afford the desired diastereomer. Following through the purification process, the final compounds **5a**,**b** and **6a**,**b** were successfully crystallized from isopropanol with higher purity.

### 2.4. Biological Analysis of Select Compounds

We tested compounds **5a**, **5b**, **6a**, and **6b** (using PF74 as a control) for their effect on the stability of covalently crosslinked HIV capsid (CA) hexamer. Of these compounds, **5b** demonstrated a positive shift in the melting temperature of the CA hexamer, indicating some stabilization (Table 1). The other three compounds did not provide any stabilization of the CA hexamer. We further tested these four compounds in cell-based antiviral assays for the inhibition of HIV virus activity. Significant toxicity was visibly observed during the antiviral testing, suggesting that the compounds are cytotoxic. This made the EC_50_s values difficult to determine reliably (Table 1). Overall, the compounds did not exhibit much biological activity.

## 3. Materials and Methods

### 3.1. Chemistry

All commercial chemicals were used as supplied unless indicated otherwise. Compounds were purified via flash chromatography using a Combiflash RF-200 (Teledyne ISCO, Lincoln, NE, USA) with RediSep columns (Teledyne ISCO, Lincoln, NE, USA) (silica) and indicated mobile phase. ^1^H and ^13^C NMR spectra were recorded on a Varian 600 MHz (Agilent Technologies, Santa Clara, CA, USA) or Bruker 400 spectrometer (Bruker, Billerica, MA, USA). Diastereomeric ratio was determined by ^1^H NMR analysis. Mass data were acquired using an Agilent 6230 TOF LC/MS spectrometer (Agilent Technologies, Santa Clara, CA, USA). Compound purity analysis was performed using Agilent 1260 Infinity HPLC (Agilent Technologies, Santa Clara, CA, USA) with an Eclipse C18 column (3.5 μm, 4.6 × 100 mm). HPLC conditions: flow rate, 1.0 mL/min; solvent A, 0.1% TFA in water; solvent B, 0.1% TFA in acetonitrile; gradient (B, %): 0–3 min (5–100), 3–11 min (100), 11–13 min (100–5). Determined purity was >85% for all final compounds.

#### 3.1.1. Procedure for Synthesis of **12**

To a solution of commercially available (3-bromo-2-fluorophenyl)methanol (**11**, 40 g, 1.0 equiv.) in 92 mL of DCM, was added MnO_2_ (40 g, 1.05 equiv.) slowly under argon. The reaction mixture was stirred for 8 h at room temperature under an argon balloon. Upon completion, as confirmed by TLC, the reaction mixture was filtered through a pad of celite. The reaction mixture was washed by DCM five times. The combined organic layers were further washed with water and brine, dried over Na_2_SO_4_, and concentrated in vacuo to afford crude intermediate **12**, 3-bromo-2-fluorobenzaldehyde as yellow solid (**12**, 4.34 g, 87%), which was directly used for the next step without further purification. Yield: 87%. ^1^H NMR (600 MHz, CDCl_3_) *δ* 7.42 (ddd, *J* = 8.1, 6.4, 1.6 Hz, 2H), 7.19 (d, *J* = 2.3 Hz, 1H), 6.97 (d, 1H). ^13^C NMR (100 MHz, CDCl_3_) *δ* 158.0, 133.0, 129.5, 129.4, 128.2, 128.1, 125.2, 125.2, 77.2, 59.3, 59.3. HRMS (ESI+) *m/z* calcd for C_7_H_5_BrFO [M+H]^+^ 202.9508, found: 202.9503.

#### 3.1.2. Procedure for Synthesis of **13**

To a solution of 3-bromo-2-fluorobenzaldehyde, (**12**, 49.25 g, 1.0 equiv.) was added acetic anhydride (52.5 g, 1.2 equiv.) and acetic acid (310.5 g) at room temperature, and the reaction mixture was heated to about 45 °C, and hydroxyl amine hydrochloride (15.75 g) was added into the mixture. The reaction was heated at 75 °C and agitated for about 6 h until the reaction was complete. Upon completion, as confirmed by TLC, the product was isolated from the reaction mixture by adding water at about 45 °C. The mixture was cooled to room temperature, and then the slurry was filtered. The filtered cake was washed with water and brine, dried over Na_2_SO_4_, and concentrated to yield 3-bromo-2-fluorobenzonitrile as yellow solid (**13**, 42.0 g, 71%), which was directly used for the next step without further purification. ^1^H NMR (600 MHz, C*D*Cl_3_) *δ* 8.29 (s, 1H), 7.62 (t, *J* = 6.9 Hz, 1H), 7.55–7.47 (m, 1H). ^13^C NMR (100 MHz, C*D*Cl_3_) *δ* 207.4, 158.3, 143.6, 143.6, 134.7, 126.0, 126.0, 125.7, 125.3, 125.3, 121.6, 121.5, 109.9, 109.7, 77.2. HRMS (ESI+) *m/z* calcd for C_7_H_3_BrFNNa [M+Na]^+^ 221.9331, found: 221.9327.

#### 3.1.3. Procedure for Synthesis of **14**

To a solution of 3-bromo-2-fluorobenzonitrile (**13**, 42.0 g, 102 mmol) in isopropanol (100 mL) and water (30 mL) was added with hydrazine hydrate (20 wt% in water, 89 kg), and the mixture was heated to about 80 °C for about 4 h. Upon completion, as confirmed by TLC, the reaction mixture was filtered, and the filtered cake was washed with a mixture of isopropanol (500 mL) and water (2 × 200 mL). The filtrate was concentrated under reduced pressure to afford 7-bromo-*1H*-indazol-3-amine as brown oil (**14**, 32 g, 60%). ^1^H NMR (400 MHz, C*D*Cl_3_) *δ* 9.56 (s, 1H), 8.01 (s, 1H), 7.85 (ddd, *J* = 8.0, 6.3, 1.6 Hz, 1H), 7.57 (ddd, *J* = 8.1, 6.6, 1.6 Hz, 1H), 7.07 (t, *J* = 7.9 Hz, 1H). ^13^C NMR (100 MHz, C*D*Cl_3_) *δ* 173.7, 158.8, 156.3, 135.7, 135.7, 134.7, 125.7, 125.7, 125.3, 125.3, 123.3, 123.2, 109.8, 109.6, 77.2. HRMS (ESI+) *m/z* calcd for C_7_H_7_BrN_3_ [M+H]^+^ 211.9823, found: 211.9819. 

#### 3.1.4. Procedure for Synthesis of **7**

To a solution of 7-bromo-1*H*-indazol-3-amine (**14**, 73 mg, 0.44 mmol, 1.00 equiv.) in 1,4-dioxane (2.20 mL) was added KOAc (86.65 mg, 1.77 mmol, 4.00 equiv.), 4,4,5,5-tetramethyl-2-(tetramethyl-1,3,2-dioxaborolan-2-yl)-1,3,2-dioxaborolane (168.10 mg, 1.32 mmol, 3.00 equiv.), and Pd(PPh_3_)_2_Cl_2_ (15.50 mg, 0.044 mmol, 0.1 equiv.). The mixture was heated to about 110 °C overnight. Upon completion, as confirmed by TLC, the reaction mixture was cooled to room temperature and quenched by the addition of iced water followed by EtOAc (∼100 mL) was added. The combined organic layer was further washed with water and brine, dried over Na_2_SO_4_, and concentrated. The crude product was purified by Combi-flash on silica get using 2–15% Hexane/EtOAc to afford 7-(4,4,5,5-tetramethyl-1,3,2-dioxaborolan-2-yl)-1*H*-indazol-3-amine intermediate as yellowish solid (**7**, 42.00 mg, 50%). ^1^H NMR (600 MHz, DMSO-*d*_6_) *δ* 7.56 (td, *J* = 7.5, 2.1 Hz, 1H), 7.49 (ddd, *J* = 7.5, 5.7, 2.0 Hz, 1H), 7.15 (t, *J* = 7.4 Hz, 1H), 5.20 (t, *J* = 5.7 Hz, 1H), 4.50 (d, *J* = 5.7 Hz, 3H), 1.26 (s, 12H). ^13^C NMR (100 MHz, C*D*Cl_3_) *δ* 166.1, 163.6, 159.4, 136.2, 136.2, 132.6, 132.5, 132.5, 128.1, 128.1, 127.7, 127.6, 123.9, 123.8, 123.6, 123.6, 77.3, 75.1, 60.8, 60.7, 59.5, 59.4, 59.4, 59.3, 59.3, 59.1, 30.9, 25.0, 24.8, 24.6, 24.5, 20.9. HRMS (ESI+) *m/z* calcd for C_13_H_19_BrN_3_O_2_ [M+H]^+^ 338.1346, found: 338.1337.

#### 3.1.5. Procedure for Synthesis of **16**

To a stirred solution of 2,5-dibromopyridine (**15**, 1 g, 1.0 equiv.) in dry THF (1.0 L), was added a nitrogen gas balloon. Separately, 2,2,6,6-tetramethylpiperidinylmagnesium chloride and lithium chloride complex (TMPMgCl.LiCI) (5.8 mL, 6.3 mmol) was added to a round bottom flask. The TMPMgCl.LiCl solution was agitated and cooled to about −20 °C. Then, compound **15** solution was added to the TMPMgCl.LiCl solution over about 30 min, maintaining a temperature below about −20 °C. Upon completing the addition, the flask was maintained at about −20 for about 1 h. A solution of dry-dimethylformamide (1.6 mL, 20 mmol) in THF (1.6 mL) was added to the mixture over about 30 min. The reaction mixture was stirred for a further 15 min. and quenched by the addition of a solution of acetic acid (1.9 mL, 34 mmol) in water (10 mL) over about 30 min, maintaining a temperature of about 0 °C. To the flask was added isopropyl acetate (10 mL) and the mixture was allowed to room temperature for 30 min, the mixture was filtered through celite and rinsed with a mixture of isopropyl acetate (10 mL), saturated ammonium chloride and 0.2 M hydrochloric acid. The pH of the combined reaction mixture was adjusted to about 8–9 by the addition of a 10% aqueous sodium hydroxide solution. The mixture was filtered a second time to remove magnesium salts and transferred to a separatory funnel. The phases were separated, and the aqueous phase was extracted with isopropyl acetate. The combined organic extracts were washed with brine, dried over Na_2_SO_4_, and concentrated. The crude product was purified by Combi-flash on silica gel using 0–70% Hexane/EtOAc to afford 3,6-dibromopicolinaldehyde intermediate as orange solid (**16**, 97.9 g, 56%).^1^H NMR (400 MHz, C*D*Cl_3_) *δ* 10.02 (s, 1H), 7.80 (d, *J* = 8.4 Hz, 1H), 7.47 (d, *J* = 8.5 Hz, 1H). ^13^C NMR (100 MHz, C*D*Cl_3_) *δ* 188.1, 150.2, 140.1, 131.8, 128.4, 118.9, 76.4. HRMS (ESI+) *m/z* calcd for C_6_H_4_BrNO [M+H]^+^ 263.8659, found: 263.8651.

#### 3.1.6. Procedure for Synthesis of **17**

To a stirred solution of 3,6-dibromopicolinaldehyde (**16**, 0.20 g, 0.38 mol, 1.00 equiv.) was added Cs_2_CO_3_ (0.29 g, 0.453 mmol), (*S*)-(+)-*tert*-butanesulfinamide (0.10 g, 0.415 mmol) in DCM (5 mL) at room temperature. The reaction mixture was stirred for 2 h in the same condition. Upon completion, as confirmed by TLC, DCM (∼100 mL) was added to extract. The combined organic layer was further washed with water and brine, dried over Na_2_SO_4_, and concentrated in vacuo. The crude product was purified by Combi-flash on silica get using 5–25% Hexane/EtOAc to afford (*S*)-*N*-((3,6-dibromopyridin-2-yl)methylene)-2-methylpropane-2-sulfinamide intermediate (**17**, 9.91 g, 76%). ^1^H NMR (400 MHz, C*D*Cl_3_) *δ* 8.87 (s, 1H), 7.83 (d, *J* = 8.4 Hz, 1H), 7.44 (d, *J* = 8.4 Hz, 1H), 1.31 (s, 9H). ^13^C NMR (100 MHz, C*D*Cl_3_) *δ* 151.2, 142.5, 141.1, 140.8, 140.4, 132.8, 129.4, 124.0, 119.9, 114.5, 77.2, 30.9. HRMS (ESI-) *m/z* calcd for C_10_H_11_Br_2_N_2_OS [M−H]^−^ 364.8959, found 364.8953.

#### 3.1.7. Procedure for Synthesis of **18**

To a stirred solution of 3,6-dibromopicolinaldehyde (**16**, 0.20 g, 0.38 mol, 1.00 equiv.) was added Cs_2_CO_3_ (0.29 g, 0.453 mmol), (*R*)-(-)-*tert*-butanesulfinamide (0.11 g, 0.415 mmol) in DCM (5 mL) at room temperature. The reaction mixture was stirred for 2 h in the same condition. Upon completion, as confirmed by TLC, DCM (∼100 mL) was added for extraction. The combined organic layer was further washed with water and brine, dried over Na_2_SO_4_ and concentrated in vacuo. The crude product was purified by Combi-flash on silica get using 5–25% Hexane/EtOAc to afford (*R*)-*N*-((3,6-dibromopyridin-2-yl)methylene)-2-methylpropane-2-sulfinamide as yellow solid, 82.5 g (**18**, 60%). ^1^H NMR (400 MHz, C*D*Cl_3_) *δ* 8.80 (s, 1H), 7.76 (d, *J* = 8.4 Hz, 1H), 7.37 (d, *J* = 8.3 Hz, 1H), 1.24 (s, 9H). ^13^C NMR (100 MHz, C*D*Cl_3_) *δ* 206.9, 160.0, 149.6, 144.8, 143.9, 141.0, 132.8, 131.2, 128.4, 121.9, 77.3, 58.8, 58.6, 58.4, 32.3, 31.6, 31.2, 30.9, 24.2, 23.8, 22.9, 22.7, 21.6. HRMS (ESI-) *m/z* calcd for C_10_H_11_Br_2_N_2_OS [M−H]^−^ 364.8959, found 364.8947. 

### 3.2. General Procedure for Synthesis of ***19a****,****b*** and ***20a****,****b***

To a solution of (*S*)-*N*-((3,6-dibromopyridin-2-yl)methylene)-2-methylpropane-2-sulfinamide intermediate (**17**, 11.41 g, 15.51 mol, 1.0 equiv.) in dry THF (150 mL) was added (3,5-difluorobenzyl)zinc bromide (**25**, 0.5 M in THF, 125 mL, 31.01 mmol) or benzyl magnesium chloride (**24**, 0.5 M in THF, 125 mL, 31.01 mmol) dropwise by additional funnel for about 30 min at 0 °C. The reaction mixture was warmed to room temperature and stirred at that temperature for 2 h. Upon completion, as confirmed by TLC, the mixture was quenched with saturated NH_4_Cl (~200 mL) and diluted with EtOAc (~200 mL), and then it was washed with water (~0.5 L) and then brine (~0.5 L). The organic solution was dried over Na_2_SO_4_, filtered, and then concentrated in vacuo. The crude product was purified by Combi-flash on silica gel using 5–50% Hexane/EtOAc to afford methanesulfonamide intermediate (**19a**,**b**, **20a**,**b**, 10.21 g, 65%).

#### 3.2.1. (*S*)-N-((*R*)-1-(3,6-Dibromopyridin-2-yl)-2-phenylethyl)-2-methylpropane-2-sulfinamide (**19a**)

Yield: 65%. ^1^H NMR (400 MHz, C*D*Cl_3_) *δ* 7.11 (s, 1H), 7.09 (s, 1H), 7.00 (d, *J* = 7.4 Hz, 2H), 6.96 (d, *J* = 7.2 Hz, 3H), 5.00 (s, 1H), 4.51 (d, *J* = 7.7 Hz, 1H), 4.38 (d, *J* = 8.6 Hz, 1H), 3.20 (d, *J* = 7.8 Hz, 2H), 1.05 (d, *J* = 1.6 Hz, 9H). ^13^C NMR (100 MHz, C*D*Cl_3_) *δ* 159.1, 150.1, 149.8, 141.6, 140.7, 140.0, 139.7, 139.3, 138.8, 136.3, 129.7, 128.7, 128.4, 128.1, 127.9, 127.4, 127.3, 127.0, 126.8, 126.3, 125.6, 125.1, 124.9, 118.9, 76.3, 42.1, 36.9, 29.3, 29.3. HRMS (ESI+) *m/z* calcd for C_17_H_20_Br_2_N_2_OSNa [M+Na]^+^ 480.9561, found 480.9553. 

#### 3.2.2. (*S*)-N-((*S*)-1-(3,6-Dibromopyridin-2-yl)-2-phenylethyl)-2-methylpropane-2-sulfinamide (**20a**)

Yield: 65%. ^1^H NMR (400 MHz, C*D*Cl_3_) *δ* 7.55–7.40 (m, 1H), 7.19–7.07 (m, 1H), 3.56–3.46 (m, 3H), 2.92 (d, *J* = 11.8 Hz, 10H). HRMS (ESI+) *m/z* calcd for C_17_H_20_Br_2_N_2_OSNa [M+Na]^+^ 480.9561, found 480.9555.

#### 3.2.3. (*S*)-N-((*R*)-1-(3,6-Dibromopyridin-2-yl)-2-(3,5-difluorophenyl)ethyl)-2-methylpropane-2-sulfinamide (**19b**)

Yield: 65%. ^1^H NMR (400 MHz, C*D*Cl_3_) *δ* 7.68–7.59 (m, 1H), 7.21–7.14 (m, 1H), 6.82 (h, *J* = 4.0 Hz, 2H), 6.62 (tt, *J* = 9.1, 2.4 Hz, 1H), 4.61 (s, 2H), 3.93 (s, 1H), 3.01 (d, *J* = 2.2 Hz, 1H), 1.31–1.29 (m, 9H). ^13^C NMR (100 MHz, C*D*Cl_3_) *δ* 164.0, 163.9, 161.5, 161.4, 159.2, 155.8, 142.8, 140.6, 140.5, 140.4, 140.1, 128.4, 119.2, 112.5, 112.4, 112.3, 112.2, 102.4, 102.1, 101.9, 77.2, 53.6, 41.1, 41.1, 41.1. HRMS (ESI-) *m/z* calcd for C_17_H_17_Br_2_F_2_N_2_OS [M−H]^−^ 492.9397, found 492.9381.3.5.4. (*S*)-N-((*S*)-1-(3,6-dibromopyridin-2-yl)-2-(3,5-difluorophenyl)ethyl)-2-methylpropane-2-sulfinamide (**20b**).

Yield: 65%. ^1^H NMR (400 MHz, C*D*Cl_3_) *δ* 7.32–7.26 (m, 3H), 7.10 (d, *J* = 7.7 Hz, 2H), 6.96 (d, *J* = 7.2 Hz, 5H), 6.88 (d, *J* = 7.4 Hz, 1H), 5.00 (s, 1H), 4.51 (d, *J* = 7.8 Hz, 1H), 4.38 (d, *J* = 8.5 Hz, 1H), 3.20 (d, *J* = 8.3 Hz, 4H), 1.01 (s, 9H). HRMS (ESI-) *m/z* calcd for C_17_H_17_Br_2_ F_2_N_2_OS [M−H]^−^ 492.9397, found 492.9390. 

### 3.3. General Procedure for Synthesis of ***21a****,****b*** and ***22a****,****b***

To a solution of (*R*)-*N*-((3,6-dibromopyridin-2-yl)methylene)-2-methylpropane-2-sulfinamide (**18,** 11.41 g, 15.51 mol, 1.0 equiv.) in dry THF (150 mL) was added (3,5-difluorobenzyl)zinc bromide (**25**, 0.5 M in THF, 125 mL, 31.01 mmol) or Benzyl magnesium chloride (**24**, 0.5 M in THF, 125 mL, 31.01 mmol) dropwise by additional funnel for about 30 min at 0 °C. The reaction mixture was warmed to room temperature and stirred at that temperature for 2 h. Upon completion, as confirmed by TLC, the mixture was quenched with saturated NH_4_Cl (~200 mL) and diluted with EtOAc (~200 mL) and then was washed with water (~0.5 L), and then brine (~0.5 L). The organic solution was dried over Na_2_SO_4_; filtered, and then concentrated in vacuo. The crude product was purified by Combi-flash on silica gel using 5–50% Hexane/EtOAc to afford methanesulfonamide intermediate (**21a**,**b** and **22a**,**b**, 28.36 g, 86%).

#### 3.3.1. (*R*)-N-((*R*)-1-(3,6-Dibromopyridin-2-yl)-2-phenylethyl)-2-methylpropane-2-sulfinamide (**21a**)

Yield: 65%. ^1^H NMR (400 MHz, C*D*Cl_3_) *δ* 7.50 (d, *J* = 8.3 Hz, 1H), 7.33–7.19 (m, 1H), 7.17–7.12 (m, 3H), 7.03–6.99 (m, 2H), 6.91 (d, *J* = 7.5 Hz, 1H), 5.08 (q, *J* = 7.5 Hz, 1H), 4.76 (d, *J* = 9.4 Hz, 1H), 4.19 (d, *J* = 8.9 Hz, 1H), 3.95–3.90 (m, 1H), 3.17 (d, *J* = 7.2 Hz, 1H), 1.04 (d, *J* = 13.3 Hz, 9H). ^13^C NMR (100 MHz, C*D*Cl_3_) *δ* 163.9, 161.3, 158.4, 155.9, 155.0, 141.1, 128.8, 124.4, 111.9, 101.7, 101.4, 87.3, 85.5, 76.8, 58.0, 52.8, 35.2, 28.3, 24.8, 22.8. HRMS (ESI-) *m/z* calcd for C_17_H_19_Br_2_N_2_OS [M−H]^−^ 456.9585, found 456.9576. 

#### 3.3.2. (*R*)-N-((*S*)-1-(3,6-Dibromopyridin-2-yl)-2-phenylethyl)-2-methylpropane-2-sulfinamide (**22a**)

Yield: 65%. ^1^H NMR (400 MHz, C*D*Cl_3_) *δ* 7.28 (d, *J* = 8.0 Hz, 1H), 7.17 (s, 1H), 7.12 (d, *J* = 7.3 Hz, 2H), 7.00 (d, *J* = 7.3 Hz, 2H), 6.96 (d, *J* = 7.2 Hz, 3H), 5.00 (s, 1H), 4.51 (d, *J* = 7.8 Hz, 1H), 4.38 (d, *J* = 8.5 Hz, 1H), 3.20 (d, *J* = 8.3 Hz, 3H), 1.05 (s, 9H). HRMS (ESI-) *m/z* calcd for C_17_H_19_Br_2_N_2_OS [M−H]^−^ 456.9585, found 456.9578. 

#### 3.3.3. (*R*)-N-((*R*)-1-(3,6-Dibromopyridin-2-yl)-2-(3,5-difluorophenyl)ethyl)-2-methylpropane-2-sulfinamide (**21b**)

Yield: 65%. ^1^H NMR (400 MHz, C*D*Cl_3_) *δ* 7.50 (dd, *J* = 8.3, 1.7 Hz, 1H), 7.19–7.12 (m, 3H), 7.03–6.99 (m, 2H), 6.91 (d, *J* = 8.0 Hz, 1H), 5.12–5.04 (m, 1H), 4.77 (d, *J* = 9.6 Hz, 1H), 4.20 (d, *J* = 8.9 Hz, 1H), 3.17 (d, *J* = 7.1 Hz, 2H), 1.05 (d, *J* = 1.7 Hz, 9H). HRMS (ESI-) *m/z* calcd for C_17_H_17_Br_2_F_2_N_2_OS [M−H]^−^ 492.9397, found 492.9389.

#### 3.3.4. (*R*)-N-((*S*)-1-(3,6-Dibromopyridin-2-yl)-2-(3,5-difluorophenyl)ethyl)-2-methylpropane-2-sulfinamide (**22b**)

Yield: 65%. ^1^H NMR (400 MHz, C*D*Cl_3_) *δ* 7.28 (d, *J* = 8.0 Hz, 1H), 7.17 (s, 1H), 7.12 (d, *J* = 7.3 Hz, 2H), 7.00 (d, *J* = 7.3 Hz, 2H), 6.96 (d, *J* = 7.2 Hz, 3H), 5.00 (s, 1H), 4.51 (d, *J* = 7.8 Hz, 1H), 4.38 (d, *J* = 8.5 Hz, 1H), 3.20 (d, *J* = 8.3 Hz, 3H), 1.05 (s, 9H). HRMS (ESI-) *m/z* calcd for C_17_H_17_Br_2_F_2_N_2_OS [M−H]^−^ 492.9397, found 492.9387. 

### 3.4. General Procedure for Synthesis of ***23a****,****b***

To a solution of (*S*)-N-((*S*)*-*1-(3,6-dibromopyridin-2-yl)-2-phenylethyl)-2-methylpropane-2-sulfinamide (**20a**, 6.62 g, 13.34 mol, 1.0 equiv.) or (*S*)-N-((*S*)-1-(3,6-dibromopyridin-2-yl)-2-(3,5-difluorophenyl)ethyl)-2-methylpropane-2-sulfinamide (**22b**, 6.62 g, 13.34 mol, 1.0 equiv.) in MeOH (30 mL) at 0 °C was added 4N HCl in EtOAc (7.50 mL) at room temperature for about 2 h. Upon completion, as confirmed by TLC, the reaction mixture was formed a thick slurry and filtered and washed with MeOH to get deprotected methanesulfonamide intermediate (**23a**,**b**, 6.05 g, 76%), which was used directly in the next step.

#### 3.4.1. (*S*)-1-(3,6-Dibromopyridin-2-yl)-2-phenylethan-1-amine (**23a**)

Yield: 76%. ^1^H NMR (600 MHz, C*D*Cl_3_) *δ* 7.57 (d, *J* = 8.3 Hz, 1H), 7.23 (d, *J* = 8.5 Hz, 2H), 7.09 (d, *J* = 8.0 Hz, 1H), 6.95–6.91 (m, 2H), 5.18 (s, 1H), 3.95 (s, 1H), 3.73 (s, 1H), 3.31 (dd, *J* = 13.8, 7.1 Hz, 1H), 3.26–3.19 (m, 1H). ^13^C NMR (100 MHz, DMSO-*d*_6_) *δ* 209.9, 154.9, 144.1, 140.2, 134.6, 130.3, 129.7, 129.2, 129.0, 127.9, 120.5, 54.1, 31.0, 25.6. HRMS (ESI+) *m/z* calcd for C_13_H_13_Br_2_N_2_ [M+H]^+^ 354.9445, found 354.9437. 

#### 3.4.2. (*S*)-1-(3,6-Dibromopyridin-2-yl)-2-(3,5-difluorophenyl)ethan-1-amine (**23b**)

Yield: 76%. ^1^H NMR (600 MHz, C*D*Cl_3_) *δ* 7.65 (d, *J* = 8.3 Hz, 1H), 7.31 (d, *J* = 8.2 Hz, 1H), 6.65 (dd, *J* = 28.3, 7.9 Hz, 3H), 5.25 (s, 1H), 3.33 (dd, *J* = 13.4, 7.5 Hz, 1H), 2.68 (s, 1H). ^13^C NMR (100 MHz, C*D*Cl_3_) *δ* 164.2, 164.1, 161.7, 161.6, 153.9, 143.0, 140.3, 137.9, 137.8, 137.7, 129.7, 120.0, 113.1, 113.0, 112.9, 112.8, 103.3, 103.1, 102.8, 77.2, 54.5, 53.9, 39.0, 31.6, 25.1, 23.8. HRMS (ESI+) *m/z* calcd for C_13_H_11_Br_2_F_2_N_2_ [M+H]^+^ 390.9257, found 390.9248. 

### 3.5. General Procedure for Synthesis of ***8a****,****b***

To a solution of (*S*)-1-(3,6-dibromopyridin-2-yl)-2-phenylethan-1-amine (**23a**, 6.30 g, 0.12 mol, 1.0 equiv.) or (*S*)-1-(3,6-dibromopyridin-2-yl)-2-(3,5-difluorophenyl)ethan-1-amine (**18b**, 6.00 g, 0.12 mol, 1.0 equiv.) in DCM (100 mL) at room temperature was added triethylamine (3.7 mL, 26.68 mmol) and di-*tert*-butyl dicarbonate (3.5 g, 16.01 mmol). The mixture was stirred at room temperature for 4 h. Upon completion, as confirmed by TLC, diluted with ethyl acetate, then the combined organic layer was further washed with water and brine, dried over Na_2_SO_4_ and concentrated. The crude product was purified by Combi-flash on silica get using 10–80% hexane/EtOAc to afford boc group protected methanesulfonamide intermediate (**8a**,**b**, 6.00 g, 80%).

#### 3.5.1. *Tert*-butyl (*S*)-(1-(3,6-dibromopyridin-2-yl)-2-phenylethyl)carbamate (**8a**)

Yield: 80%. ^1^H NMR (600 MHz, C*D*Cl_3_) *δ* 7.66–7.55 (m, 1H), 7.37–7.28 (m, 1H), 7.24–7.18 (m, 1H), 7.17–7.08 (m, 1H), 7.03 (s, 1H), 4.94 (dd, *J* = 12.7, 6.5 Hz, 1H), 4.79–4.71 (m, 1H), 3.25–3.10 (m, 1H), 3.02–2.92 (m, 1H), 1.23 (s, 9H). ^13^C NMR (100 MHz, C*D*Cl_3_) *δ* 171.2, 151.2, 142.7, 141.1, 140.2, 136.3, 129.8, 128.7, 127.0, 126.6, 119.4, 78.5, 75.1, 60.4, 55.5, 24.9. HRMS (ESI+) *m/z* calcd for C_18_H_21_Br_2_N_2_O_2_ [M+H]^+^ 454.9970, found 454.9961. 

#### 3.5.2. Tert-butyl (S)-(1-(3,6-dibromopyridin-2-yl)-2-(3,5-difluorophenyl)ethyl)carbamate (**8b**)

Yield: 80%. ^1^H NMR (400 MHz, C*D*Cl_3_) *δ* 7.74–7.62 (m, 1H), 7.47–7.40 (m, 1H), 6.91–6.84 (m, 2H), 5.70–5.48 (m, 2H), 3.41 (s, 0H), 3.16–2.85 (m, 1H), 1.45 (s, 9H). ^13^C NMR (100 MHz, C*D*Cl_3_) *δ* 164.1, 163.9, 161.6, 161.5, 158.9, 154.9, 142.7, 140.7, 140.6, 140.5, 140.2, 128.4, 119.0, 112.5, 112.5, 112.4, 112.3, 102.4, 102.2, 101.9, 79.9, 77.2, 65.7, 57.1, 54.1, 41.5, 28.5, 28.3, 27.4, 21.6, 15.8. HRMS (ESI+) *m/z* calcd for C_18_H_18_Br_2_F_2_N_2_O_2_Na [M+Na]^+^ 514.9575, found 514.9578. 

### 3.6. Procedure for Synthesis of ***21***

To a solution of MeSO_2_Na (**26**, 6 g, 29.26 mmol, 1.0 equiv.) in DMF (100 mL) was added copper(I) chloride (0.48 g, 2.4 mmol) slowly. The reaction mixture was heated to 40 °C and maintained at that temperature for 18 h. Upon completion, as confirmed by TLC, the reaction mixture was cooled to room temperature and concentrated under reduced pressure. The resulting residue was diluted with water and extracted with EtOAc to remove unreacted starting material. The aqueous layer was acidified with 0.5 M citric acid and followed by 1 N NaOH. The combined organic layers were washed with water, brine (1.0 L); dried over Na_2_SO_4_; filtered and then concentrated in vacuo. The crude product was purified by Combi-flash on silica get using 0–5% Hexane/EtOAc to afford 3-methyl-3-(methylsulfonyl)but-1-yne (**21**, 2.92 g, 41%). ^1^H NMR (600 MHz, C*D*Cl_3_) *δ* 3.04 (s, 3H), 2.58 (d, *J* = 1.5 Hz, 1H), 1.67 (s, 6H). ^13^C NMR (100 MHz, C*D*Cl_3_) *δ* 159.2, 70.2, 58.0, 54.5, 35.3, 25.5, 23.7, 22.6. HRMS (ESI+) *m/z* calcd for C_6_H_10_O_2_SNa [M+Na]^+^ 169.0294, found 169.0289. 

### 3.7. General Procedure for Synthesis of ***27a**,**b***

To a solution of *tert-*butyl (*S*)-(1-(3,6-dibromopyridin-2-yl)-2-phenylethyl)carbamate (**8a**, 0.80 g, 0.813 mmol 1.0 equiv.) or *tert-*butyl (*S*)-(1-(3,6-dibromopyridin-2-yl)-2-(3,5-difluorophenyl)ethyl)carbamate (**8b**, 0.80 g, 0.813 mmol 1.0 equiv.), 3-methyl-3-(methylsulfonyl)but-1-yne (**10**, 0.286 g, 0.975 mmol), in DMF (10 mL) was added triethyl amine (0.70 mL, 2.44 mmol). At the room temperature, Bis(triphenylphosphine)palladium(II)dichloride (70 mg, 0.0406 mmol) and copper(I) iodide (15.45 mg, 0.0406 mmol) were added to the reaction mixture and was stirred at room temperature for another 6 h. Upon completion, confirmed by TLC, the reaction mixture was concentrated under reduced pressure. The resulting residue was diluted with water and extracted with EtOAc to remove unreacted starting material. The combined organic layers were washed with water, brine; dried over Na_2_SO_4_; filtered and then concentrated in vacuo. The crude product was purified by Combi-flash on silica get using 20–50% Hexane/EtOAc to afford carbamate intermediate (**27a**,**b**, 0.59 g, 65%).

#### 3.7.1. *Tert*-butyl (*S*)-(1-(3-bromo-6-(3-methyl-3-(methylsulfonyl)but-1-yn-1-yl)pyridin-2-yl)-2-phenylethyl)carbamate (**27a**)

Yield: 65%. ^1^H NMR (400 MHz, C*D*Cl_3_) *δ* 7.81 (d, *J* = 8.1 Hz, 1H), 7.66 (d, *J* = 8.3 Hz, 1H), 7.42 (d, *J* = 18.1 Hz, 1H), 7.23 (d, *J* = 8.3 Hz, 1H), 6.62 (s, 3H), 5.69 (d, *J* = 8.7 Hz, 1H), 5.57 (s, 1H), 3.11 (s, 4H), 2.90 (d, *J* = 14.5 Hz, 1H), 1.80 (s, 6H), 1.55 (s, 5H), 1.41 (s, 9H). ^13^C NMR (100 MHz, C*D*Cl_3_) *δ* 189.3, 160.0, 149.6, 148.9, 144.8, 143.9, 141.1, 141.0, 132.8, 131.2, 122.0, 77.2, 58.6, 31.6, 24.5, 22.8. HRMS (ESI-) *m/z* calcd for C_24_H_28_BrN_2_O_4_S [M−H]^−^ 521.1031, found 521.1028. 

#### 3.7.2. *Tert*-butyl (*S*)-(1-(3-bromo-6-(3-methyl-3-(methylsulfonyl)but-1-yn-1-yl)pyridin-2-yl)-2-(3,5-difluorophenyl)ethyl)carbamate (**27b**)

Yield: 65%. ^1^H NMR (400 MHz, MeOD) δ 7.81 (d, J = 8.2 Hz, 1H), 7.23 (d, J = 8.2 Hz, 1H), 6.63 (d, J = 16.8 Hz, 2H), 5.70 (d, J = 9.0 Hz, 1H), 5.50 (d, J = 7.7 Hz, 1H), 3.12 (d, J = 7.7 Hz, 3H), 2.93–2.87 (m, 1H), 1.80 (s, 7H), 1.56 (s, 9H), 1.43 (d, J = 7.4 Hz, 8H). ^13^C NMR (100 MHz, CDCl_3_) δ 164.0, 161.6, 159.0, 155.0, 142.7, 140.6, 140.2, 128.4, 119.1, 112.5, 112.5, 112.4, 112.3, 102.4, 102.2, 101.9, 79.9, 77.2, 54.1, 45.8, 41.5, 28.3, 8.7. HRMS (ESI-) *m/z* calcd for C_24_H_26_BrF_2_N_2_O_4_S [M−H]^−^ 555.0763, found 555.0757. 

### 3.8. General Procedure for Synthesis of ***28a**,**b***

To a solution of *tert-*butyl (*S*)*-*(1-(3-bromo-6-(3-methyl-3-(methylsulfonyl)but-1-yn-1-yl)pyridin-2-yl)-2-phenylethyl)carbamate (**27a**, 228 g, 0.21 mmol) or *tert*-butyl (*S*)-(1-(3-bromo-6-(3-methyl-3-(methylsulfonyl)but-1-yn-1-yl)pyridin-2-yl)-2-(3,5-difluorophenyl)ethyl)carbamate (**27b**, 228 g, 0.21 mmol) in 1,4-dioxane (4 mL) and water (0.4 mL) were added [1,1′*bis*(diphenylphosphino)-ferrocene]dichloropalladium(II) (35.0 mg, 0.0205 mmol), potassium carbonate (170.0 mg, 1.35 mmol) and 7-(4,4,5,5-*tetra*methyl-1,3,2-dioxaborolan-2-yl)-*1H*-indazol-3-amine (**7**, 200 mg, 0.266 mmol) at room temperature. The red solution was stirred in a heated condition at 110 °C for about 4 h. Upon completion, as confirmed by TLC, the solution was concentrated under reduced pressure, and the resulting residue was dissolved in EtOAc for extraction. The combined organic layers were washed with water, brine; dried over Na_2_SO_4_; filtered and then concentrated in vacuo. The crude product was purified by Combi-flash on silica get using 40–90% Hexane/EtOAc to afford the desired intermediate (**28a**,**b**, 0.10 g, 51%).

#### 3.8.1. *Tert*-butyl (*S*)-(1-(3-(3-amino-1H-indazol-7-yl)-6-(3-methyl-3-(methylsulfonyl)but-1-yn-1-yl)pyridin-2-yl)-2-phenylethyl)carbamate (**28a**)

Yield: 61%. ^1^H NMR (400 MHz, C*D*Cl_3_) *δ* 7.56–7.46 (m, 1H), 7.40 (d, *J* = 7.7 Hz, 2H), 7.03–6.89 (m, 1H), 6.51 (t, *J* = 8.6 Hz, 2H), 6.15 (d, *J* = 7.6 Hz, 3H), 5.52 (s, 2H), 4.94–4.70 (m, 7H), 4.56 (s, 1H), 4.12 (q, *J* = 7.1 Hz, 1H), 2.84–2.66 (m, 2H), 2.01–1.95 (m, 8H), 1.41 (s, 9H). HRMS (ESI-) *m/z* calcd for C_31_H_34_N_5_O_4_S [M−H]^−^ 572.2332, found 572.2326. 

#### 3.8.2. *Tert*-butyl (*S*)-(1-(3-(3-amino-1H-indazol-7-yl)-6-(3-methyl-3-(methylsulfonyl)but-1-yn-1-yl)pyridin-2-yl)-2-(3,5-difluorophenyl)ethyl)carbamate (**28b**)

Yield: 61%. ^1^H NMR (400 MHz, C*D*Cl_3_) *δ* 7.70–7.64 (m, 2H), 7.55 (td, *J* = 7.3, 1.5 Hz, 1H), 7.49–7.40 (m, 4H), 7.24 (d, *J* = 7.7 Hz, 1H), 6.54 (t, *J* = 9.3 Hz, 1H), 6.17 (s, 2H), 5.65 (d, *J* = 9.4 Hz, 1H), 4.83 (d, *J* = 6.0 Hz, 1H), 4.48 (s, 1H), 3.16 (s, 3H), 2.93 (s, 3H), 1.84 (s, 7H), 1.24 (s, 9H). ^13^C NMR (100 MHz, C*D*Cl_3_) *δ* 164.1, 164.0, 161.6, 161.5, 159.0, 154.9, 142.7, 140.7, 140.6, 140.5, 140.2, 128.4, 119.0, 112.5, 112.5, 112.4, 112.3, 102.4, 102.2, 101.9, 79.9, 77.2, 65.7, 57.1, 54.1, 41.5, 28.5, 28.3, 27.4, 21.6, 15.8. HRMS (ESI-) *m/z* calcd for C_31_H_32_F_2_N_5_O_4_S [M−H]^−^ 608.2143, found 608.2137. 

### 3.9. General Procedure for Synthesis of ***29a**,**b***

To a solution of *tert*-butyl (*S*)-(1-(3-(3-amino-*1H*-indazol-7-yl)-6-(3-methyl-3-(methylsulfonyl)but-1-yn-1-yl)pyridin-2-yl)-2-phenylethyl)carbamate (**28a**, 0.10 g, 0.14 mmol, 1.0 equiv.) or *tert*-butyl (*S*)-(1-(3-(3-amino-*1H*-indazol-7-yl)-6-(3-methyl-3-(methylsulfonyl)but-1-yn-1-yl)pyridin-2-yl)-2-(3,5-difluorophenyl)ethyl)carbamate (**28b**, 0.1 g, 0.14 mmol, 1.0 equiv.) in 3 mL of DCM and triethylamine (80 μL, 0.0486 mmol) was added methanesulfonyl chloride (0.2 g, 1.05 equiv.), and the reaction mixture was stirred for 2 h at room temperature. Upon completion, as confirmed by TLC, the reaction mixture was concentrated, diluted with water, and extracted with ethyl acetate. The combined organic layer was further washed with water and brine, dried over Na_2_SO_4_ and concentrated. The crude product was purified by Combi-flash on silica get using 15–35% EtOAc/Hexane to afford desired intermediate as yellow solid (**29a**,**b**, 3.34 g, 73%).

#### 3.9.1. *Tert*-butyl (*S*)-(1-(6-(3-methyl-3-(methylsulfonyl)but-1-yn-1-yl)-3-(3-(N-(methylsulfonyl)methylsulfonamido)-1H-indazol-7-yl)pyridin-2-yl)-2-phenylethyl)carbamate (**29a**)

Yield: 73%. ^1^H NMR (400 MHz, C*D*Cl_3_) *δ* 7.60 (t, *J* = 8.7 Hz, 2H), 7.47 (t, *J* = 7.0 Hz, 1H), 7.17–6.99 (m, 5H), 6.92–6.84 (m, 1H), 5.55 (d, *J* = 9.0 Hz, 1H), 5.47 (d, *J* = 7.5 Hz, 1H), 4.10 (d, *J* = 4.2 Hz, 2H), 3.99 (dd, *J* = 28.7, 6.9 Hz, 2H), 3.78–3.55 (m, 1H), 3.11 (dd, *J* = 13.5, 5.9 Hz, 1H), 2.94 (dd, *J* = 13.5, 7.4 Hz, 1H), 1.56–1.43 (m, 6H). ^13^C NMR (100 MHz, C*D*Cl_3_) *δ* 159.8, 155.0, 144.4, 143.2, 142. 6, 142.1, 140.0, 136.6, 135.8, 132. 8, 132.4, 132.1, 132.0, 129.9, 129.6, 129.3, 129.3, 129.2, 129.2, 129.1, 128.8, 128.7, 128.5, 128.4, 128.3, 128.3, 128.2, 128.0, 127.8, 127.7, 127.6, 127.0, 126.6, 126.4, 123.8, 119.2, 114.6, 82.3, 79.7, 68.7, 54.5, 41.7, 41.5, 41.0, 28.6, 28.3, 27.8, 27.5, 22.4. HRMS (ESI+) *m/z* calcd for C_33_H_40_N_5_O_8_S_3_ [M+H]^+^ 730.2039, found 730.2035. 

#### 3.9.2. Tert-butyl (S)-(2-(3,5-difluorophenyl)-1-(6-(3-methyl-3-(methylsulfonyl)but-1-yn-1-yl)-3-(3-(N-(methylsulfonyl)methylsulfonamido)-1H-indazol-7-yl)pyridin-2-yl)ethyl)carbamate (**29b**)

Yield: 73%. ^1^H NMR (400 MHz, C*D*Cl_3_) *δ* 7.52–7.43 (m, 2H), 7.27 (d, *J* = 2.4 Hz, 1H), 6.74 (s, 1H), 6.59 (t, *J* = 9.0 Hz, 1H), 6.50 (s, 1H), 6.16 (d, *J* = 7.0 Hz, 1H), 5.90 (d, *J* = 9.1 Hz, 1H), 5.56 (d, *J* = 13.7 Hz, 1H), 4.54 (d, *J* = 13.7 Hz, 1H), 4.12 (qd, *J* = 7.3, 1.8 Hz, 1H), 3.65–3.57 (m, 3H), 3.57–3.50 (m, 4H), 3.16 (s, 3H), 3.14–3.07 (m, 4H), 2.86 (s, 1H), 2.76 (t, *J* = 10.3 Hz, 1H), 1.84 (s, 6H), 1.39 (s, 7H). HRMS (ESI+) *m/z* calcd for C_33_H_38_F_2_N_5_O_8_S_3_ [M+H]^+^ 766.1852, found 766.1845. 

### 3.10. General Procedure for Synthesis of ***30a**,**b***

To a solution of *tert*-butyl (*S*)-(1-(6-(3-methyl-3-(methylsulfonyl)but-1-yn-1-yl)-3-(3-(*N*-(methylsulfonyl)methylsulfonamido)-*1H*-indazol-7-yl)pyridin-2-yl)-2-phenylethyl)carbamate (**29a**, 0.10 g, 0.113 mmol, 1.0 equiv.) or *tert*-butyl (*S*)-(2-(3,5-difluorophenyl)-1-(6-(3-methyl-3-(methylsulfonyl)but-1-yn-1-yl)-3-(3-(*N*-(methylsulfonyl)methylsulfonamido)-*1H*-indazol-7-yl)pyridin-2-yl)ethyl)carbamate (**29b**, 0.10 g, 0.113 mmol, 1.0 equiv.) in 5 mL of dichloromethane was added Trifluoroacetic acid (2.5 mL, 7.7 mmol, 3.0 equiv.) at room temperature, and the reaction mixture was stirred at room temperature for 4 h. Upon completion, as confirmed by TLC, the reaction mixture was carefully neutralized to about pH 7 with a saturated sodium bicarbonate solution and the organic layer was separated. The aqueous layer was further extracted with dichloromethane. The combined organic layer was further washed with water and brine, dried over Na_2_SO_4_, and concentrated to afford desired intermediate as yellow oil (**30a**,**b**, 2.51 g, 50–51%).

#### 3.10.1. (*S*)-N-(7-(2-(1-Amino-2-phenylethyl)-6-(3-methyl-3-(methylsulfonyl)but-1-yn-1-yl)pyridin-3-yl)-1H-indazol-3-yl)-N-(methylsulfonyl)methanesulfonamide (**30a**)

Yield: 51%. ^1^H NMR (400 MHz, C*D*Cl_3_) *δ* 7.52 (d, *J* = 7.2 Hz, 1H), 7.43 (s, 1H), 7.26 (d, *J* = 1.7 Hz, 7H), 7.14 (s, 1H), 6.59 (s, 1H), 6.37 (s, 1H), 6.17 (s, 1H), 4.65 (s, 1H), 3.30 (s, 4H), 3.17 (q, *J* = 4.7 Hz, 8H), 3.04 (s, 1H), 1.83 (s, 6H), 1.44 (s, 6H). HRMS (ESI+) *m/z* calcd for C_28_H_32_N_5_O_6_S_3_ [M+H]^+^ 630.1516, found 630.1510. 

#### 3.10.2. (*S*)-N-(7-(2-(1-Amino-2-(3,5-difluorophenyl)ethyl)-6-(3-methyl-3-(methylsulfonyl)but-1-yn-1-yl)pyridin-3-yl)-1H-indazol-3-yl)-N-(methylsulfonyl)methanesulfonamide (**30b**)

Yield: 51%. ^1^H NMR (400 MHz, C*D*Cl_3_) *δ* 7.52 (d, *J* = 7.4 Hz, 1H), 7.43 (s, 1H), 7.26 (s, 2H), 7.14 (s, 1H), 6.59 (s, 1H), 6.37 (s, 1H), 6.16 (s, 1H), 4.44 (s, 1H), 3.69 (s, 1H), 3.30 (s, 4H), 3.17 (d, *J* = 7.7 Hz, 7H), 3.04 (s, 1H), 1.83 (s, 4H), 1.44 (s, 6H). ^13^C NMR (100 MHz, C*D*Cl_3_) *δ* 164.3, 164.2, 164.0, 161.8, 161.7, 161.6, 158.6, 157.3, 156.1, 141.6, 141.4, 130.4, 130.4, 130.3, 129.3, 128.9, 128.9, 128.8, 128.7, 128.6, 127.8, 124.8, 123.7, 123.7, 123.6, 123.3, 123.3, 123.2, 123.1, 112.1, 111.8, 111.6, 101.8, 101.5, 101.3, 74.5, 64.1, 57.8, 57.5, 38.6, 34.2, 34.1, 33.9, 23.6, 21.7. HRMS (ESI-) *m/z* calcd for C_28_H_29_F_2_N_5_O_6_S_3_ [M−H]^−^ 664.1170, found 664.1164.

### 3.11. General Procedure for Synthesis of ***5a**,**b*** and ***6a**,**b***

To a solution of desired acid (0.45 g, 14.7 mmol, 1.1 equiv.) in 2 mL of DMF was added HATU (0.51 g, 0.13 mmol, 1.05 equiv.) at 0 °C, and the reaction mixture was stirred for 30 min. Followed by addition of a solution of amine intermediate, (*S*)-N-(7-(2-(1-amino-2-phenylethyl)-6-(3-methyl-3-(methylsulfonyl)but-1-yn-1-yl)pyridin-3-yl)-*1H*-indazol-3-yl)-N-(methylsulfonyl)methanesulfonamide (**30a**, 0.30 g, 1.0 equiv.) or (*S*)-N-(7-(2-(1-amino-2-(3,5-difluorophenyl)ethyl)-6-(3-methyl-3-(methylsulfonyl)but-1-yn-1-yl)pyridin-3-yl)-*1H*-indazol-3-yl)-N-(methylsulfonyl)methanesulfonamide (**30b**, 0.30 g, 1.0 equiv.) in 1 mL DMF and DIPEA (0.51 mL, 32.71 mmol, 3.0 equiv.). The reaction mixture was then slowly warmed to room temperature and stirred for 12 h. To the reaction mixture was added a solution of ammonia in MeOH (2 M, 10 mL), and the mixture was then stirred for 10 min. Upon completion, as confirmed by TLC, the reaction mixture was concentrated, diluted with water, and extracted with ethyl acetate and washed with aq 1 M HCl solution. The combined organic layer was further washed with water and brine, dried over Na_2_SO_4_, and concentrated. The crude product was purified by Combi-flash on silica gel using 20–100% DCM/MeOH with 0.1% TFA to remove some impurities. The mixture was concentrated under reduced pressure and the fractions containing each diastereomer were combined and back extracted with EtOAc, dried, and concentrated to afford the final compounds.

#### 3.11.1. 2-(4,7-Dimethyl-2-oxoindolin-3-yl)-N-((*S*)-1-(6-(3-methyl-3-(methylsulfonyl)but-1-yn-1-yl)-3-(3-(methylsulfonamido)-1H-indazol-7-yl)pyridin-2-yl)-2-phenylethyl)acetamide (**6a**)

Yield: 41%. ^1^H NMR (600 MHz, MeO*D*) *δ* 7.65 (dd, *J* = 12.1, 7.7 Hz, 2H), 7.54 (t, *J* = 7.6 Hz, 1H), 7.48–7.43 (m, 7H), 7.41–7.36 (m, 2H), 7.26 (h, *J* = 7.7 Hz, 4H), 6.93 (dt, *J* = 16.6, 8.0 Hz, 1H), 6.73 (t, *J* = 8.5 Hz, 1H), 5.75 (s, 3H), 4.80 (d, *J* = 6.6 Hz, 6H), 3.25–3.12 (m, 1H), 2.81 (s, 2H), 1.88 (s, 1H), 1.63 (s, 4H), 1.33–1.19 (m, 2H). ^13^C NMR (100 MHz, MeO*D*) *δ* 158.6, 158.6, 157.9, 156.1, 156.1, 155.4, 141.2, 133.9, 131.8, 131.6, 130.4, 130.4, 130.4, 128.9, 128.9, 128.9, 128.7, 128.7, 128.6, 128.6, 125.6, 125. 6, 125.2, 125.2, 123.7, 123.7, 123.7, 123.3, 123.3, 123.2, 123.2, 122.5, 122.5, 122.4, 122.4, 109.0, 108.8, 57.6, 57.6, 57.5, 48.1, 47.9, 47.9, 47.7, 47.7, 47.5, 47.5, 47.2, 47.0. HRMS (ESI-) *m/z* calcd for C_39_H_39_N_6_O_6_S_2_ [M−H]^−^ 751.2373, found 751.2367. 

#### 3.11.2. N-((*S*)-2-(3,5-Difluorophenyl)-1-(6-(3-methyl-3-(methylsulfonyl)but-1-yn-1-yl)-3-(3-(methylsulfonamido)-1H-indazol-7-yl)pyridin-2-yl)ethyl)-2-(4,7-dimethyl-2-oxoindolin-3-yl)acetamide (**6b**)

Yield: 41%. ^1^H NMR (600 MHz, MeO*D*) *δ* 7.81 (d, *J* = 7.6 Hz, 0H), 7.65 (d, *J* = 7.8 Hz, 2H), 7.48 (d, *J* = 8.0 Hz, 1H), 7.39 (d, *J* = 7.2 Hz, 1H), 7.34 (s, 1H), 7.19 (d, *J* = 7.9 Hz, 2H), 6.83 (s, 1H), 6.23 (d, *J* = 13.0 Hz, 1H), 4.98 (d, *J* = 15.9 Hz, 1H), 4.79–4.68 (m, 1H), 4.58–4.46 (m, 1H), 3.58 (d, *J* = 13.8 Hz, 1H), 3.19 (s, 1H), 3.04 (s, 1H), 2.87 (s, 5H), 2.58 (s, 6H), 1.69 (s, 9H), 1.38 (s, 1H), 1.30 (t, *J* = 6.8 Hz, 2H). ^13^C NMR (100 MHz, MeO*D*) *δ* 184.3, 184.2, 175.5, 173.3, 172.7, 172.3, 170.0, 155.4, 144.7, 144.6, 138.2, 136.7, 136.3, 136.1, 134.8, 134.5, 134.3, 134.2, 134.2, 129.4, 129.3, 128.1, 125.2, 124.9, 120.7, 120.7, 96.8, 79.5, 79.4, 79.3, 64.1, 53.1, 52.9, 52.2, 52.0, 51.8, 51.6, 51.4, 51.2, 50.9, 49.5, 49.2, 46.2, 44.2, 43.8, 43.1, 43.1, 42.5, 41.5, 41.2, 39.0, 38.8, 38.5, 33.3, 33.3, 28.7, 27.2, 26.9, 26.8, 23.9, 23.8, 23.7, 23.6, 23. 5, 23.4, 23.2, 23.1, 23.1, 22.9, 21. 9, 20.1, 20.1, 20.0, 19.9, 19.8, 19.4, 18.9, 17.5, 17.1. HRMS (ESI-) *m/z* calcd for C_39_H_37_F_2_N_6_O_6_S_2_ [M−H]^−^ 787.2182, found 787.2178. 

#### 3.11.3. (*S*)-2-(5-Hydroxy-1H-indol-3-yl)-N-(1-(6-(3-methyl-3-(methylsulfonyl)but-1-yn-1-yl)-3-(3-(methylsulfonamido)-1H-indazol-7-yl)pyridin-2-yl)-2-phenylethyl)acetamide (**5a**)

Yield: 41%. ^1^H NMR (400 MHz, MeOD) δ 8.62 (d, J = 4.6 Hz, 1H), 8.32 (dd, J = 19.9, 11.1 Hz, 1H), 7.45 (d, J = 6.7 Hz, 4H), 7.36–7.30 (m, 1H), 7.27 (t, J = 5.4 Hz, 3H), 7.19–7.10 (m, 3H), 7.06 (d, J = 18.8 Hz, 1H), 7.03 (s, 1H), 6.95–6.86 (m, 1H), 6.74 (d, J = 8.7 Hz, 1H), 5.72 (d, J = 8.2 Hz, 1H), 4.78 (s, 6H), 4.11 (q, J = 7.3 Hz, 1H), 3.25 (s, 1H), 3.18 (s, 1H), 3.01 (s, 1H), 1.85–1.72 (m, 1H), 1.66 (s, 2H), 1.35–1.17 (m, 3H). ^13^C NMR (100 MHz, MeOD) δ 159.4, 159.0, 158.6, 157.7, 156.9, 156.47, 156.1, 155.29, 151.4, 151.3, 151.3, 139.7, 139.6, 135.2, 134.8, 132.4, 132.3, 132.1, 132.0, 131.9, 131.9, 131.6, 131.6, 130.8, 130.7, 130.4, 130.4, 130.4, 130.3, 130.2, 129.2, 129.1, 129.1, 128.9, 128.9, 128.9, 128.8, 128.7, 127.5, 127.4, 125.8, 125.6, 125.5, 125.3, 125.2, 125.1, 125.0, 124.2, 124.1, 124.1, 124.0, 123.9, 123.9, 123.8, 123.8, 123.7, 123.7, 123.7, 123.4, 123.3, 121.0, 108.8, 108.0, 77.9, 76.1, 76.0, 75.9, 69.3, 57.8, 38.8, 36.8, 34.2, 31. 7, 29.4, 23.7, 21.4. HRMS (ESI-) *m/z* calcd for C_37_H_35_N_6_O_6_S_2_ [M−H]^−^ 723.2060, found 723.2054. 

#### 3.11.4. (*S*)-N-(2-(3,5-Difluorophenyl)-1-(6-(3-methyl-3-(methylsulfonyl)but-1-yn-1-yl)-3-(3-(methylsulfonamido)-1H-indazol-7-yl)pyridin-2-yl)ethyl)-2-(5-hydroxy-1H-indol-3-yl)acetamide (**5b**)

Yield: 41%. ^1^H NMR (400 MHz, MeOD) δ 7.49 (t, J = 5.9 Hz, 4H), 7.21 (t, J = 7.7 Hz, 1H), 7.09 (d, J = 8.6 Hz, 1H), 6.93 (s, 1H), 6.87 (s, 1H), 6.72 (d, J = 12.8 Hz, 1H), 6.59 (s, 1H), 6.56 (d, J = 2.3 Hz, 1H), 6.28 (d, J = 7.4 Hz, 2H), 4.32 (d, J = 13.8 Hz, 1H), 4.16 (d, J = 14.0 Hz, 1H), 4.00 (q, J = 7.1 Hz, 1H), 3.21 (p, J = 1.6 Hz, 6H), 2.92 (d, J = 6.7 Hz, 1H), 1.71 (s, 7H), 1.14 (t, J = 7.1 Hz, 1H), 1.05 (s, 2H). ^13^C NMR (101 MHz, MeOD) δ 167.5, 164.2, 161.8, 158.6, 157.3, 155.6, 148.6, 141.4, 131.7, 130.4, 130.4, 128.9, 128.8, 128.7, 124.1, 123.7, 123.3, 116.5, 111.8, 101.8, 101.5, 90.5, 84.9, 78.2, 77. 9, 77. 6, 64.1, 57. 8, 57.5, 54.3, 34.1, 31.7, 29.4, 23.6, 21.7, 21.1. HRMS (ESI-) m/z calcd for C_37_H_33_F_2_N_6_O_6_S_2_ [M−H]^−^ 759.1869, found 759.1862.

### 3.12. Modeling and Docking Analysis

Molecular modeling was performed using the Schrödinger small molecule drug discovery suite 2021-3 (Schrödinger Inc., New York, NY, USA) [48]. We analyzed PF74-bound full length native HIV-1 CA (PDB ID: 4XFZ) by using Maestro (Schrödinger Inc.) [49]. Standard docking protocols were following protein preparation, grid generation, ligand preparation, and molecular docking. Protein preparation conducted by using the Protein preparation wizard (Schrödinger Inc.) [50] and involved the refinement of the protein structure. By using prime, the missing hydrogen atoms, side chains, and loops were refined into the protein. The OPLS3e force field was used to minimize the hydrogen bonding network and readjusting the heavy atoms to a rmsd of 0.3 Å [51]. The receptor grid generation tool in Maestro (Schrödinger Inc.) was utilized to standardize the binding site around the native ligand, surrounding all the key residues within the range of 12 Å. After sketching ligands in Maestro 2D Sketch tab, different conformers were generated in LigPrep [52] at pH of 7 ± 2 to serve as initial step for docking process. Finally, docking was conducted using the Glide XP (Glide, version 8.2, New York, NY, USA) [53] with a command as the van der Waals radii of nonpolar atoms for each of the ligands fixed by a factor of 0.8. After docking refinement and minimization, protein flexibility was also regarded under implicit solvent. All docked poses were subjected to analysis to cut off a small number of poses within the field of the receptor and binding pocket to generate better desired poses. Each docked pose was furtherance and presented in publication format using PyMOL (SchrodingerLLC) [54]. The numbering of residues of HIV-1 CA used in this paper for description was based on the full-length native HIV-1 CA.

### 3.13. Thermal Shift Assays (TSAs)

TSAs used purified covalently crosslinked hexameric CAA14C/E45C/W184A/M185A (CA121). CA121, cloned in a pET11a expression plasmid, was kindly provided by Dr. Owen Pornillos (University of Virginia, Charlottesville, VA, USA). CA121 was expressed in Escherichia coli BL21(DE3)RIL and purified according to reported protocols [55]. The TSAs were performed as previously described [56,57,58], with each reaction containing 7.5 µmol/L CA121 in 50 mmol/L sodium phosphate buffer (pH 8.0), 1× Sypro Orange Protein Gel Stain (Life Technologies, Carlsbad, CA, USA), and either 1% DMSO (control) or 20 µmol/L compound (1% DMSO final). The plate was heated from 25 to 95 °C with a heating rate of 0.2 °C every 10 s in the QuantStudio 3 Real-Time PCR system (Thermo Fisher Scientific, Waltham, MA, USA). The fluorescence intensity was measured with an Ex range of 475–500 nm and an Em range of 520–590 nm. The differences in the melting temperature (ΔTm) of CA121 in DMSO (T0) verses in the presence of compound (Tm) were calculated using the following Equation (1):

ΔTm (°C) = Tm − T0 (1)

### 3.14. Virus Production

The wild-type laboratory HIV-1 strain, HIV-1_NL4-3_ [59], was produced using a pNL4-3 vector (NIH AIDS Reagent Program, Division of AIDS, NIAID, NIH, Bethesda, MD, USA). HIV-1_NL4-3_ was generated by transfecting HEK 293FT cells with 10 µg of pNL4-3 vector and FuGENE^®^HD Transfection Reagent (Promega, Madison, WI, USA) in a T75 flask. The supernatant was harvested 48–72 h post-transfection and transferred to MT2 cells for viral propagation. The virus was harvested upon observation of syncytia formation, typically after 3–5 days. The viral supernatant was then concentrated using 8% *w*/*v* PEG 8000 overnight at 4 °C, followed by centrifugation for 40 min at 3500 rpm. The resulting viral-containing pellet was concentrated 10-fold by resuspension in DMEM without FBS and stored at −80 °C.

### 3.15. Anti-HIV-1 Assays

The anti-HIV-1 activity of compounds was examined in TZM-GFP cells. The potency of HIV-1 inhibition was determined based on the inhibition of viral LTR-activated GFP expression in the presence of compounds compared to DMSO controls. Briefly, TZM-GFP cells were plated at a density of 1 × 10^4^ cells per well in a 96-well plate. After 24 h, media was replaced with increasing concentrations of compound. Cells were exposed to HIV-1_NL4-3_ (MOI = 1) 24 h post treatment. After 48 h incubation, anti-HIV-1 activity was determined by counting the amount of GFP-positive cells on a Cytation^TM^ 5 Imaging Reader (BioTek, Winooski, VT, USA), and 50% effective concentration (EC_50_) values were determined. All cell-based assays were conducted in duplicate and in at least two independent experiments. Final values were calculated for each independent assay, and average values for all assays were calculated. Cells were observed periodically by microscope during the antiviral assays, and cell apoptosis was observed during the course of the assays, suggesting significant cytotoxicity effects from the compounds.

## 4. Conclusions

Based on the shared binding modes of **1**, PF74, and **2**, GS-6207, and necessitated by the need for novel sub-chemotypes of **2**, GS-6207, we have designed and synthesized molecular hybrids **5a**,**b** and **6a**,**b**, featuring the **1**, PF74, R^3^ moiety and R^1^ and R^4^ moieties of **2**, GS-6207. Per the induced-fit molecular docking, all four analogs bind to the CA-CA interface favorably. Synthetically, the newly designed analogs were constructed via a modular synthesis from the core component, C^2^, and the other three components, C^1^, C^3^, and C^4^, using highly reliable synthetic procedures. Although the current analogs only showed weak activities, the design and synthesis described herein contribute significantly to developing novel sub-chemotypes of **2**, GS-6207. The modular synthesis, in particular, can be adapted for synthesizing further designed analogs.

## Figures and Tables

**Table 1:** Thermal shift and cell-based antiviral analysis of selected compounds.

Compound	ΔT_m_ (°C) ^a^	EC_50_ (µM) ^b,c^
**5a**	0.18 ± 0.21	>11
**5b**	1.6 ± 0.19	>50
**6a**	0.12 ± 0.30	>33
**6b**	0.10 ± 0.24	>100
PF74	5.7 ± 0.48	0.55 ± 0.09

^a^ ΔT_m_: melting point change of CA hexamer in presence of compound compared to DMSO control. Mean ± standard deviation (SD) from at least two independent experiments. ^b^ Half maximal effective concentration from at least two independent experiments. ^c^ Cytotoxic effects from compounds were visible during EC_50_ determination; the results may be unreliable.

## Data Availability

The data produced from the current study are available from the corresponding author upon request. The original 1H and 13C NMR spectra for all synthetic compounds; the X-ray data of compound **22a**; HPLC method and traces for final compounds **5a**,**b** and **6a**,**b** (Figures S1–S36).

## Supplementary Materials

The following supporting information can be downloaded at https://www.mdpi.com/article/10.3390/ijms25073734/s1.

## Abbreviations

HIV	Human immunodeficiency virus
CA	Capsid protein
CPSF6	Cleavage and polyadenylation specific factor 6
CA_NTD_	CA N-terminal domain
CA_CTD_	CA C-terminal domain
SAR	Structure–activity relationship
HATU	1-[Bis(dimethylamino)methylene]-1*H*-1,2,3-triazolo [4,5-*b*]pyridinium3-oxid hexafluorophosphate
TSA	Thermal shift assay
EtOAc	Ethyl acetate
MeOH	Methanol
EtOH	Ethanol
DCM	Dichloromethane
DMF	*N, N*-Dimethylformamide
THF	Tetrahydrofuran
MeCN	Acetonitrile
IPA	Isopropyl alcohol
MnO_2_	Manganese dioxide
H_2_NOH.H_2_O	Hydroxylamine
Ac_2_O	Acetic anhydride
AcOH	Acetic acid
H_2_NNH_2_.H_2_O	Hydrazine hydrate
B_2_Pin_2_	4,4,5,5-tetramethyl-2-(tetramethyl-1,3,2-dioxaborolan-2-yl)-1,3,2-dioxaborolane
(Boc)_2_O	Di-*tert*-butyl pyrocarbonate
KOAc	Potassium acetate
NH_4_Cl	Ammonium chloride
NaOH	Sodium hydroxide
Cs_2_CO_3_	Cesium carbonate
K_2_CO_3_	Potassium carbonate
Na_2_SO_4_	Sodium sulfate
HCl	Hydrogen chloride
TFA	Trifluoroacetic acid
TEA	Triethylamine
DIPEA	*N, N*-Diisopropylethylamine
Pd(PPh_3_)_2_Cl_2_	Bis(triphenylphosphine)palladium(II)dichloride
Cu(I)Cl	Copper(I) chloride
Cu(I)I	Copper(I) iodide
TMPMgCl.LiCI	2,2,6,6-tetramethylpiperidinylmagnesium chloride, lithium chloride complex
Pd(dppf)_2_Cl_2_	[1,1′*bis*(diphenylphosphino)-ferrocene]dichloropalladium(II)
MeSO_2_Na	Sodium methanesulfonate
i-Pr_2_NEt	*N, N*-Diisopropylethylamine

## References

[1] Ding D., Xu S., Zhang X., Jiang X., Cocklin S., Dick A., Zhan P., Liu X. (2023). The discovery and design of novel HIV-1 capsid modulators and future perspectives. Expert Opin. Drug Discov..

[2] McFadden W.M., Snyder A.A., Kirby K.A., Tedbury P.R., Raj M., Wang Z., Sarafianos S.G. (2021). Rotten to the core: Antivirals targeting the HIV-1 capsid core. Retrovirology.

[3] Cevik M., Orkin C. (2019). Insights into HIV-1 capsid inhibitors in preclinical and early clinical development as antiretroviral agents. Expert Opin. Inv. Drug.

[4] Thenin-Houssier S., Valente S.T. (2016). HIV-1 Capsid Inhibitors as Antiretroviral Agents. Curr. HIV Res..

[5] Carnes S.K., Sheehan J.H., Aiken C. (2018). Inhibitors of the HIV-1 capsid, a target of opportunity. Curr. Opin. HIV AIDS.

[6] Wang L., Casey M.C., Vernekar S.K.V., Sahani R.L., Kankanala J., Kirby K.A., Du H., Hachiya A., Zhang H., Tedbury P.R. (2020). Novel HIV-1 capsid-targeting small molecules of the PF74 binding site. Eur. J. Med. Chem..

[7] Buffone C., Martinez-Lopez A., Fricke T., Opp S., Severgnini M., Cifola I., Petiti L., Frabetti S., Skorupka K., Zadrozny K.K. (2018). Nup153 Unlocks the Nuclear Pore Complex for HIV-1 Nuclear Translocation in Nondividing Cells. J. Virol..

[8] Matreyek K.A., Yucel S.S., Li X., Engelman A. (2013). Nucleoporin NUP153 phenylalanine-glycine motifs engage a common binding pocket within the HIV-1 capsid protein to mediate lentiviral infectivity. PLoS Pathog..

[9] Achuthan V., Perreira J.M., Sowd G.A., Puray-Chavez M., McDougall W.M., Paulucci-Holthauzen A., Wu X., Fadel H.J., Poeschla E.M., Multani A.S. (2018). Capsid-CPSF6 Interaction Licenses Nuclear HIV-1 Trafficking to Sites of Viral DNA Integration. Cell Host Microbe.

[10] Bejarano D.A., Peng K., Laketa V., Borner K., Jost K.L., Lucic B., Glass B., Lusic M., Mueller B., Krausslich H.G. (2019). HIV-1 nuclear import in macrophages is regulated by CPSF6-capsid interactions at the nuclear pore complex. Elife.

[11] Bhattacharya A., Alam S.L., Fricke T., Zadrozny K., Sedzicki J., Taylor A.B., Demeler B., Pornillos O., Ganser-Pornillos B.K., Diaz-Griffero F. (2014). Structural basis of HIV-1 capsid recognition by PF74 and CPSF6. Proc. Natl. Acad. Sci. USA.

[12] Rebensburg S.V., Wei G.C., Larue R.C., Lindenberger J., Francis A.C., Annamalai A.S., Morrison J., Shkriabai N., Huang S.W., KewalRamani V. (2021). Sec24C is an HIV-1 host dependency factor crucial for virus replication. Nat. Microbiol..

[13] Shi J., Zhou J., Shah V.B., Aiken C., Whitby K. (2011). Small-molecule inhibition of human immunodeficiency virus type 1 infection by virus capsid destabilization. J. Virol..

[14] Rankovic S., Ramalho R., Aiken C., Rousso I. (2018). PF74 Reinforces the HIV-1 Capsid To Impair Reverse Transcription-Induced Uncoating. J. Virol..

[15] Saito A., Ferhadian D., Sowd G.A., Serrao E., Shi J., Halambage U.D., Teng S., Soto J., Siddiqui M.A., Engelman A.N. (2016). Roles of Capsid-Interacting Host Factors in Multimodal Inhibition of HIV-1 by PF74. J. Virol..

[16] Link J.O., Rhee M.S., Tse W.C., Zheng J., Somoza J.R., Rowe W., Begley R., Chiu A., Mulato A., Hansen D. (2020). Clinical targeting of HIV capsid protein with a long-acting small molecule. Nature.

[17] Bester S.M., Wei G.C., Zhao H.Y., Adu-Ampratwum D., Iqbal N., Courouble V.V., Francis A.C., Annamalai A.S., Singh P.K., Shkriabai N. (2020). Structural and mechanistic bases for a potent HIV-1 capsid inhibitor. Science.

[18] Price A.J., Jacques D.A., McEwan W.A., Fletcher A.J., Essig S., Chin J.W., Halambage U.D., Aiken C., James L.C. (2014). Host cofactors and pharmacologic ligands share an essential interface in HIV-1 capsid that is lost upon disassembly. PLoS Pathog..

[19] Vernekar S.K.V., Sahani R.L., Casey M.C., Kankanala J., Wang L., Kirby K.A., Du H., Zhang H., Tedbury P.R., Xie J. (2020). Toward Structurally Novel and Metabolically Stable HIV-1 Capsid-Targeting Small Molecules. Viruses.

[20] Wang L., Casey M.C., Vernekar S.K.V., Do H.T., Sahani R.L., Kirby K.A., Du H., Hachiya A., Zhang H., Tedbury P.R. (2020). Chemical profiling of HIV-1 capsid-targeting antiviral PF74. Eur. J. Med. Chem..

[21] Wang L., Casey M.C., Vernekar S.K.V., Sahani R.L., Kirby K.A., Du H., Zhang H., Tedbury P.R., Xie J., Sarafianos S.G. (2021). Novel PF74-like small molecules targeting the HIV-1 capsid protein: Balance of potency and metabolic stability. Acta Pharm. Sin. B..

[22] Sahani R.L., Diana-Rivero R., Vernekar S.K.V., Wang L., Du H., Zhang H., Castaner A.E., Casey M.C., Kirby K.A., Tedbury P.R. (2021). Design, Synthesis and Characterization of HIV-1 CA-Targeting Small Molecules: Conformational Restriction of PF74. Viruses.

[23] Sahani R.L., Akther T., Cilento M.E., Castaner A.E., Zhang H., Kirby K.A., Xie J., Sarafianos S.G., Wang Z. (2021). Potency and metabolic stability: A molecular hybrid case in the design of novel PF74-like small molecules targeting HIV-1 capsid protein. RSC Med. Chem..

[24] Xu S.J., Sun L., Barnett M., Zhang X.J., Ding D., Gattu A., Shi D.Z., Taka J.R.H., Shen W.L., Jiang X.Y. (2023). Discovery, Crystallographic Studies, and Mechanistic Investigations of Novel Phenylalanine Derivatives Bearing a Quinazolin-4-one Scaffold as Potent HIV Capsid Modulators. J. Med. Chem..

[25] Jiang X.Y., Sharma P.P., Rathi B., Ji X.K., Hu L.D., Gao Z., Kang D.W., Wang Z., Xie M.H., Xu S.J. (2022). Discovery of novel 1,2,4-triazole phenylalanine derivatives targeting an unexplored region within the interprotomer pocket of the HIV capsid protein. J. Med. Virol..

[26] Ji X.K., Li J., Sharma P.P., Jiang X.Y., Rathi B., Gao Z., Hu L.D., Kang D.W., De Clercq E., Cocklin S. (2022). Design, Synthesis and Structure-Activity Relationships of Phenylalanine-Containing Peptidomimetics as Novel HIV-1 Capsid Binders Based on Ugi Four-Component Reaction. Molecules.

[27] Jiang X.Y., Wu G.C., Zalloum W.A., Meuser M.E., Dick A., Sun L., Chen C.H., Kang D.W., Jing L.L., Jia R.F. (2019). Discovery of novel 1,4-disubstituted 1,2,3-triazole phenylalanine derivatives as HIV-1 capsid inhibitors. RSC Adv..

[28] Sun L., Dick A., Meuser M.E., Huang T.G., Zalloum W.A., Chen C.H., Cherukupalli S., Xu S.J., Ding X., Gao P. (2020). Design, Synthesis, and Mechanism Study of Benzenesulfonamide-Containing Phenylalanine Derivatives as Novel HIV-1 Capsid Inhibitors with Improved Antiviral Activities. J. Med. Chem..

[29] Li J., Jiang X.Y., Dick A., Sharma P.P., Chen C.H., Rathi B., Kang D.W., Wang Z., Ji X.K., Lee K.H. (2021). Design, synthesis, and antiviral activity of phenylalanine derivatives as HIV-1 capsid inhibitors. Bioorg. Med. Chem..

[30] Sun L., Huang T.G., Dick A., Meuser M.E., Zalloum W.A., Chen C.H., Ding X., Gao P., Cocklin S., Lee K.H. (2020). Design, synthesis and structure-activity relationships of 4-phenyl-1-1,2,3-triazole phenylalanine derivatives as novel HIV-1 capsid inhibitors with promising antiviral activities. Eur. J. Med. Chem..

[31] Wu G.C., Zalloum W.A., Meuser M.E., Jing L.L., Kang D.W., Chen C.H., Tian Y., Zhang F.F., Cocklin S., Lee K.H. (2018). Discovery of phenylalanine derivatives as potent HIV-1 capsid inhibitors from click chemistry-based compound library. Eur. J. Med. Chem..

[32] Xu S.J., Sun L., Dick A., Zalloum W.A., Huang T.G., Meuser M.E., Zhang X.J., Tao Y.C., Cherukupalli S., Ding D. (2022). Design, synthesis, and mechanistic investigations of phenylalanine derivatives containing a benzothiazole moiety as HIV-1 capsid inhibitors with improved metabolic stability. Eur. J. Med. Chem..

[33] Mushtaq A., Kazi F. (2023). Lenacapavir: A new treatment of resistant HIV-1 infections. Lancet Infect. Dis..

[34] Margot N., Vanderveen L., Naik V., Ram R., Parvangada P., Martin R., Rhee M., Callebaut C. (2022). Phenotypic resistance to lenacapavir and monotherapy efficacy in a proof-of-concept clinical study. J. Antimicrob. Chemother..

[35] Margot N.A., Naik V., VanderVeen L., Anoshchenko O., Singh R., Dvory-Sobol H., Rhee M.S., Callebaut C. (2022). Resistance Analyses in Highly Treatment-Experienced People With Human Immunodeficiency Virus (HIV) Treated with the Novel Capsid HIV Inhibitor Lenacapavir. J. Infect. Dis..

[36] Marcelin A.-G., Charpentier C., Jary A., Perrier M., Margot N., Callebaut C., Calvez V., Descamps D. (2020). Frequency of capsid substitutions associated with GS-6207 in vitro resistance in HIV-1 from antiretroviral-naive and -experienced patients. J. Antimicrob. Chemother..

[37] Gres A.T., Kirby K.A., KewalRamani V.N., Tanner J.J., Pornillos O., Sarafianos S.G. (2015). X-ray crystal structures of native HIV-1 capsid protein reveal conformational variability. Science.

[38] Gritter R.J., Dupre G.D., Wallace T.J. (1964). Oxidation of Benzyl Alcohols with Manganese Dioxide. Nature.

[39] Mowry D.T. (1948). The Preparation of Nitriles. Chem. Rev..

[40] Rohrbach S., Smith A.J., Pang J.H., Poole D.L., Tuttle T., Chiba S., Murphy J.A. (2019). Concerted Nucleophilic Aromatic Substitution Reactions. Angew. Chem. Int. Ed..

[41] Billingsley K.L., Buchwald S.L. (2008). An Improved System for the Palladium-Catalyzed Borylation of Aryl Halides with Pinacol Borane. J. Org. Chem..

[42] Schlecker W., Huth A., Ottow E., Mulzer J. (1995). Regioselective Metalation of Pyridinylcarbamates and Pyridinecarboxamides with (2,2,6,6-Tetramethylpiperidino)magnesium Chloride. J. Org. Chem..

[43] Ellman J.A., Owens T.D., Tang T.P. (2002). N-tert-Butanesulfinyl Imines:  Versatile Intermediates for the Asymmetric Synthesis of Amines. Acc. Chem. Res..

[44] Reddy R.J., Kumari A.H. (2021). Synthesis and applications of sodium sulfinates (RSO(2)Na): A powerful building block for the synthesis of organosulfur compounds. RSC Adv..

[45] Sonogashira K. (2002). Development of Pd–Cu catalyzed cross-coupling of terminal acetylenes with sp2-carbon halides. J. Organomet. Chem..

[46] Miyaura N., Yamada K., Suzuki A. (1979). A new stereospecific cross-coupling by the palladium-catalyzed reaction of 1-alkenylboranes with 1-alkenyl or 1-alkynyl halides. Tetrahedron Lett..

[47] Carpino L.A. (1993). 1-Hydroxy-7-azabenzotriazole. An efficient peptide coupling additive. J. Am. Chem. Soc..

[48] Schrödinger (2021). Small-Molecule Drug Discovery Suite 2021-3.

[49] Schrödinger (2021). Release 2021-3: Maestro.

[50] Sastry G.M., Adzhigirey M., Day T., Annabhimoju R., Sherman W. (2013). Protein and ligand preparation: Parameters, protocols, and influence on virtual screening enrichments. J. Comput. Aided Mol. Des..

[51] Jorgensen W.L., Maxwell D.S., TiradoRives J. (1996). Development and testing of the OPLS all-atom force field on conformational energetics and properties of organic liquids. J. Am. Chem. Soc..

[52] Schrödinger (2021). Release 2021-3: LigPrep.

[53] Friesner R.A., Banks J.L., Murphy R.B., Halgren T.A., Klicic J.J., Mainz D.T., Repasky M.P., Knoll E.H., Shelley M., Perry J.K. (2004). Glide: A new approach for rapid, accurate docking and scoring. 1. Method and assessment of docking accuracy. J. Med. Chem..

[54] Schrödinger (2021). The PyMOL Molecular Graphics System, Version 2.0.

[55] Pornillos O., Ganser-Pornillos B.K., Kelly B.N., Hua Y., Whitby F.G., Stout C.D., Sundquist W.I., Hill C.P., Yeager M. (2009). X-ray structures of the hexameric building block of the HIV capsid. Cell.

[56] Lo M.C., Aulabaugh A., Jin G., Cowling R., Bard J., Malamas M., Ellestad G. (2004). Evaluation of fluorescence-based thermal shift assays for hit identification in drug discovery. Anal. Biochem..

[57] Miyazaki Y., Doi N., Koma T., Adachi A., Nomaguchi M. (2017). Novel In Vitro Screening System Based on Differential Scanning Fluorimetry to Search for Small Molecules against the Disassembly or Assembly of HIV-1 Capsid Protein. Front. Microbiol..

[58] Pantoliano M.W., Petrella E.C., Kwasnoski J.D., Lobanov V.S., Myslik J., Graf E., Carver T., Asel E., Springer B.A., Lane P. (2001). High-density miniaturized thermal shift assays as a general strategy for drug discovery. J. Biomol. Screen.

[59] Adachi A., Gendelman H.E., Koenig S., Folks T., Willey R., Rabson A., Martin M.A. (1986). Production of acquired immunodeficiency syndrome-associated retrovirus in human and nonhuman cells transfected with an infectious molecular clone. J. Virol..

