# Supplementary material for: Design and Synthesis of New GS-6207 Subtypes for Targeting HIV-1 Capsid Protein
Source: Int J Mol Sci. 2024 Mar 27;25(7):3734. doi: 10.3390/ijms25073734 (PMC11012105; doi:10.3390/ijms25073734)
Supplement: Supplementary file 1 [file ijms-25-03734-s001.zip › ijms-2884618-supplementary.pdf]

Supporting Information

Design and synthesis of new GS-6207 subtypes for targeting HIV-1 capsid protein

Thamina Akther[1], William M. McFadden[2], Huanchun Zhang[2], Karen A. Kirby[2], Stefan G. Sarafianos[2], Zhengqiang Wang[1]*

[1] Center for Drug Design, College of Pharmacy, University of Minnesota, Minneapolis, MN 55455, USA

[2] Laboratory of Biochemical Pharmacology, Department of Pediatrics, Emory University School of Medicine, Atlanta, GA 30322, USA

*Corresponding author

E-mail address: wangx472@umn.edu. (Z. Wang)

Tel.: +1 612 626 7025 (Z. Wang)

Figure S1: ^{1}H- and ^{13}C-NMR spectra of compound (**12**).

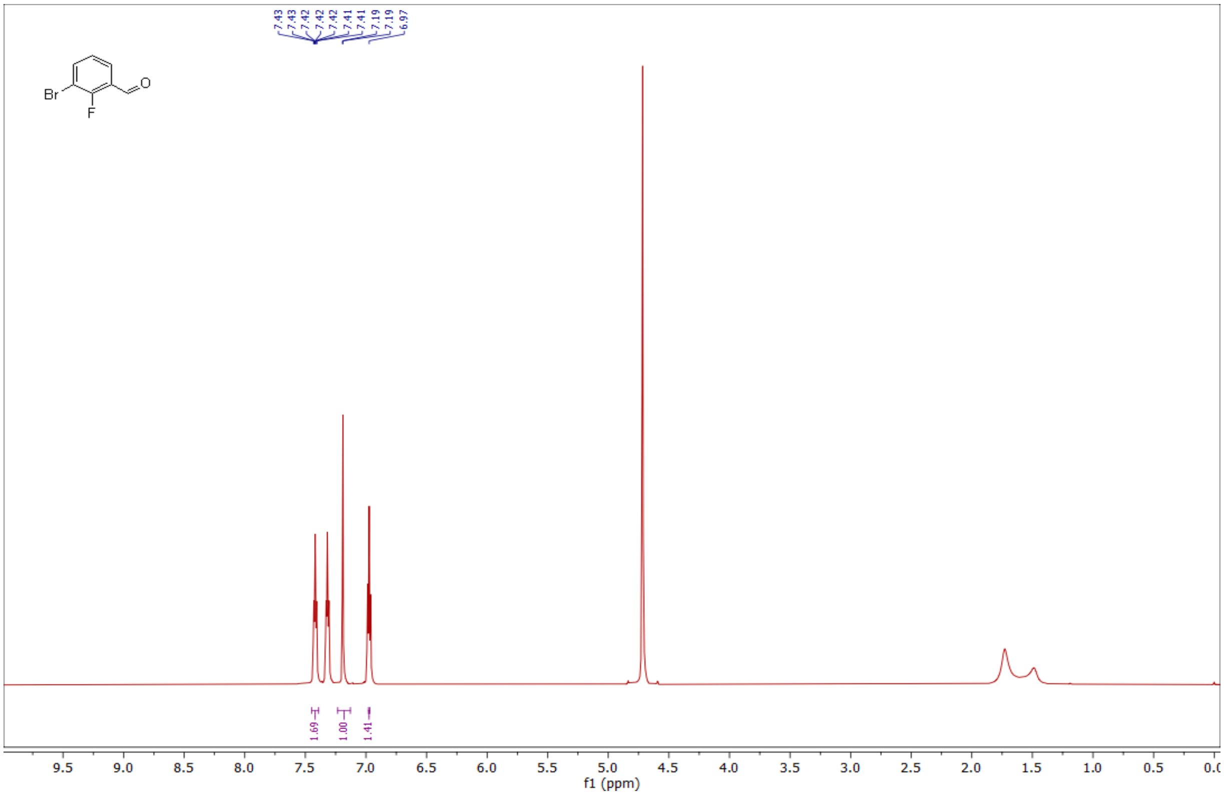

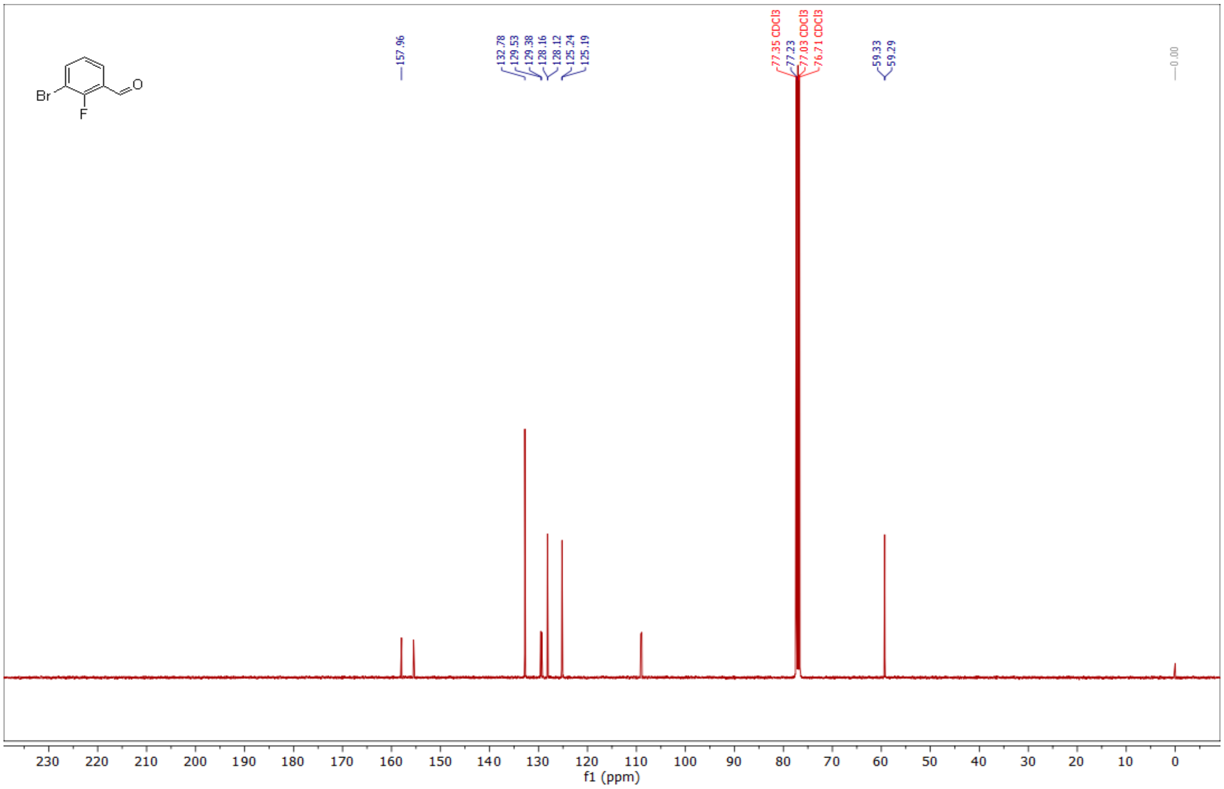

Figure S2: ^{1}H- and ^{13}C-NMR spectra of compound (**13**).

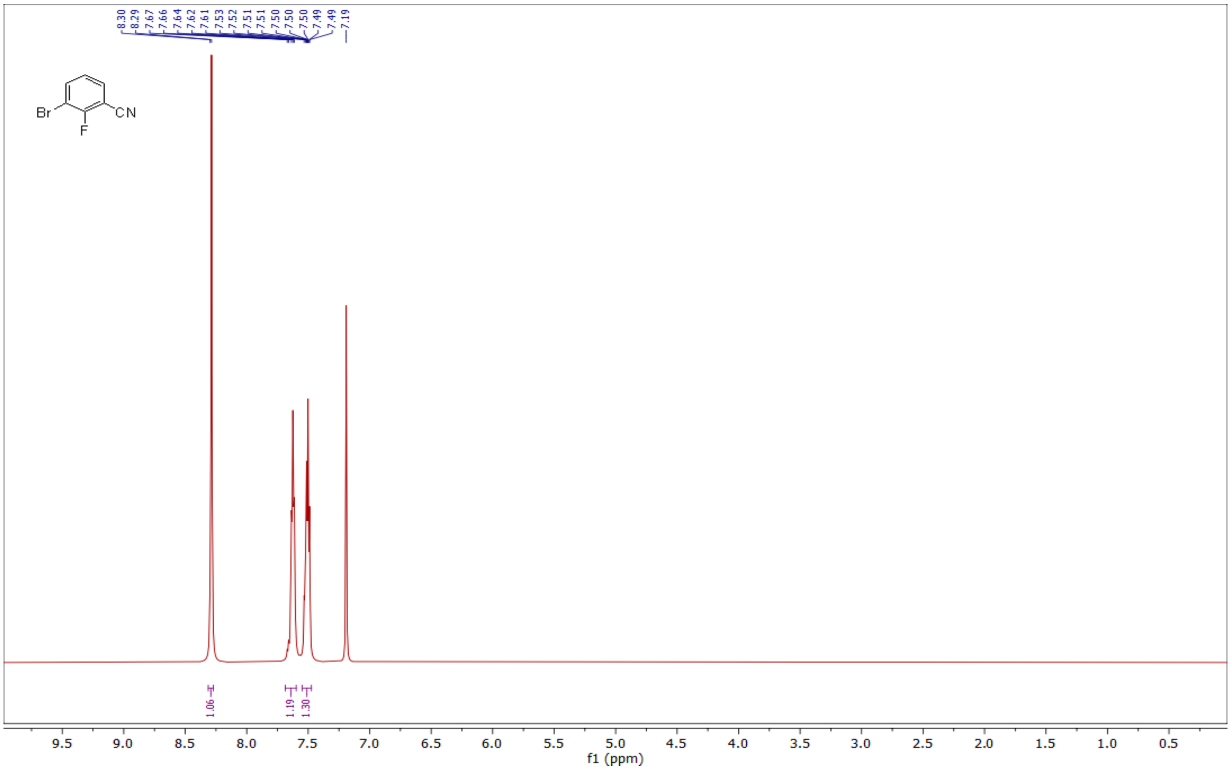

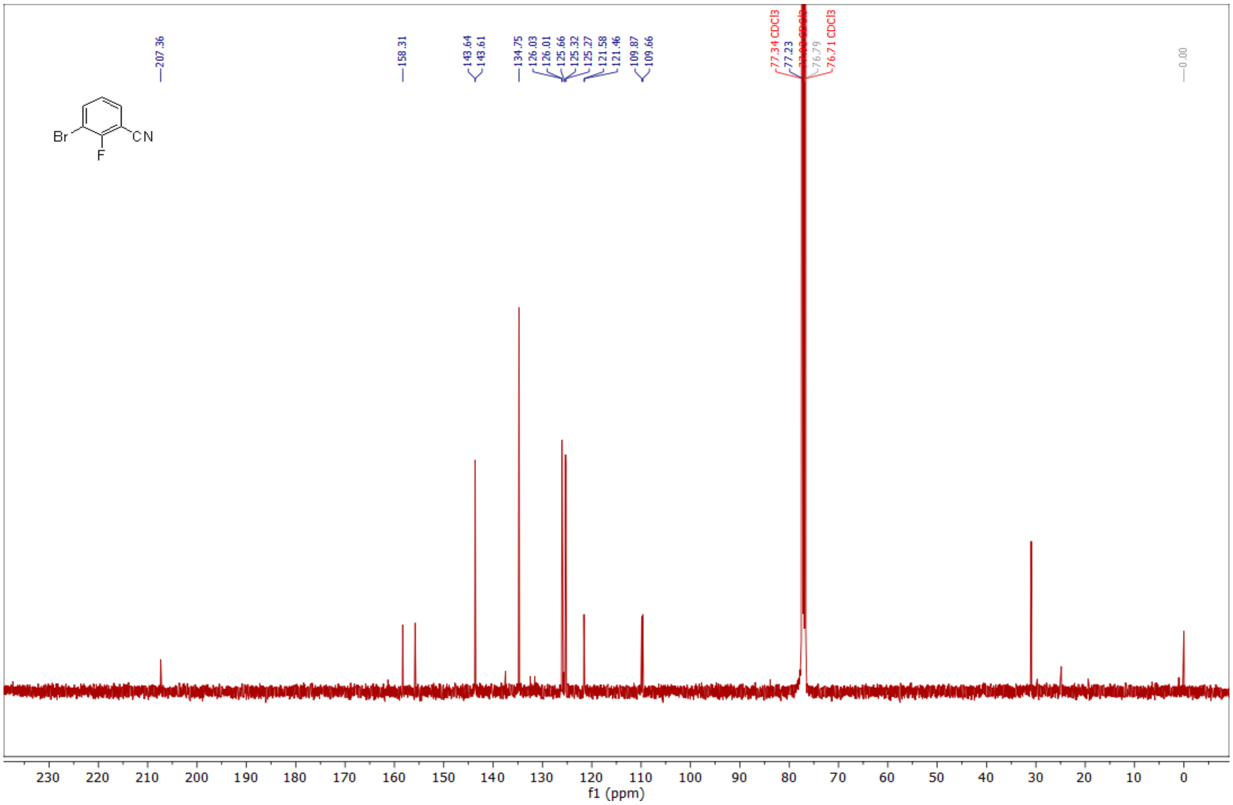

Figure S3: ^{1}H- and ^{13}C-NMR spectra of compound (**14**).

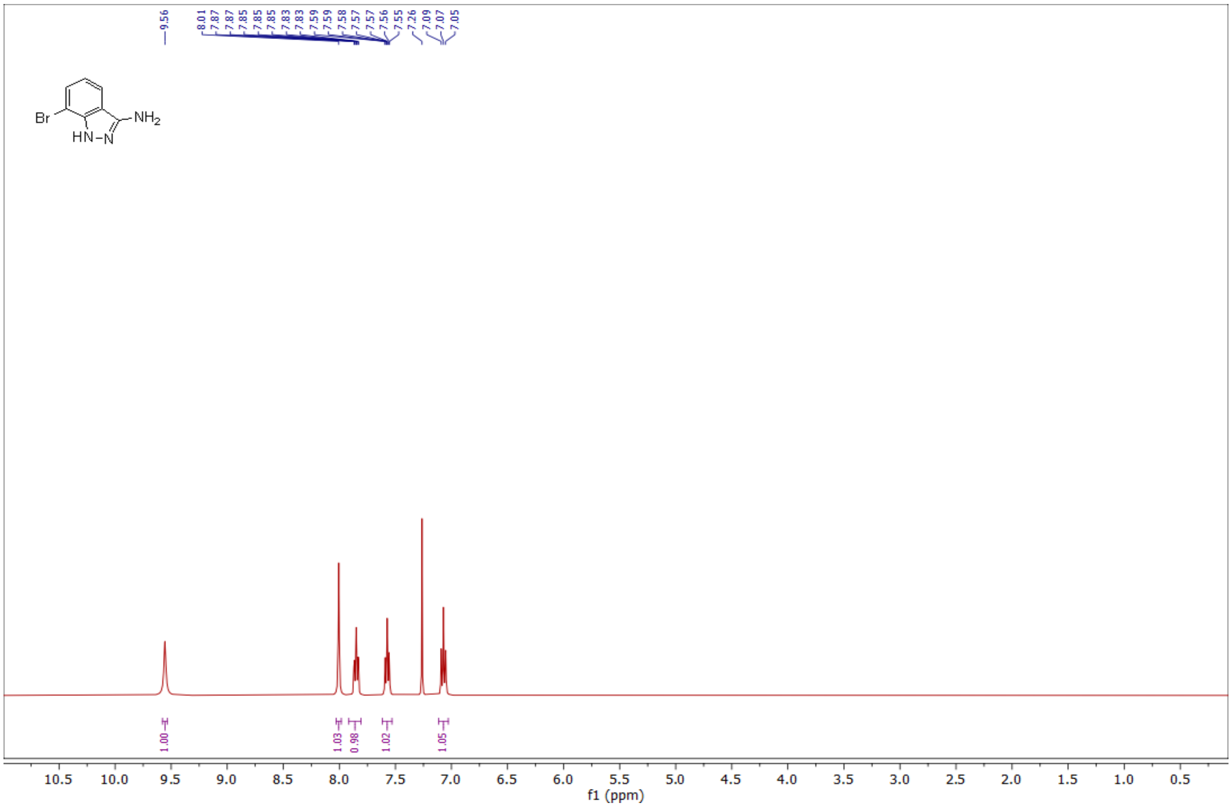

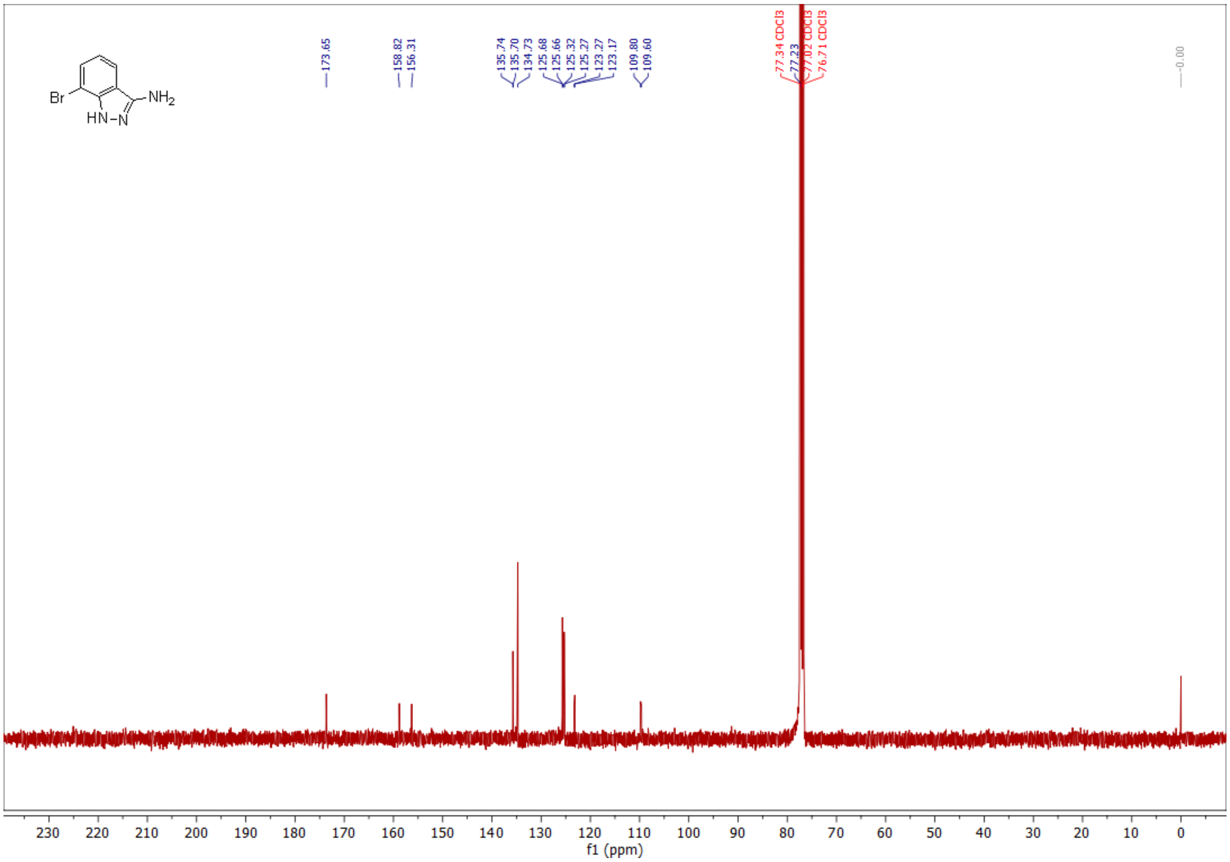

Figure S4: ^{1}H- and ^{13}C-NMR spectra of compound (**7**).

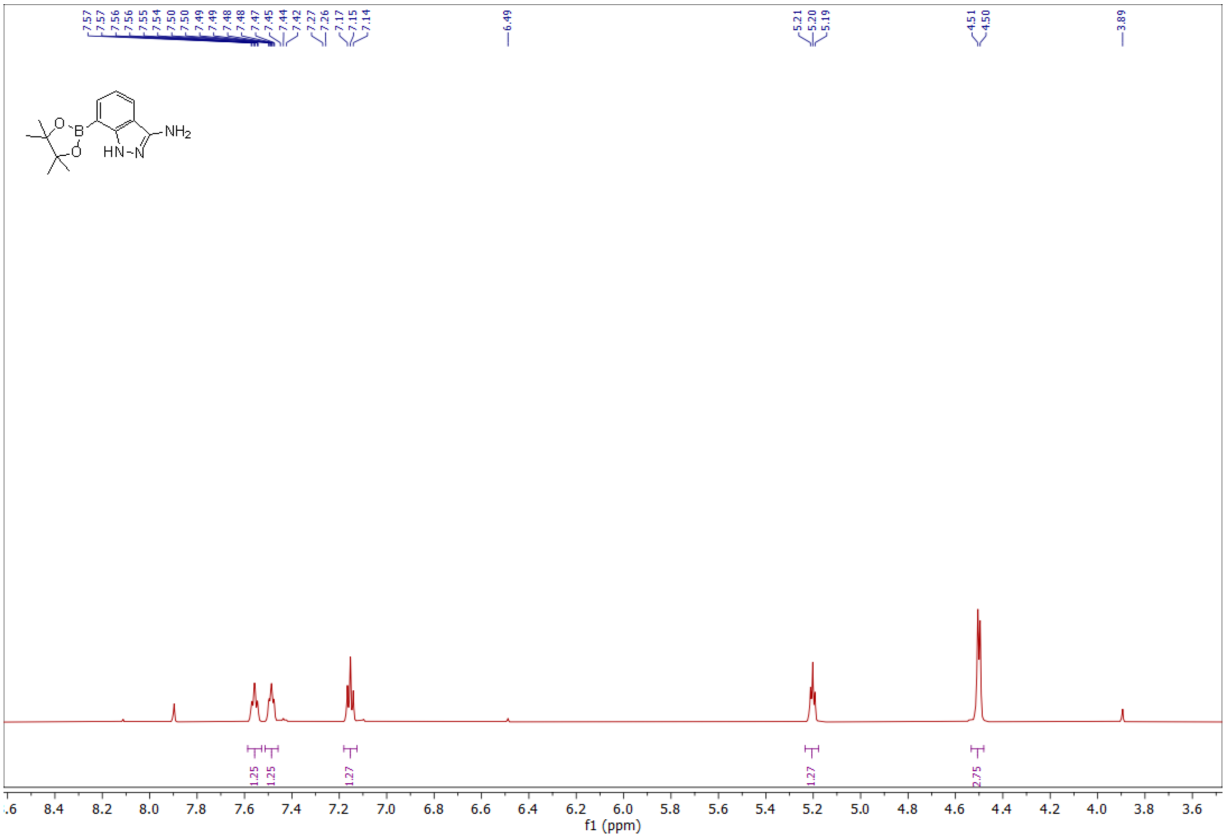

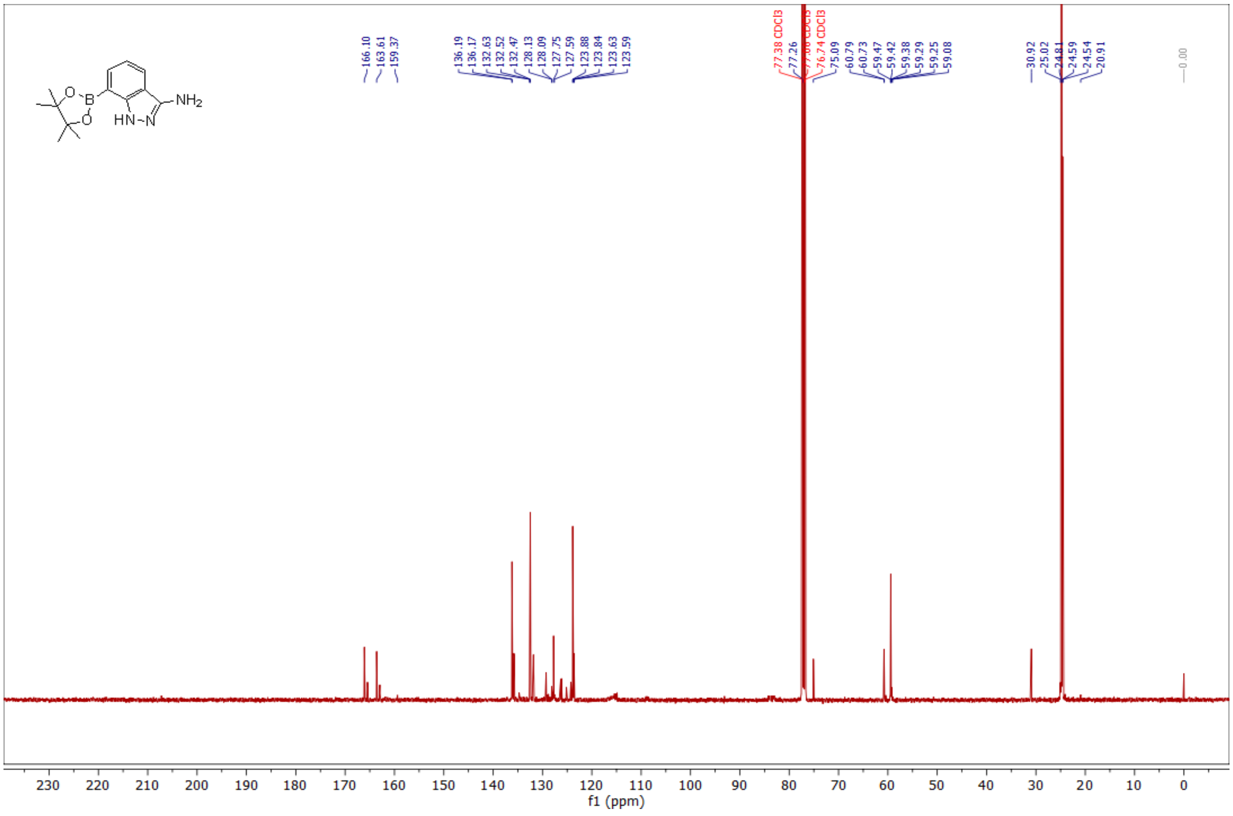

Figure S5: ^{1}H- and ^{13}C-NMR spectra of compound (**16**).

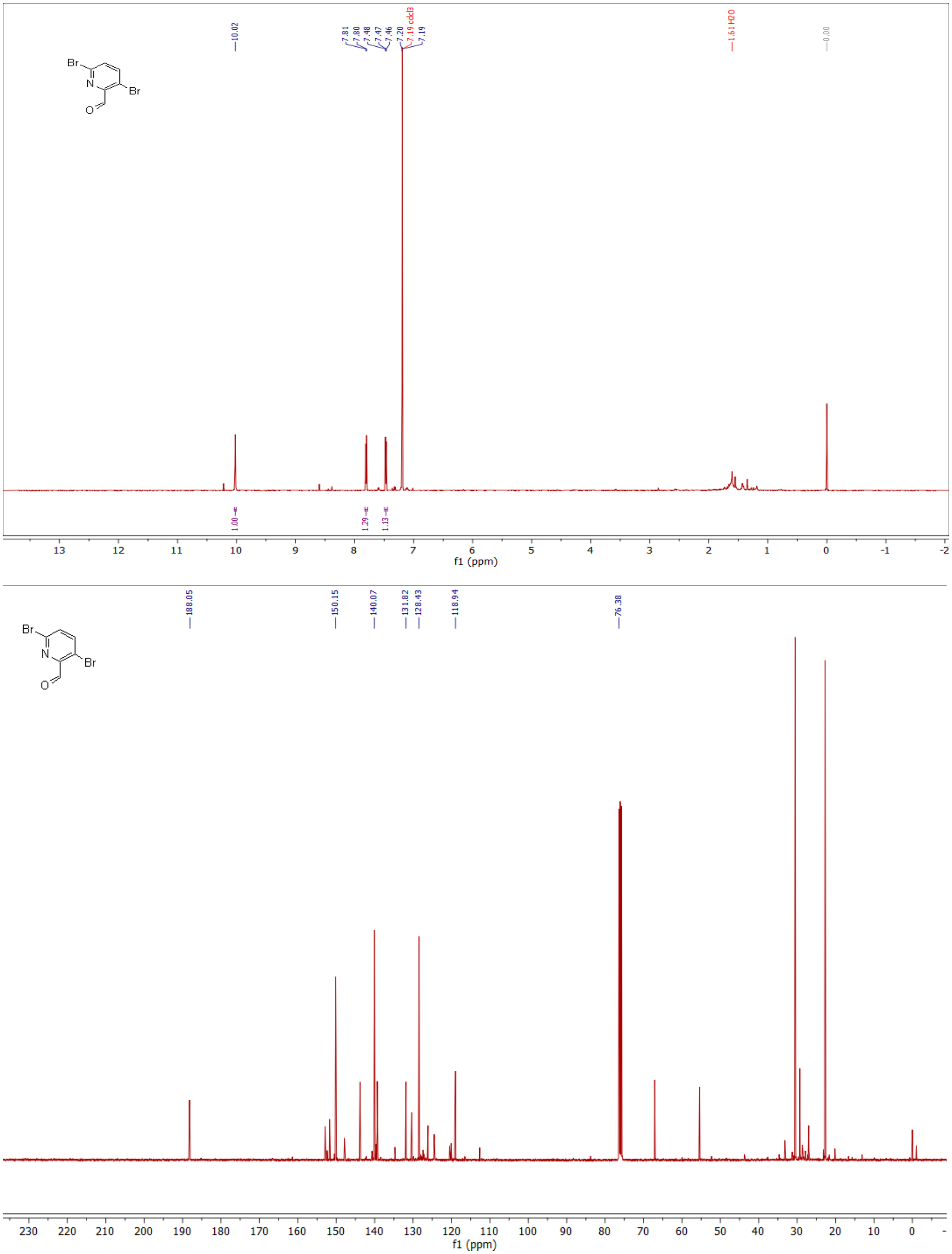

Figure S6: ^{1}H- and ^{13}C-NMR spectra of compound (**17**).

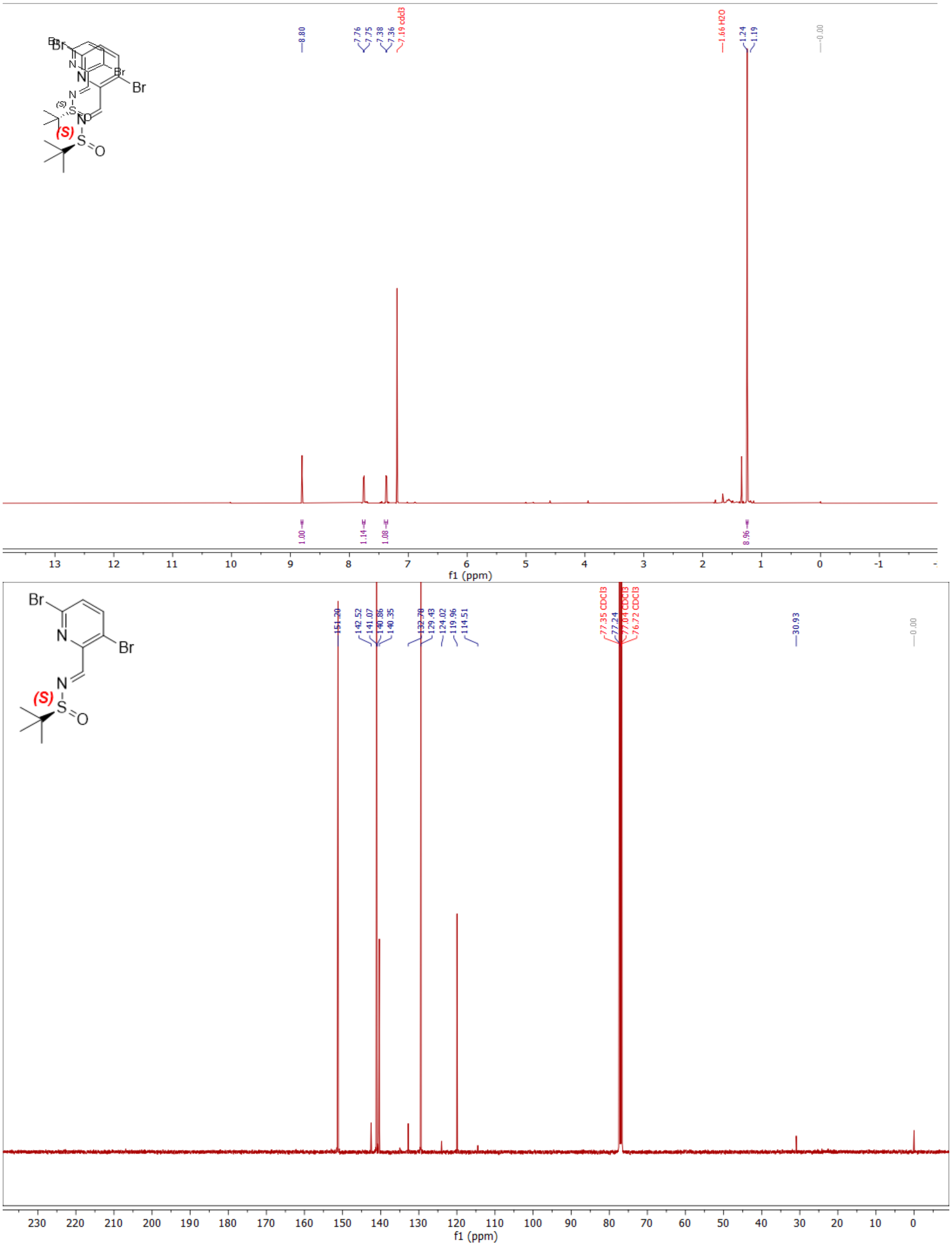

Figure S7: ^{1}H- and ^{13}C-NMR spectra of compound (**18**).

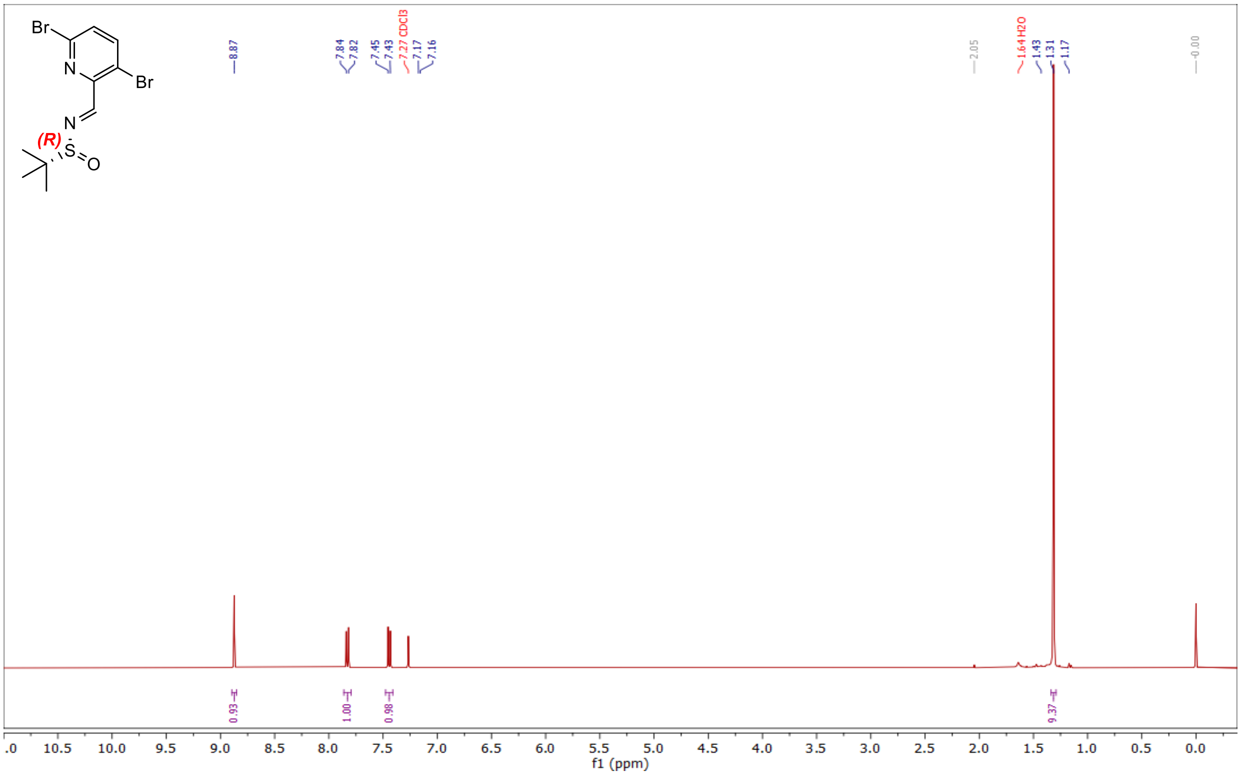

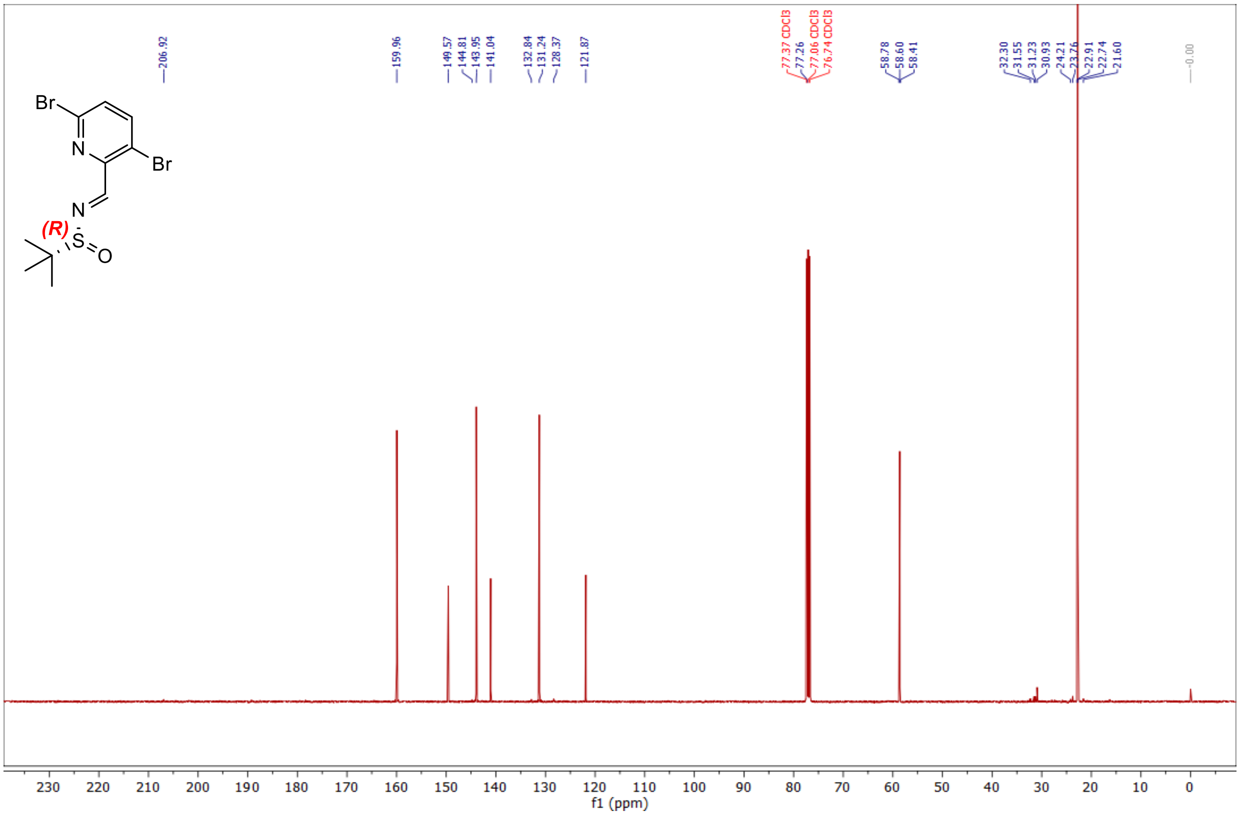

Figure S8: ^{1}H- and ^{13}C-NMR spectra of compound (**19a**).

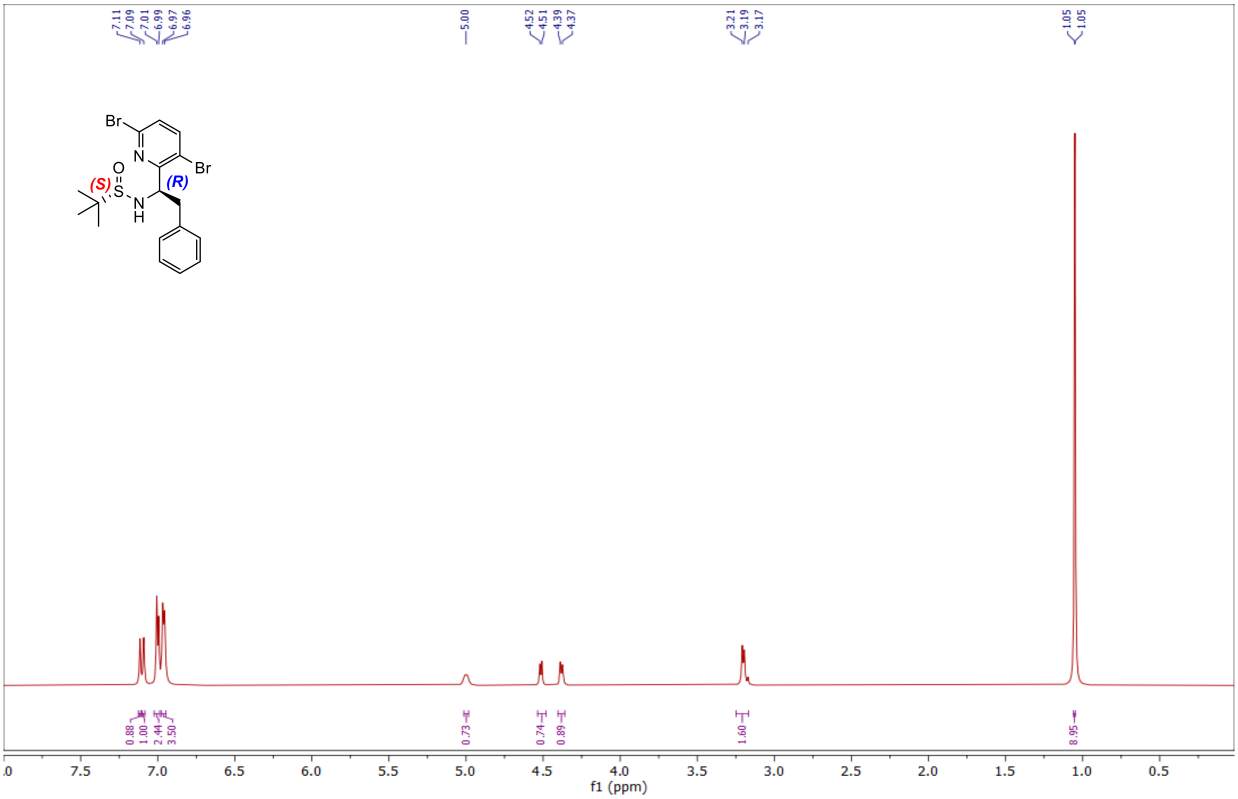

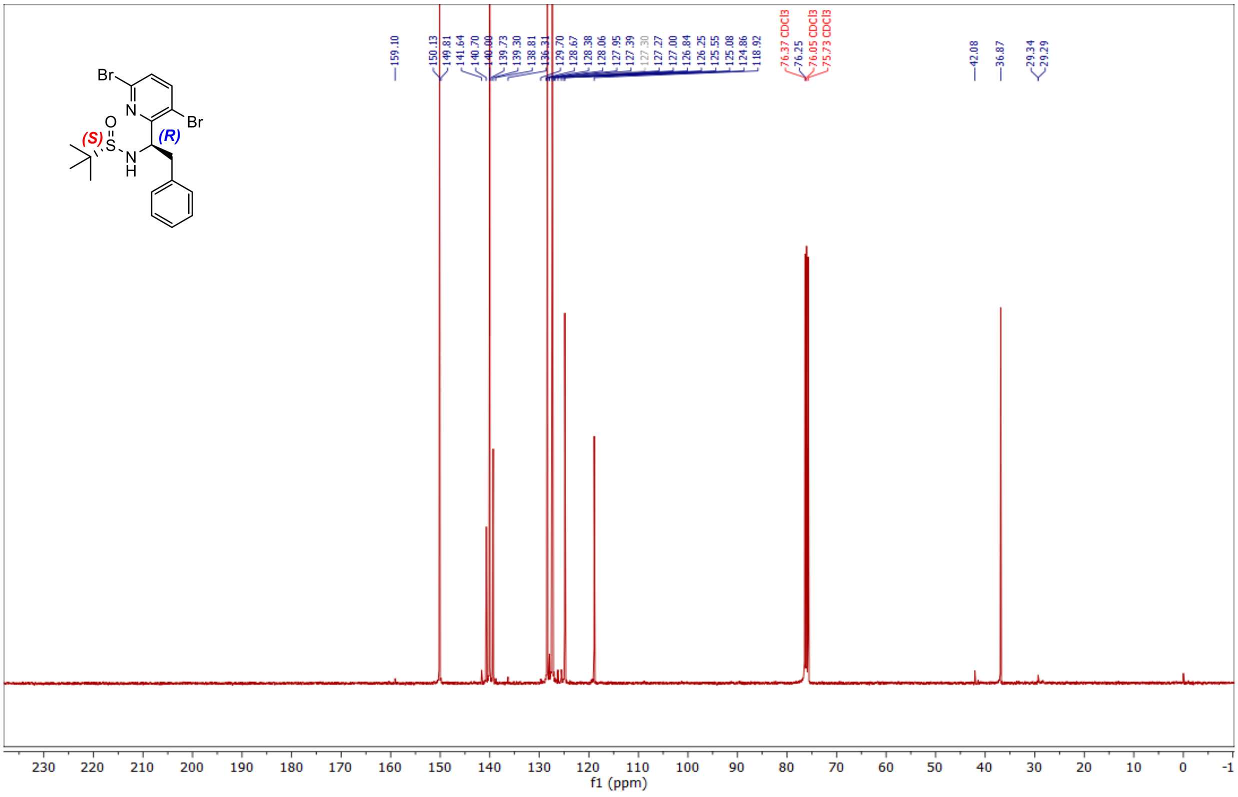

Figure S9: ^{1}H- and ^{13}C-NMR spectra of compound (**19b**).

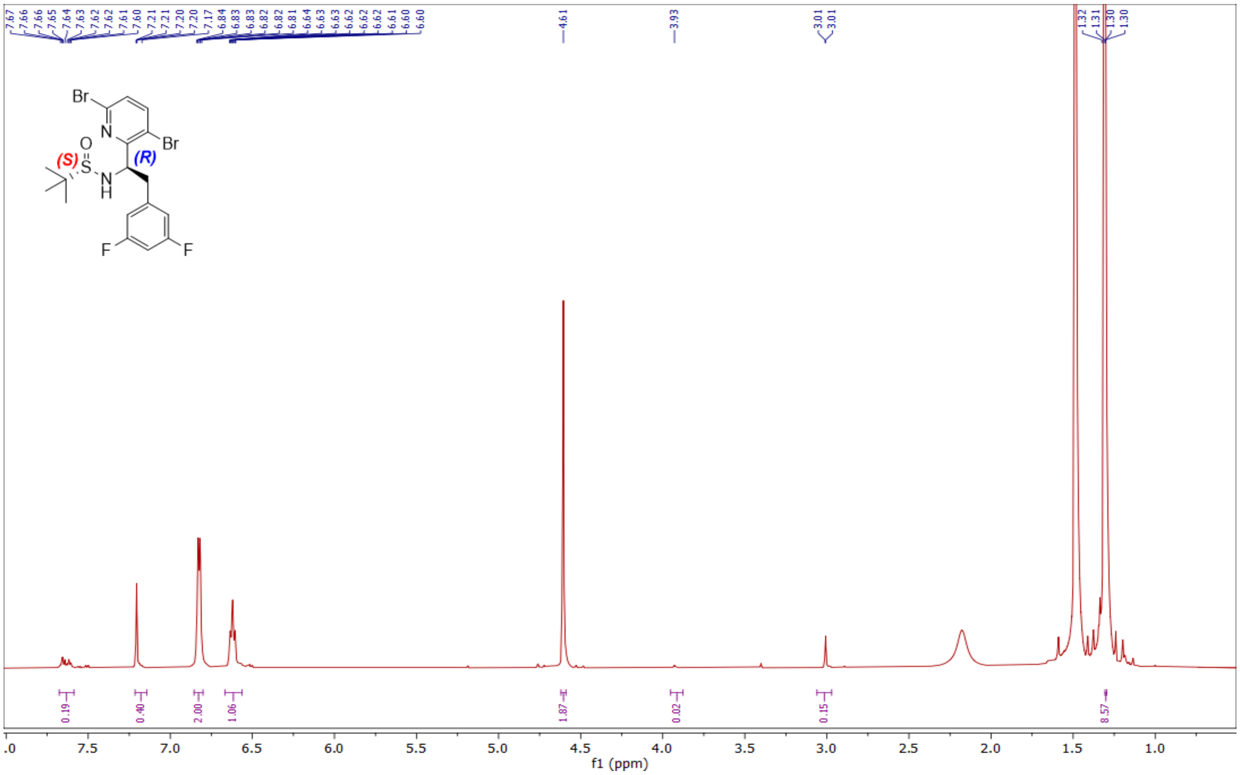

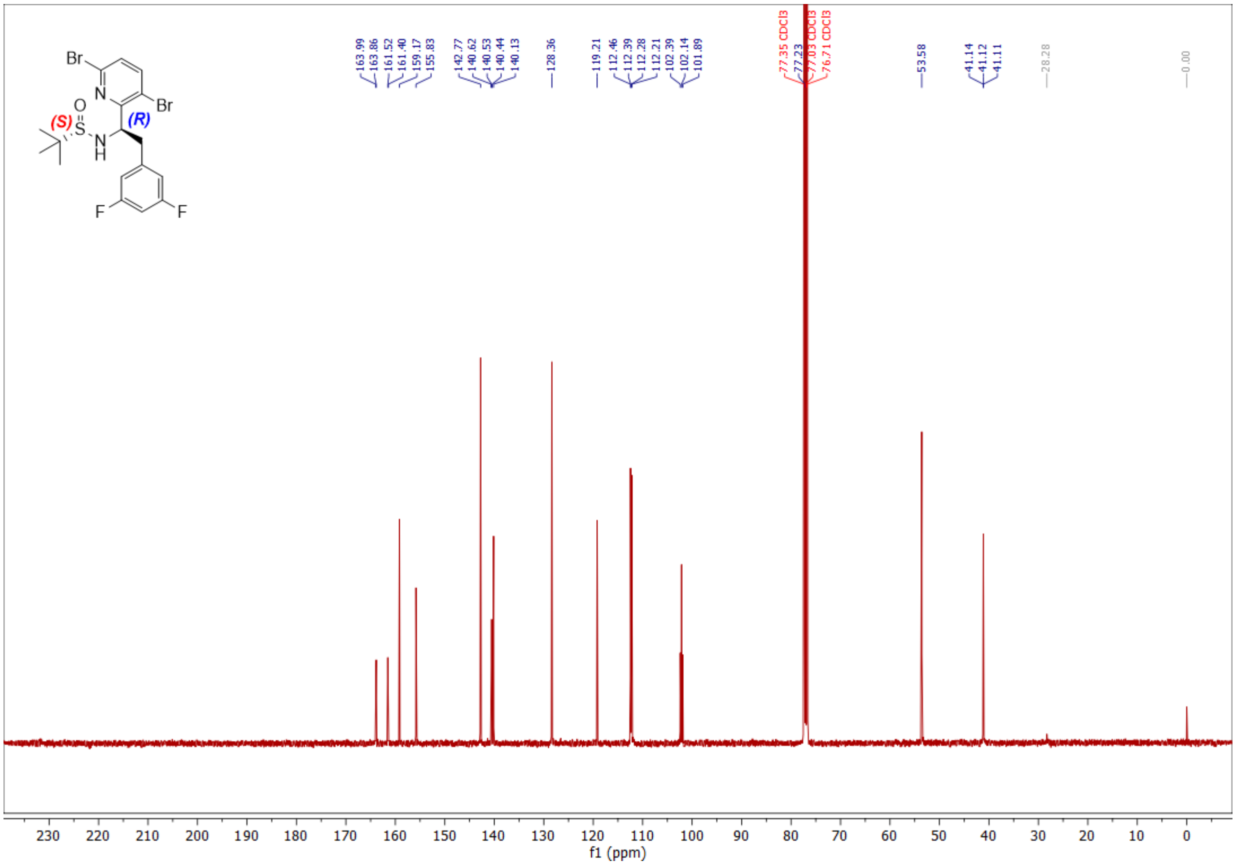

Figure S10: ^{1}H-NMR spectra of compound (**20a**).

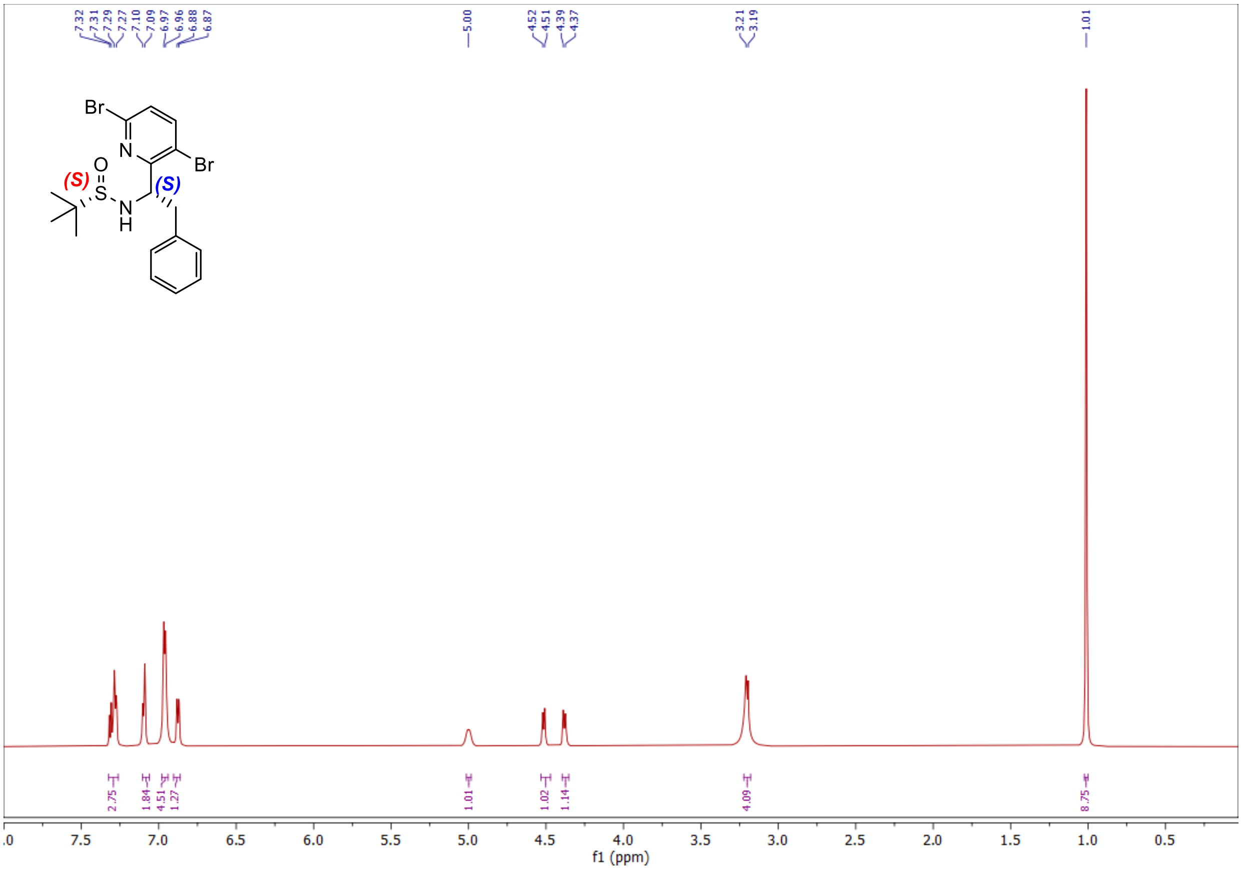

Figure S11: ^{1}H- and ^{13}C-NMR spectra of compound (**21a**).

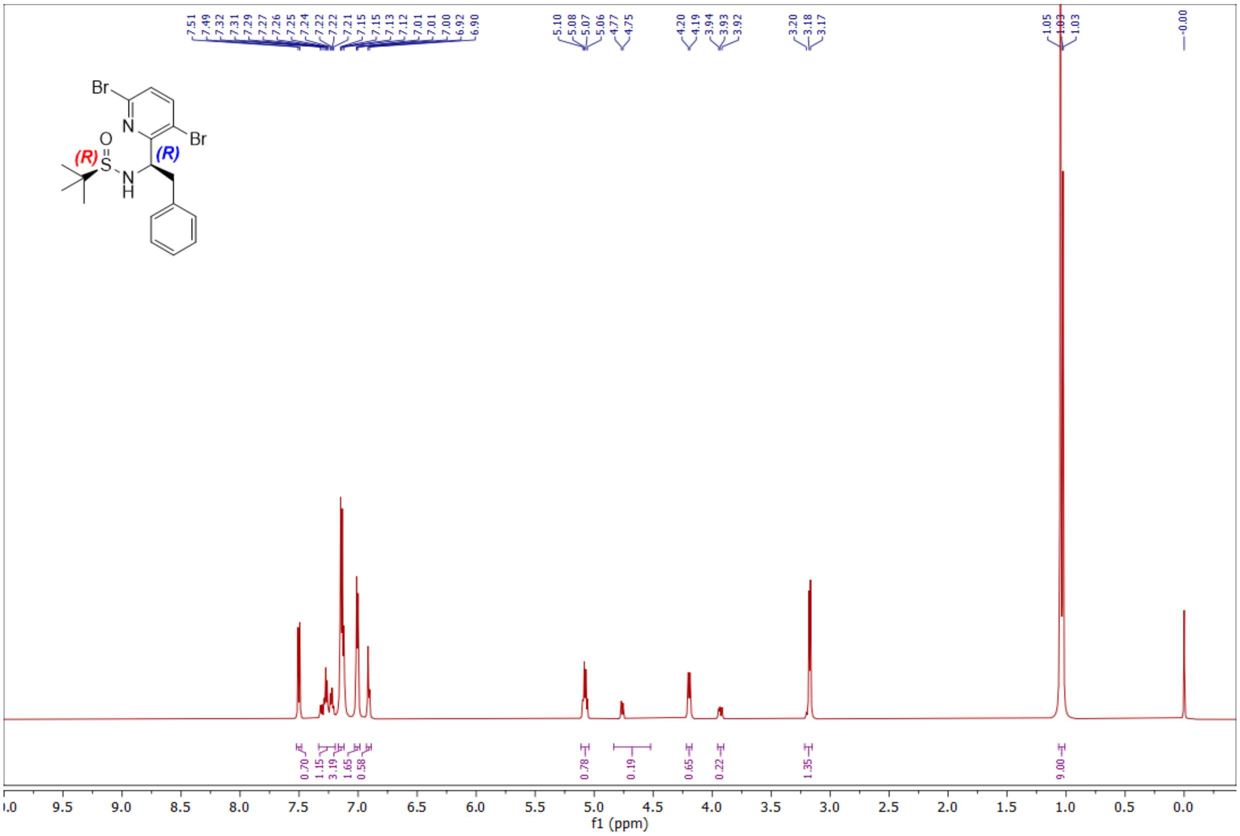

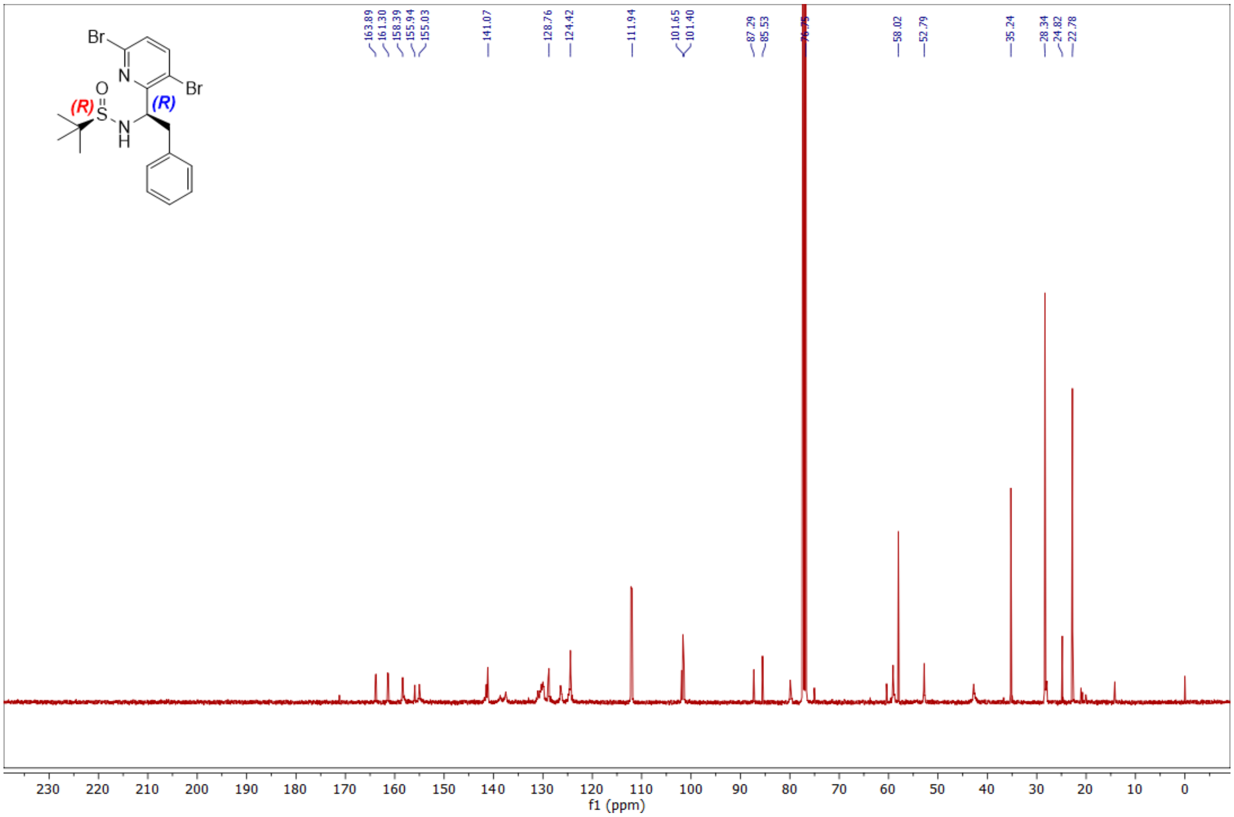

Figure S12: ^{1}H-NMR spectra of compound (**21b**).

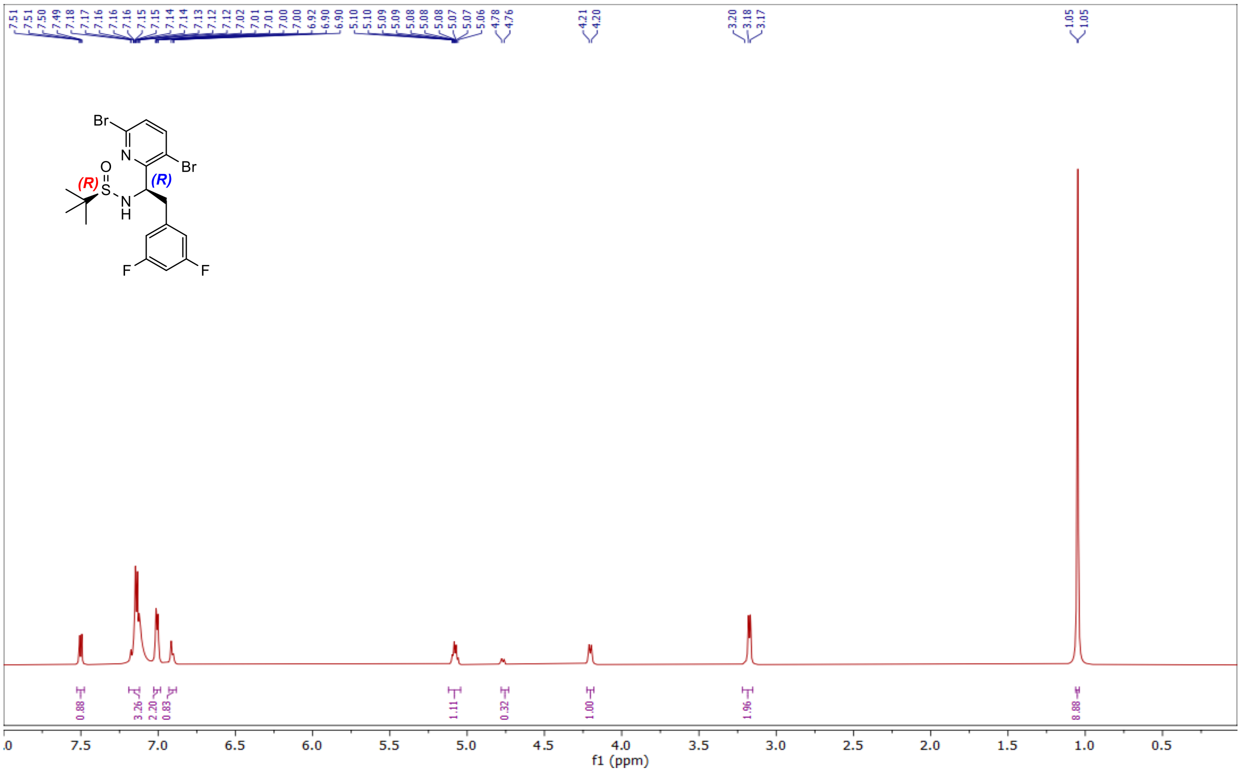

Figure S13: ^{1}H-NMR spectra of compound (**22a**).

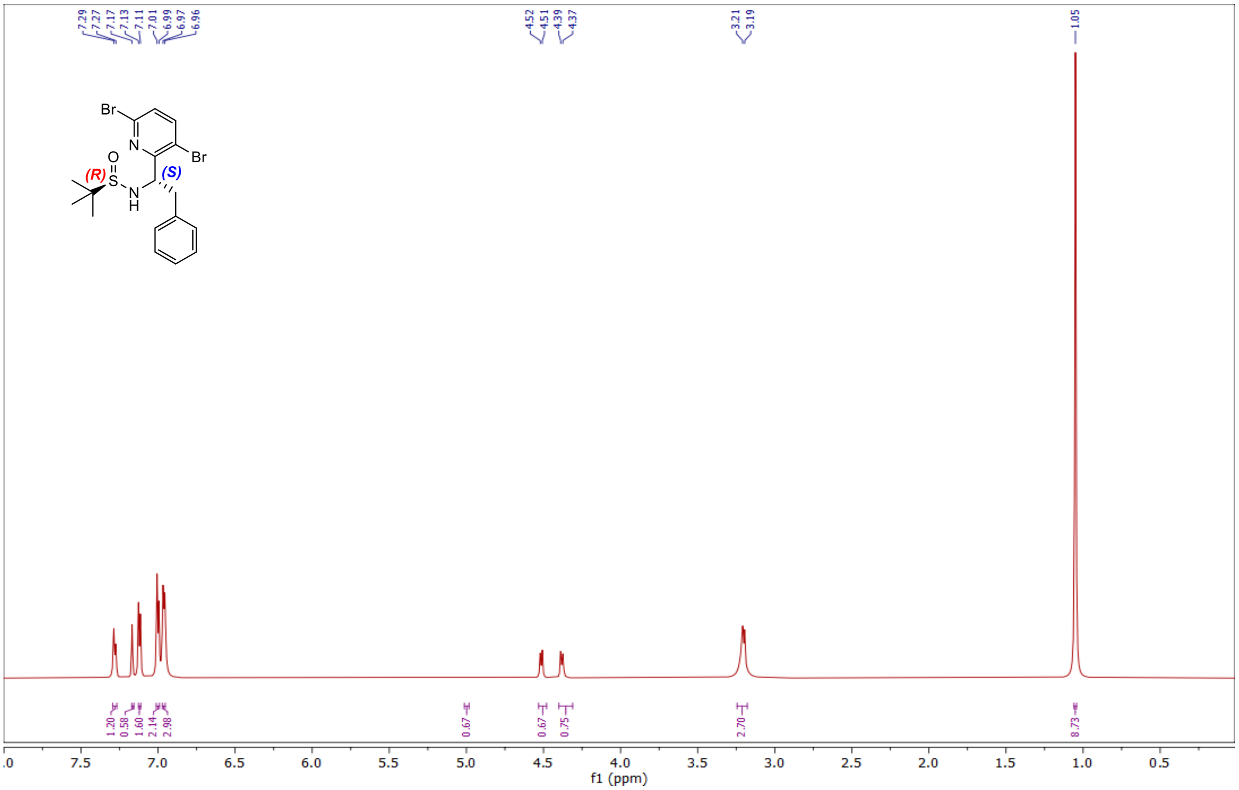

Figure S14: ^{1}H-NMR spectra of compound (**22b**).

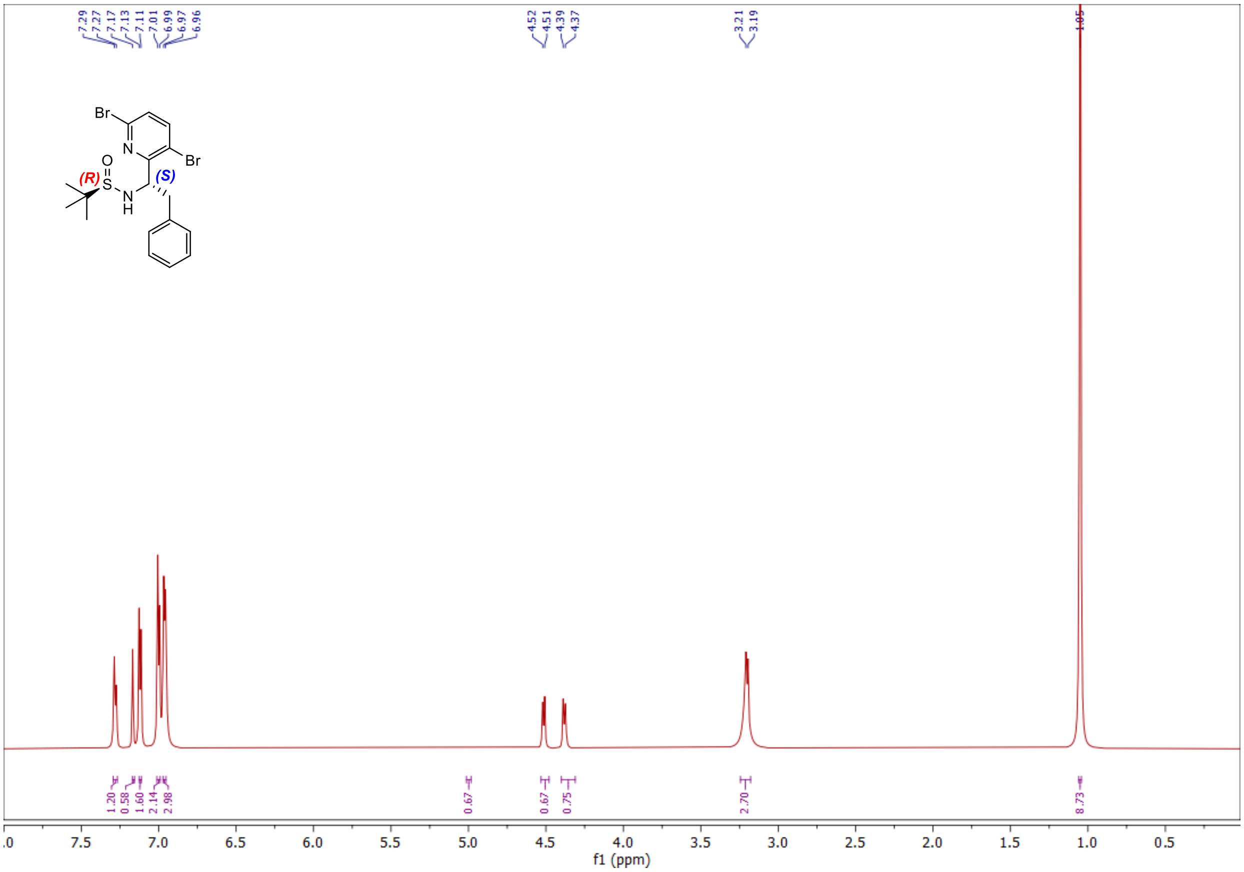

Figure S15: ^{1}H- and ^{13}C-NMR spectra of compound (**23a**).

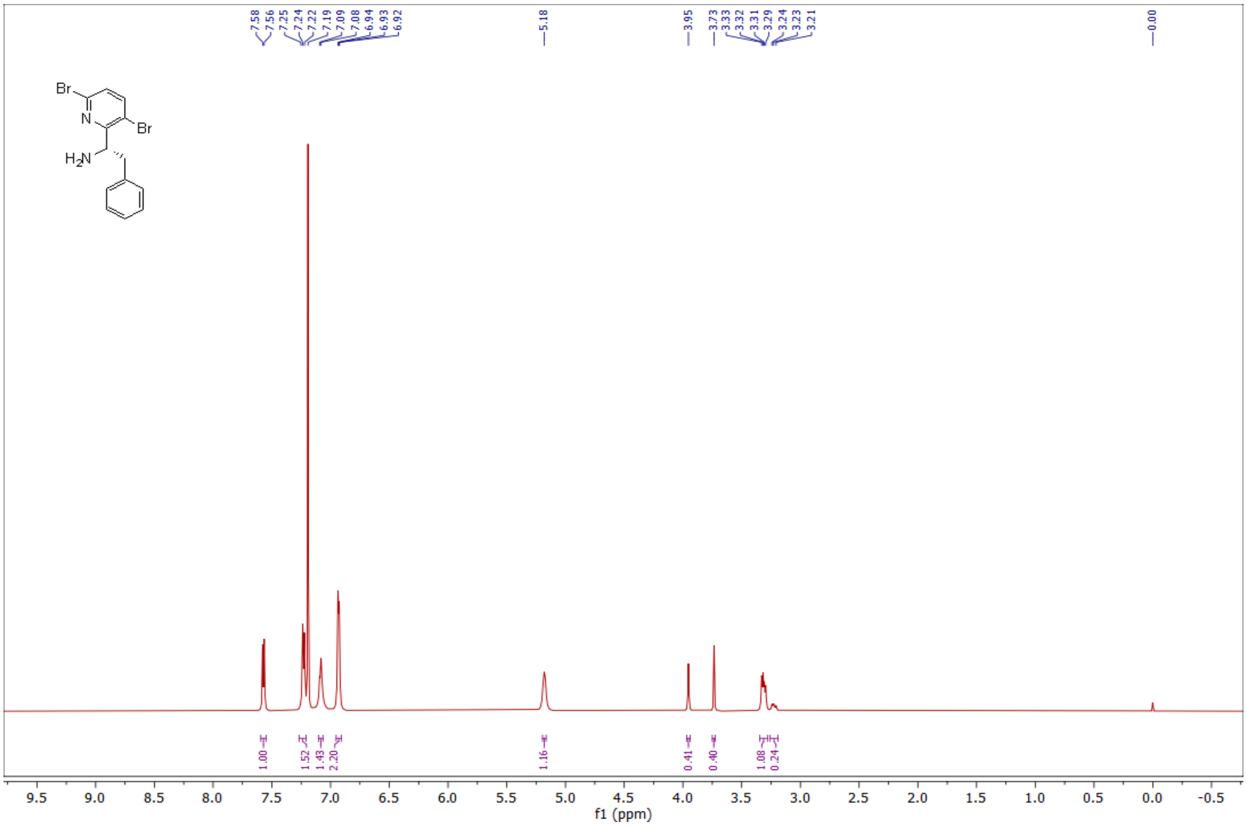

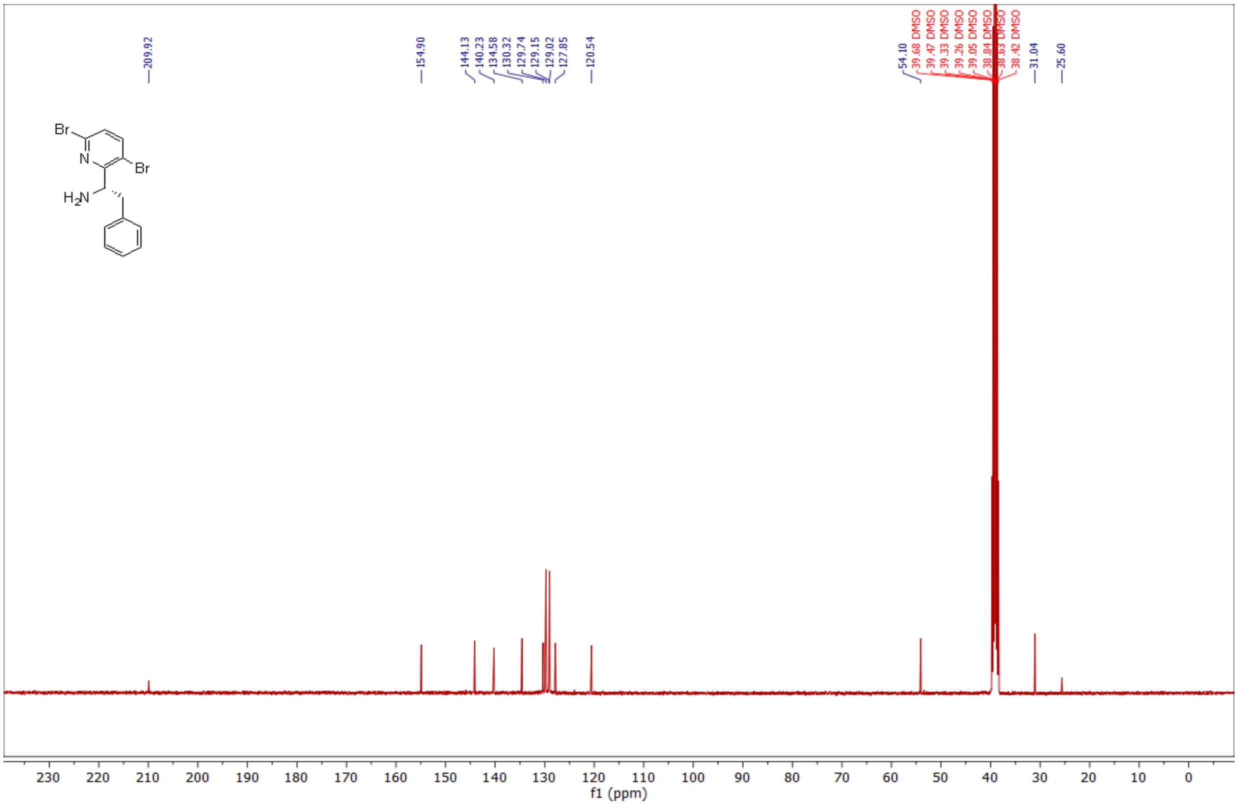

Figure S16: ^{1}H- and ^{13}C-NMR spectra of compound (**23b**).

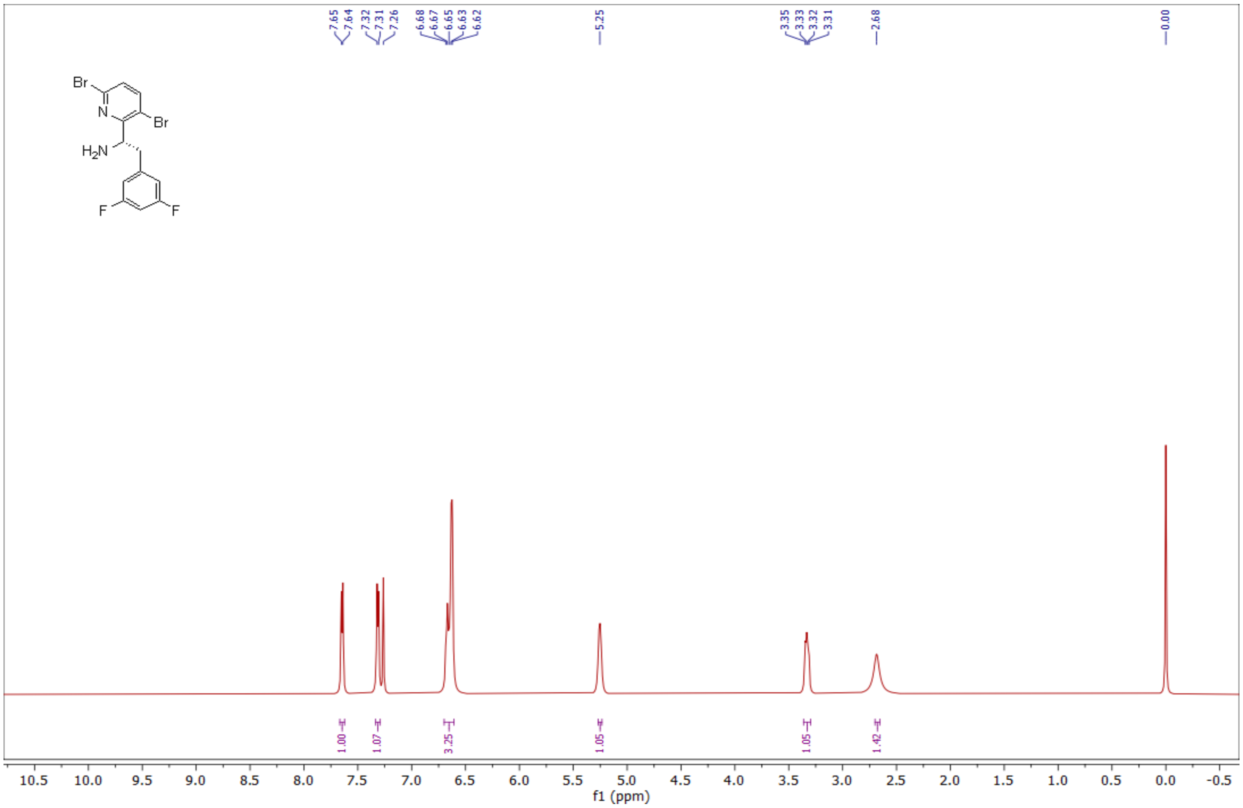

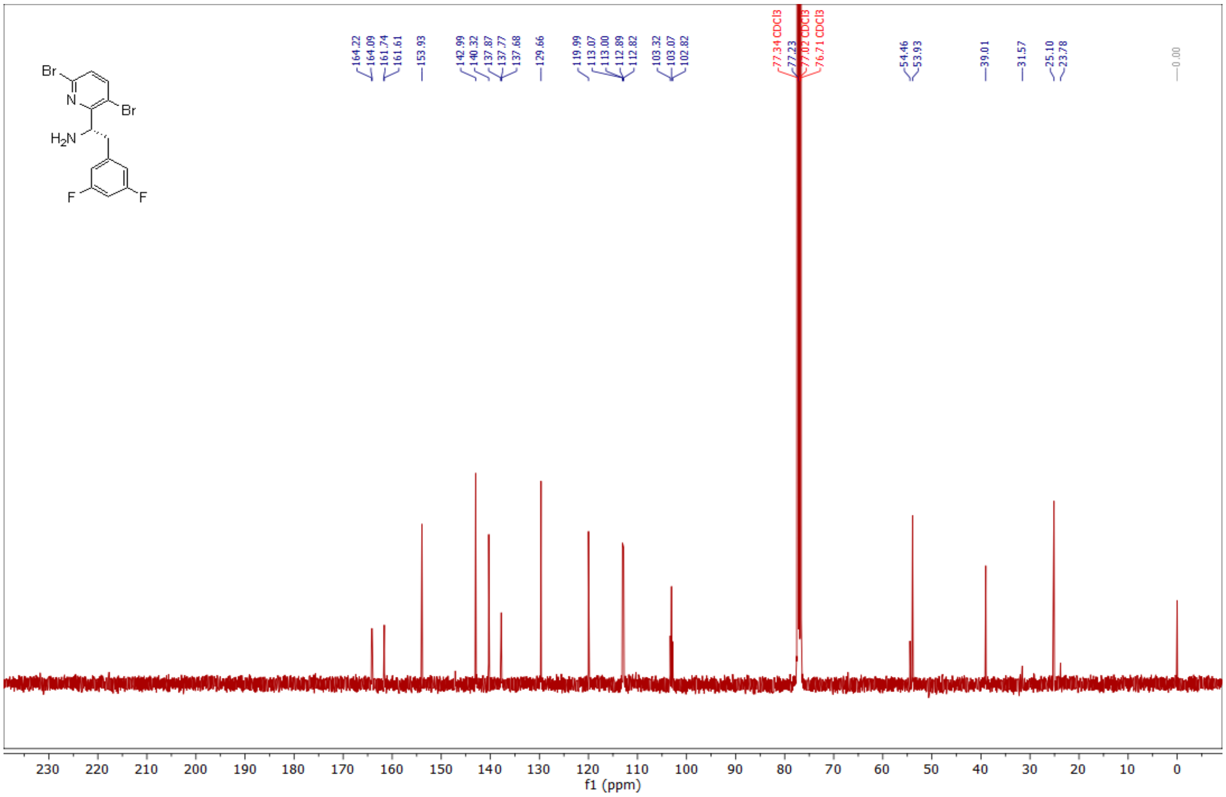

Figure S17: ^{1}H- and ^{13}C-NMR spectra of compound (**8a**).

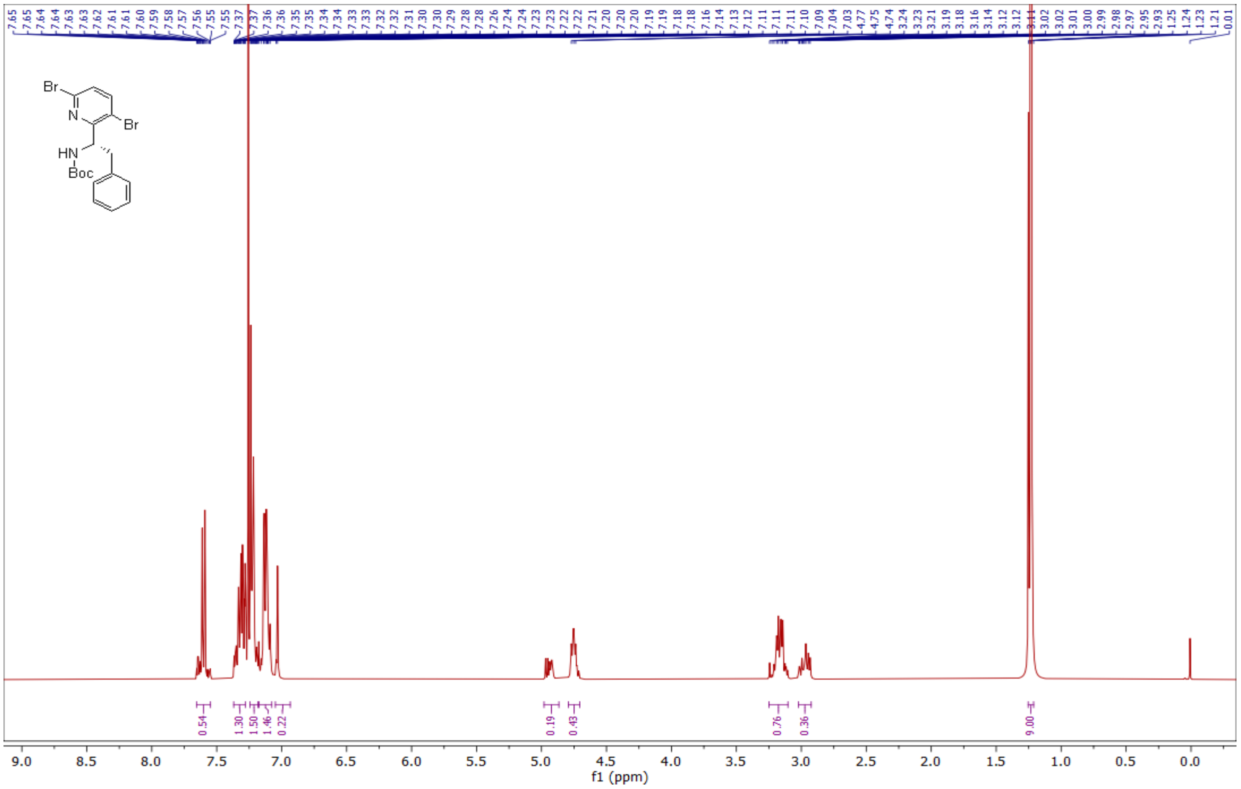

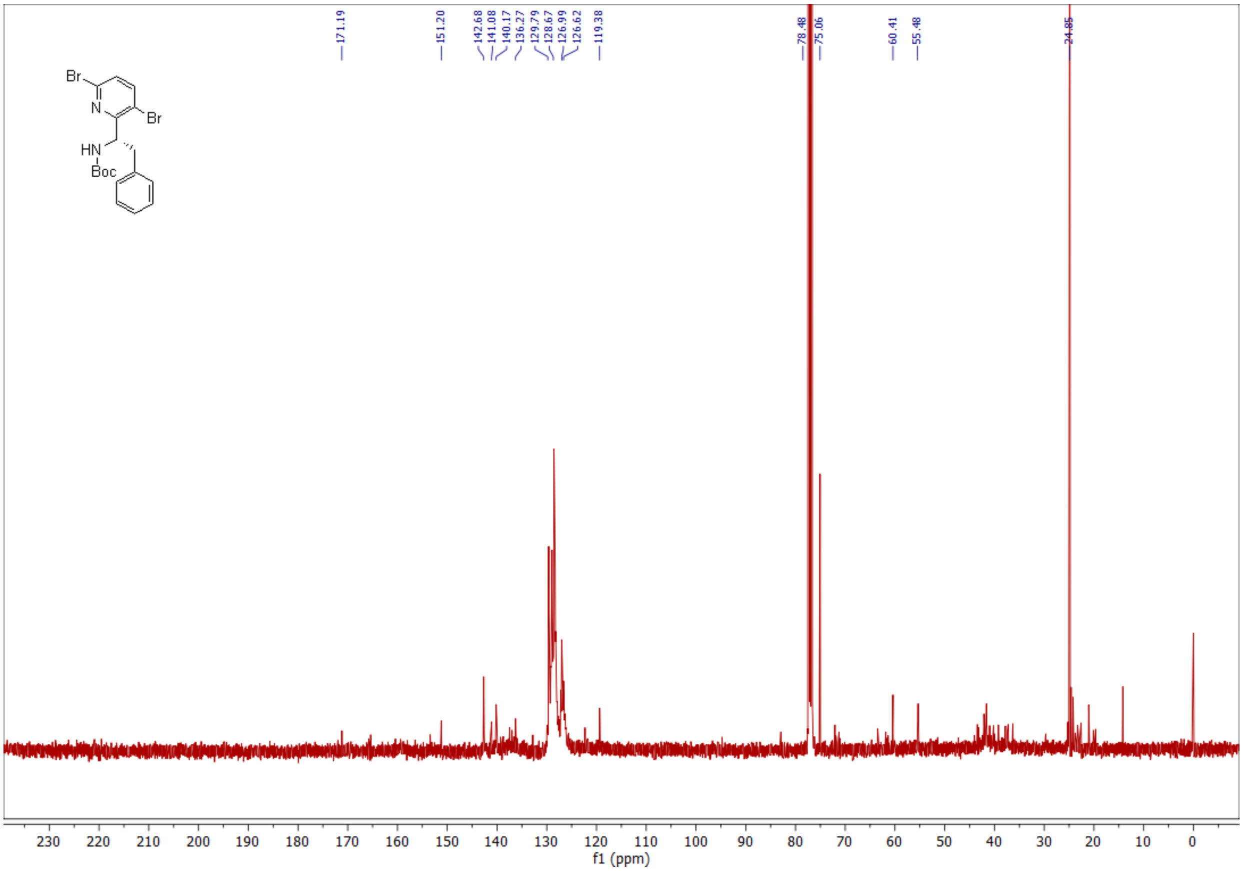

Figure S18: ^{1}H- and ^{13}C-NMR spectra of compound (**8b**).

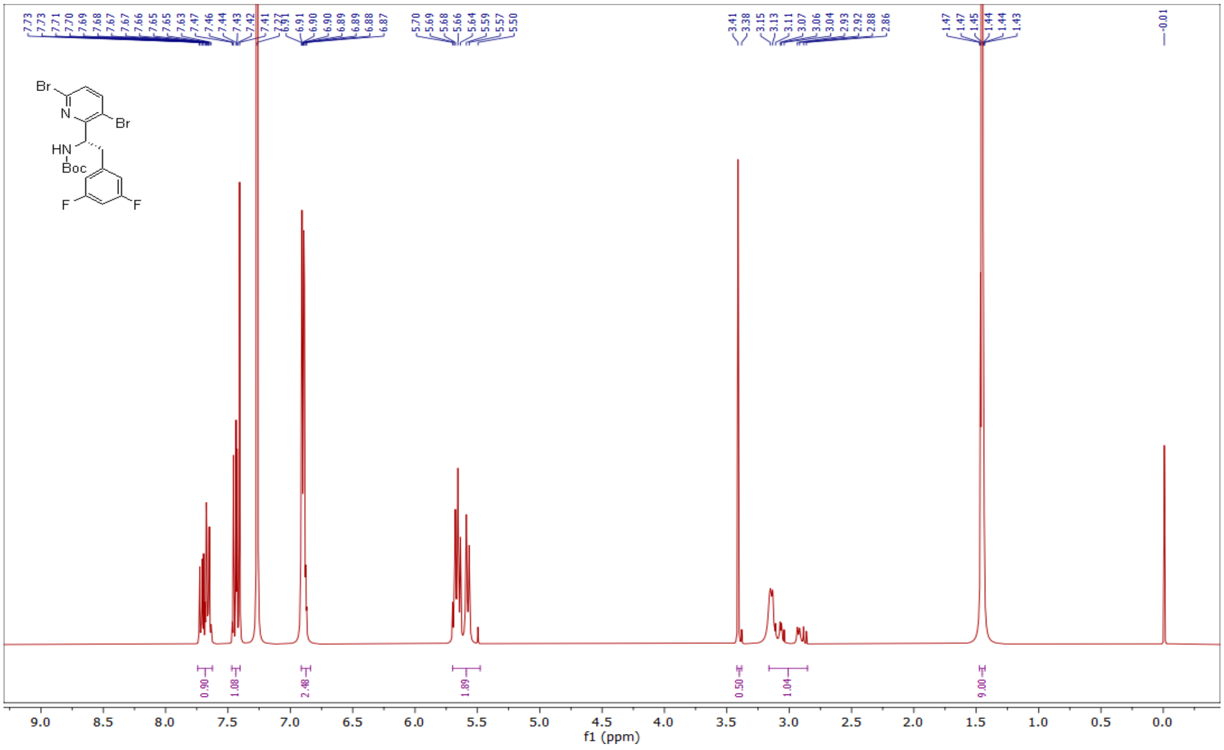

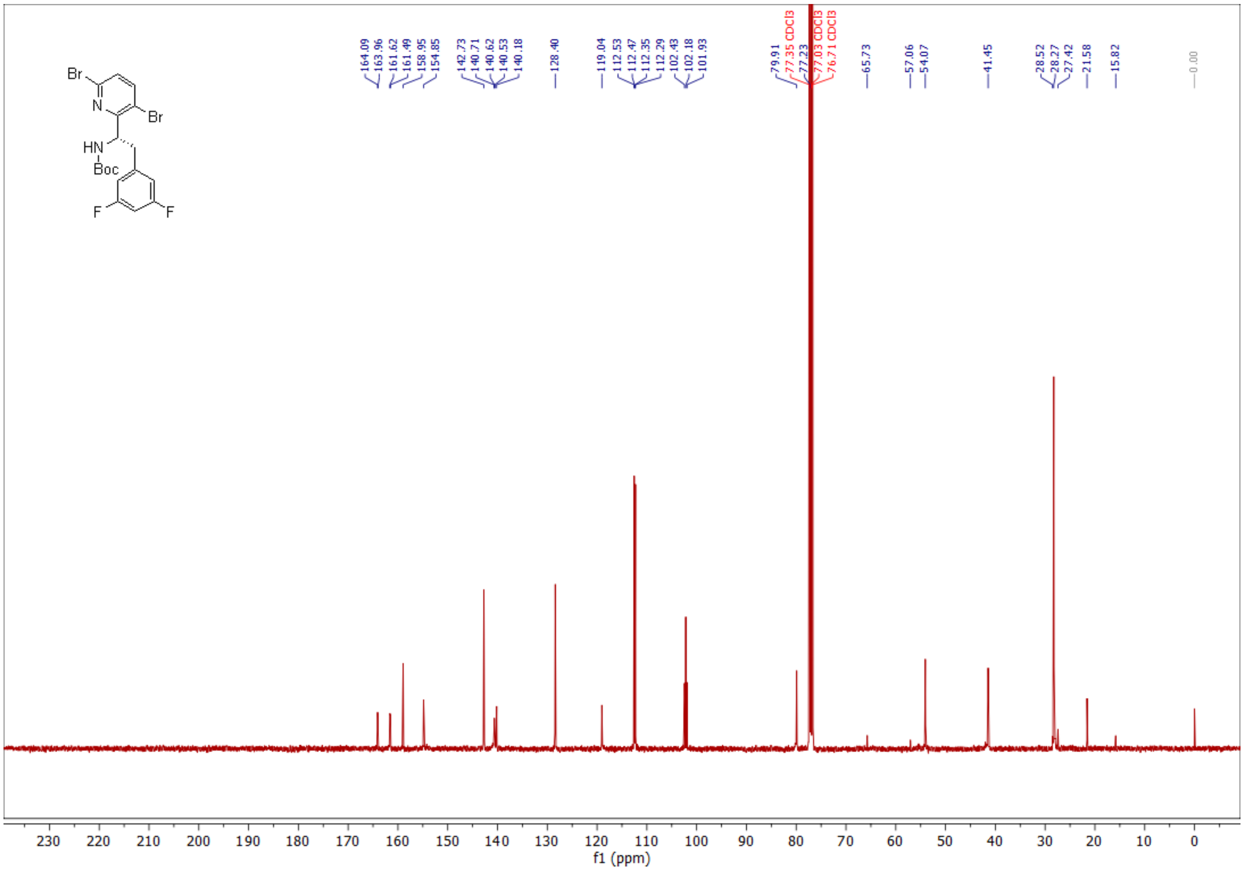

Figure S19: ^{1}H- and ^{13}C-NMR spectra of compound (**10**).

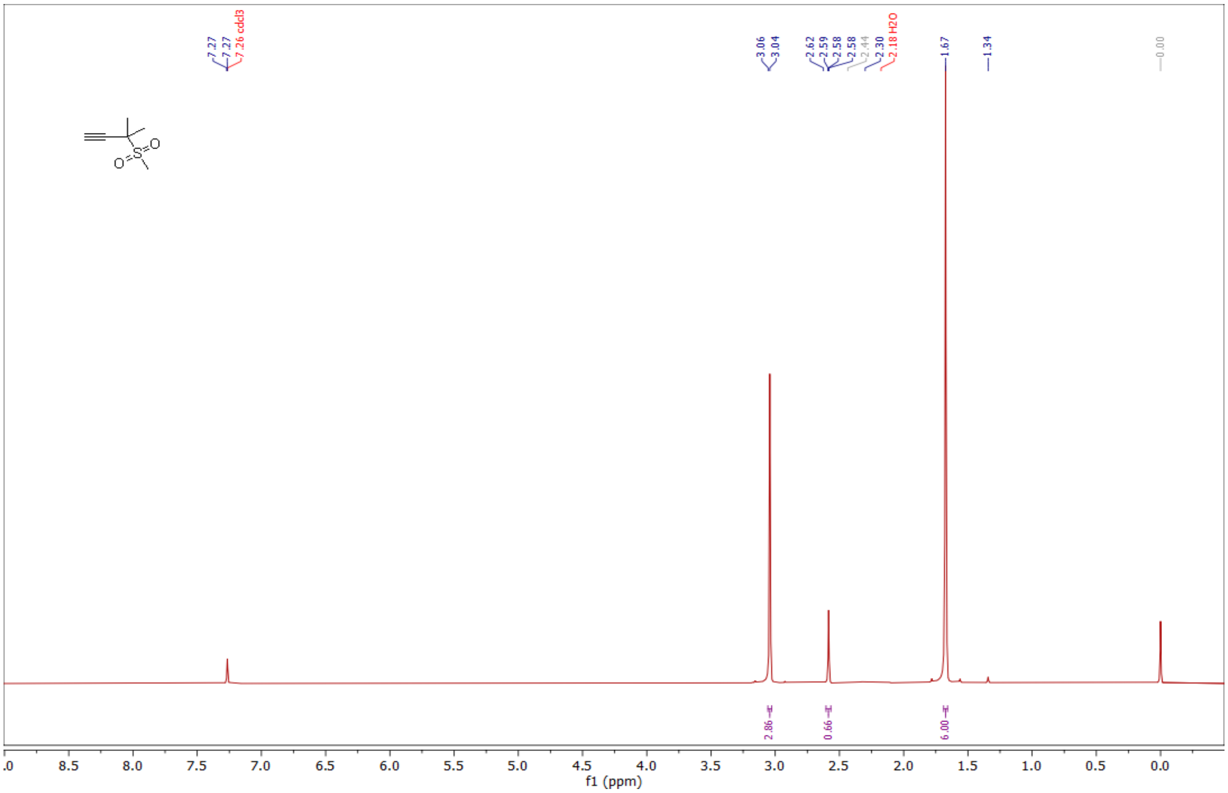

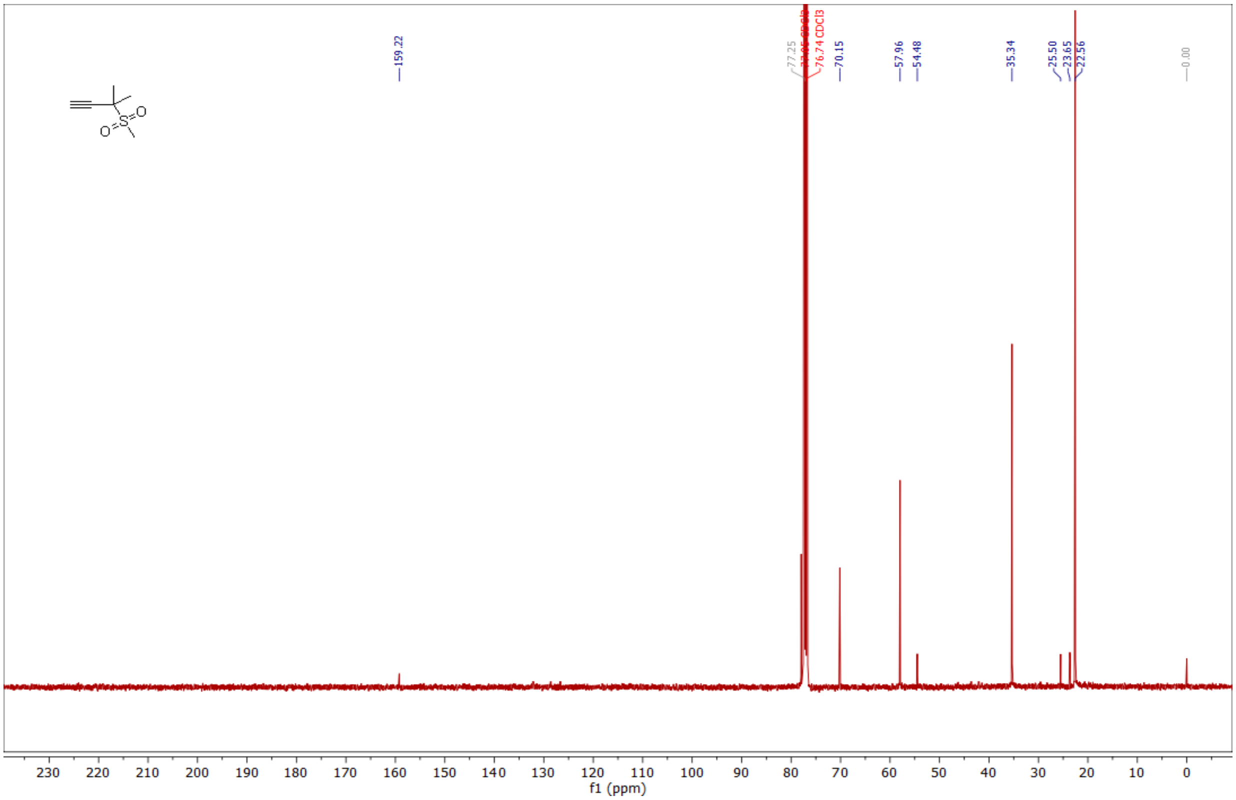

Figure S20: ^{1}H- and ^{13}C-NMR spectra of compound (**27a**).

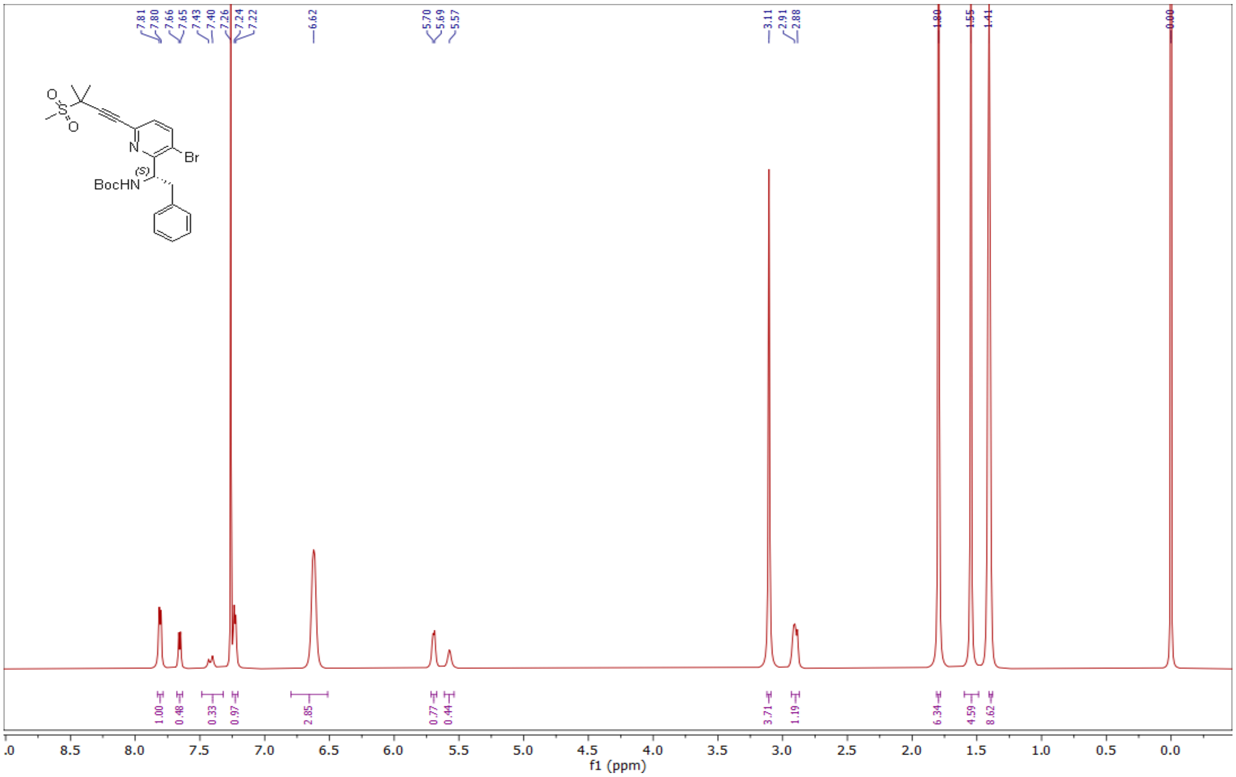

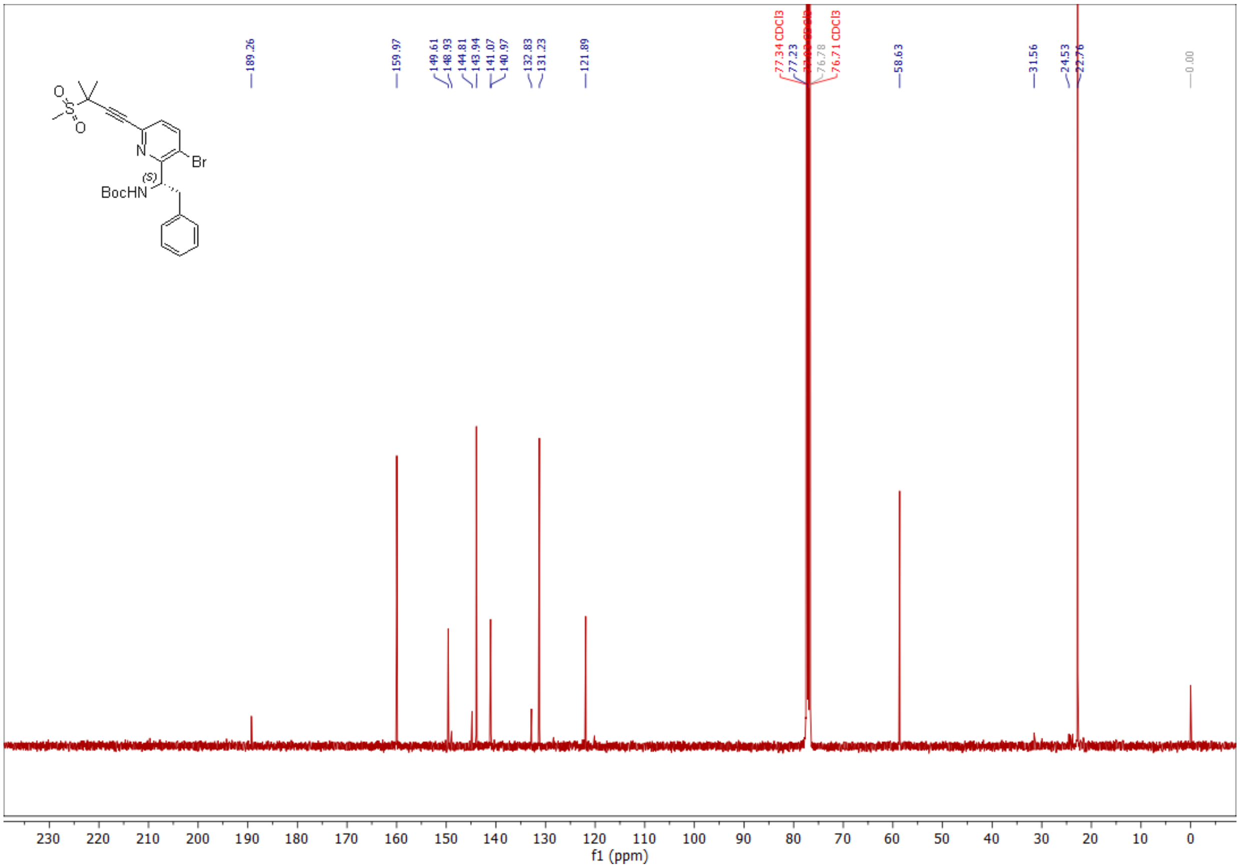

Figure S21: ^{1}H- and ^{13}C-NMR spectra of compound (**27b**).

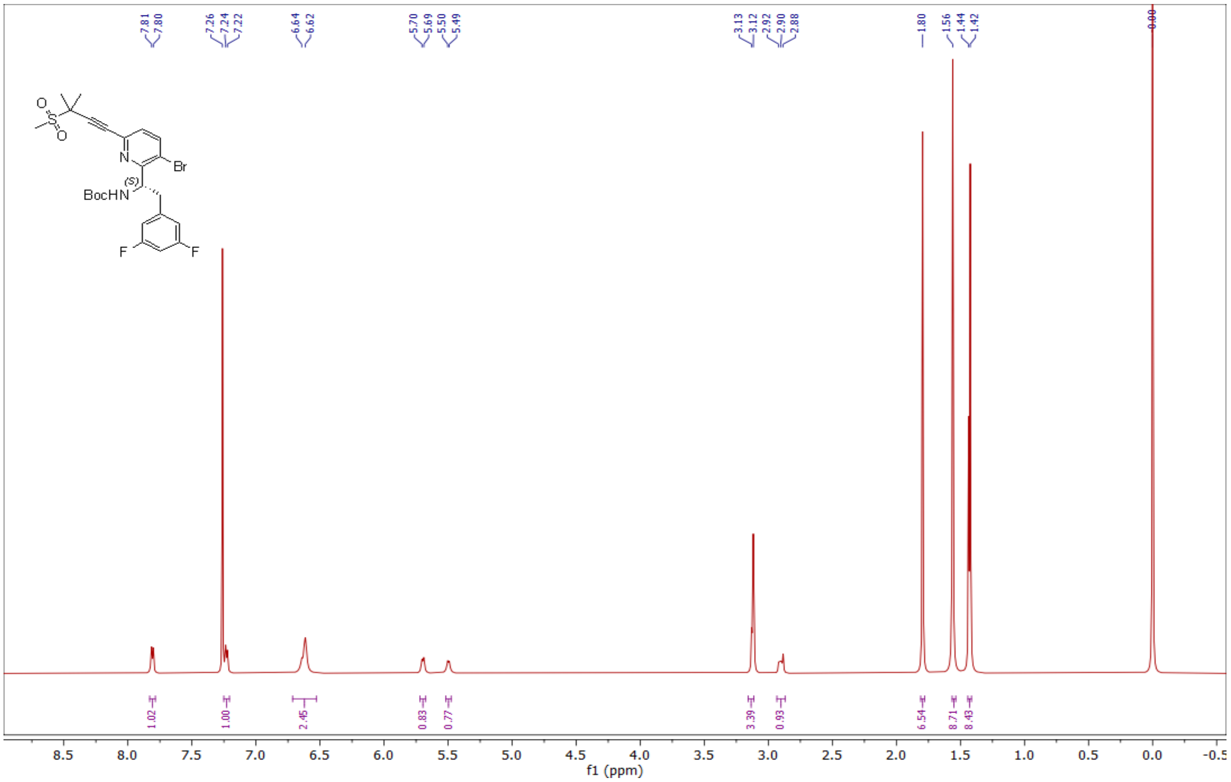

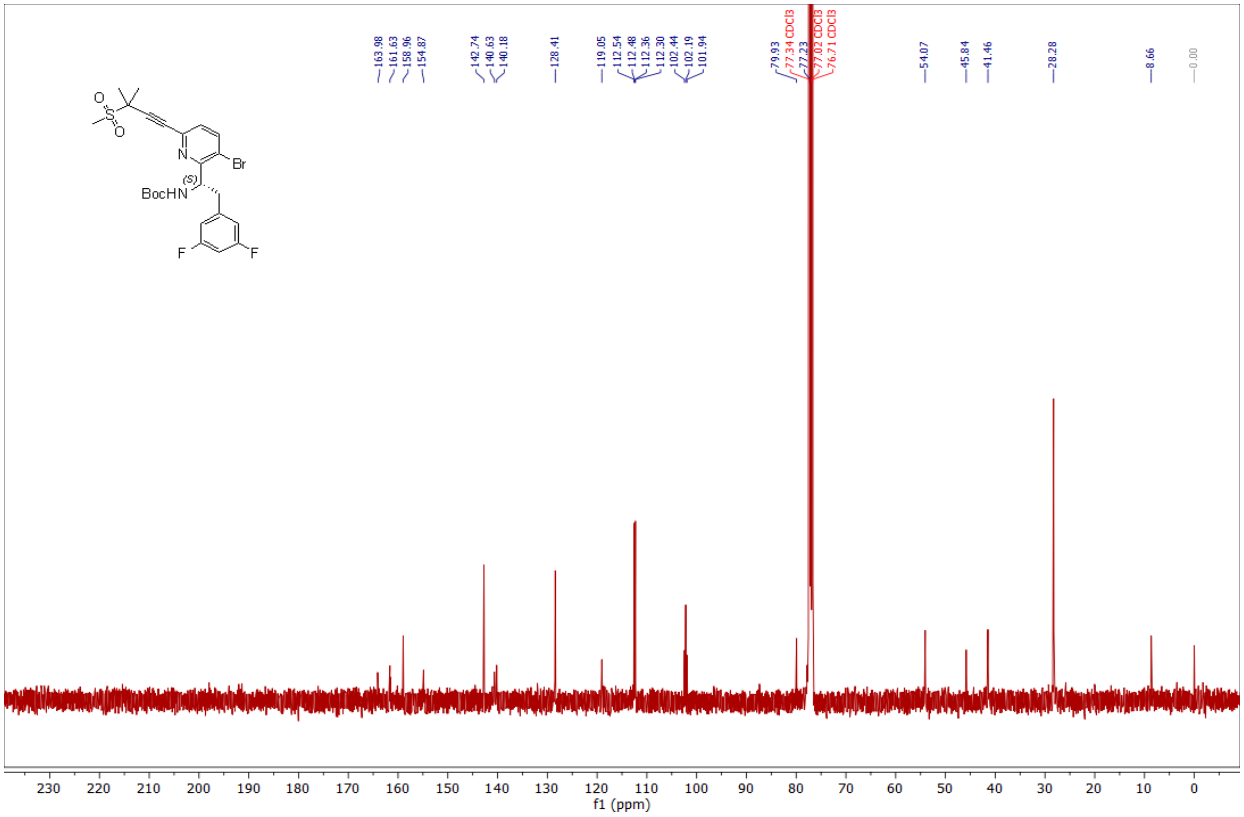

Figure S22: ^{1}H-NMR spectra of compound (**28a**).

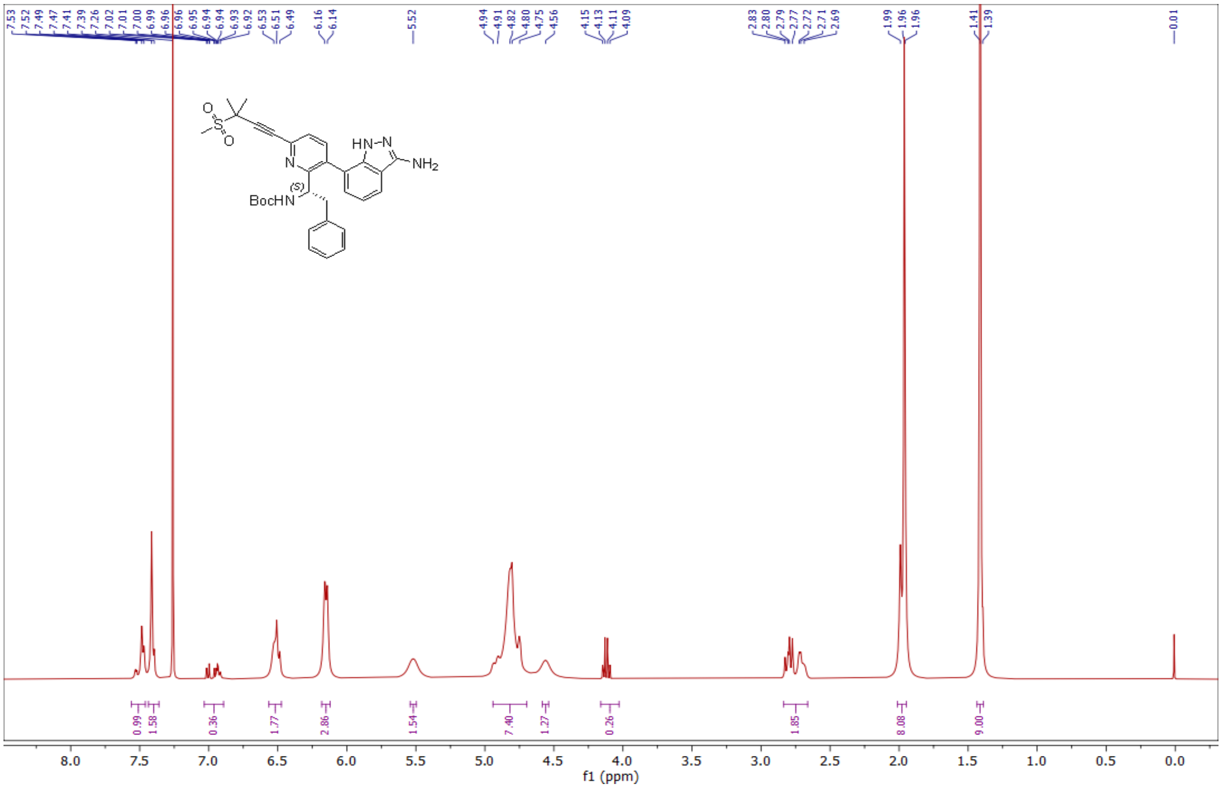

Figure S23: ^{1}H- and ^{13}C-NMR spectra of compound (**28b**).

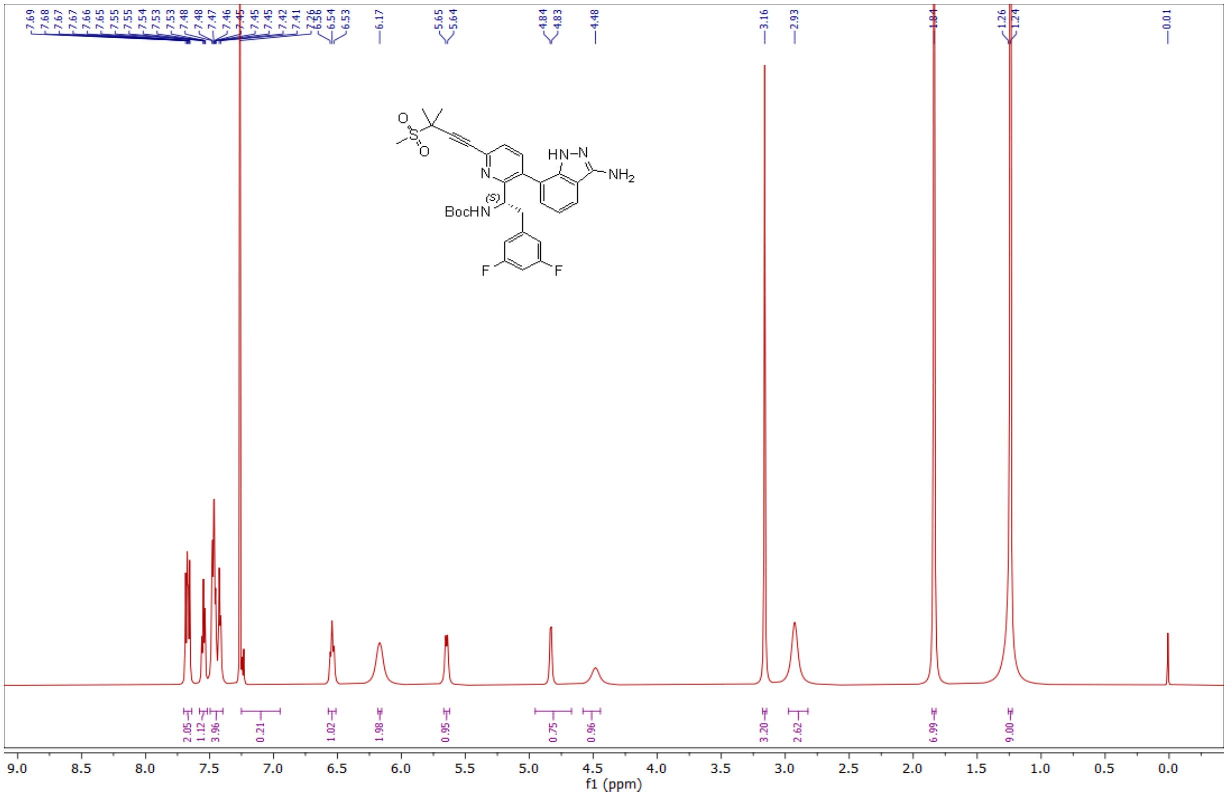

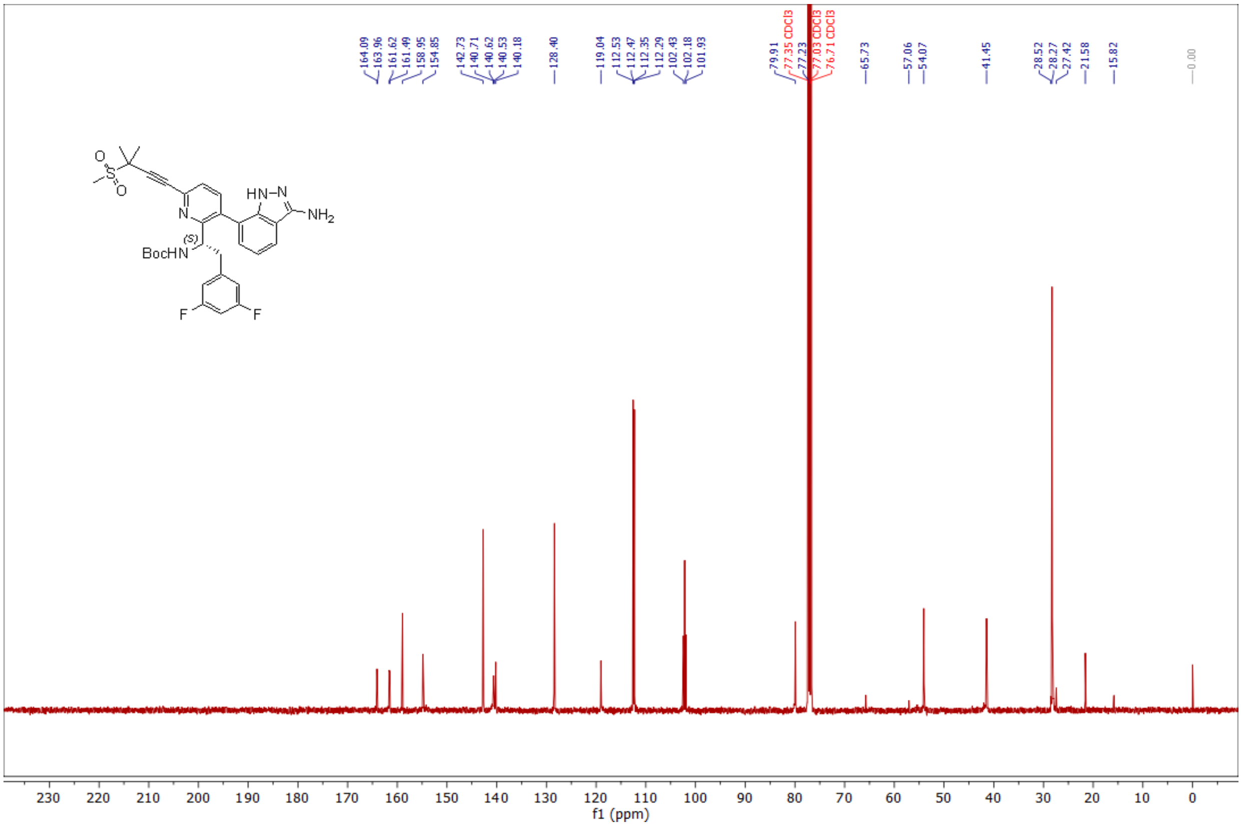

Figure S24: ^{1}H- and ^{13}C-NMR spectra of compound (**29a**).

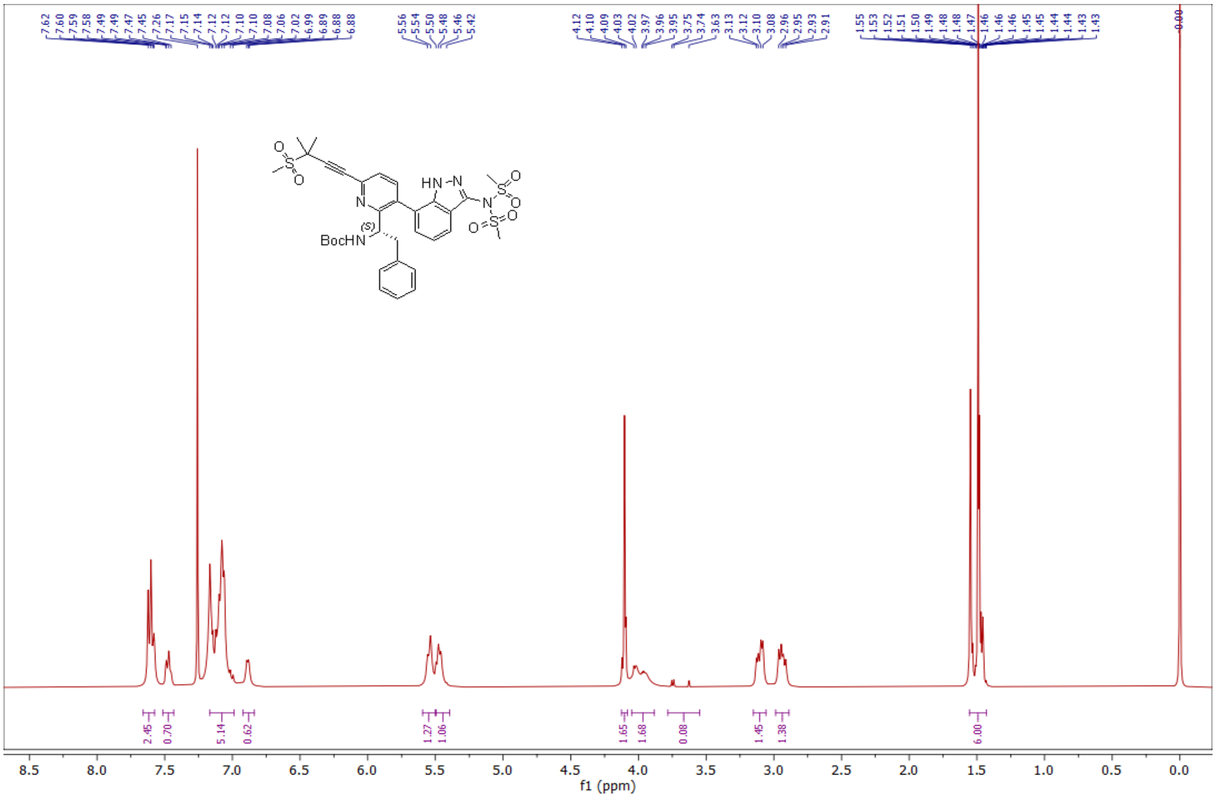

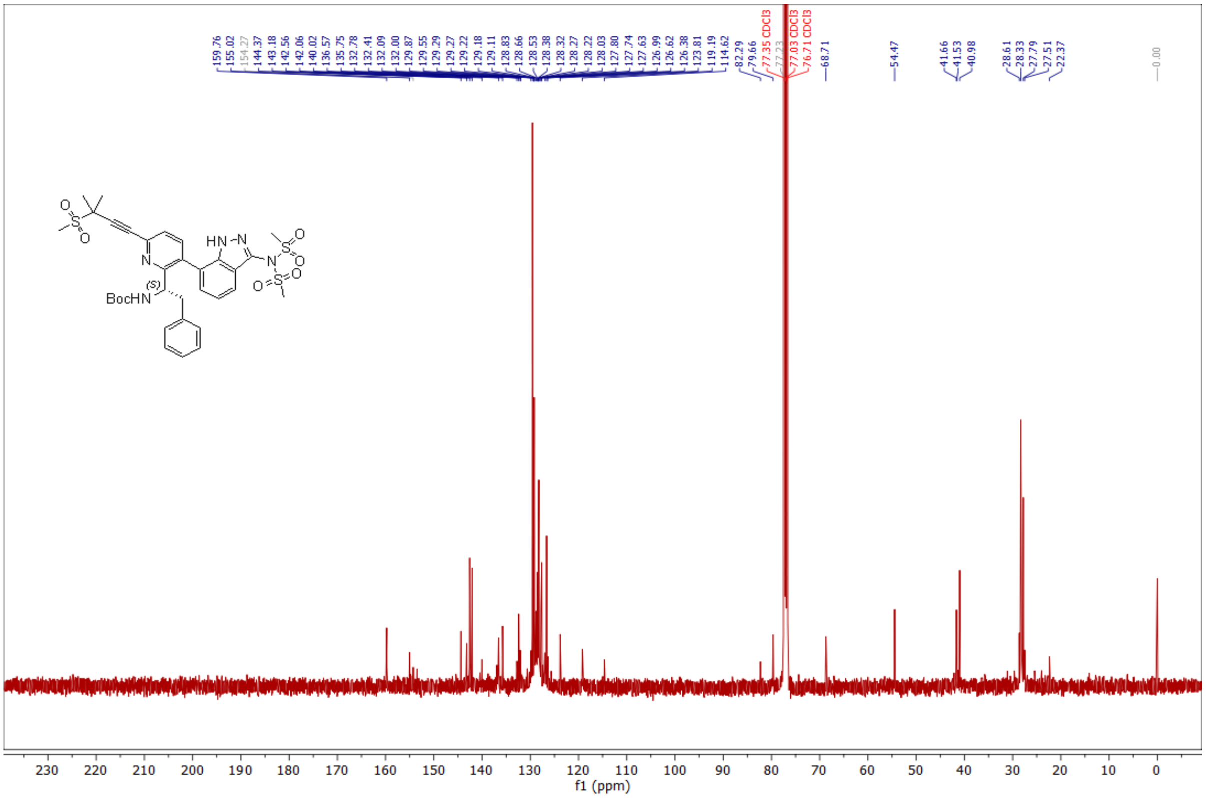

Figure S25: ^{1}H-NMR spectra of compound (**29b**).

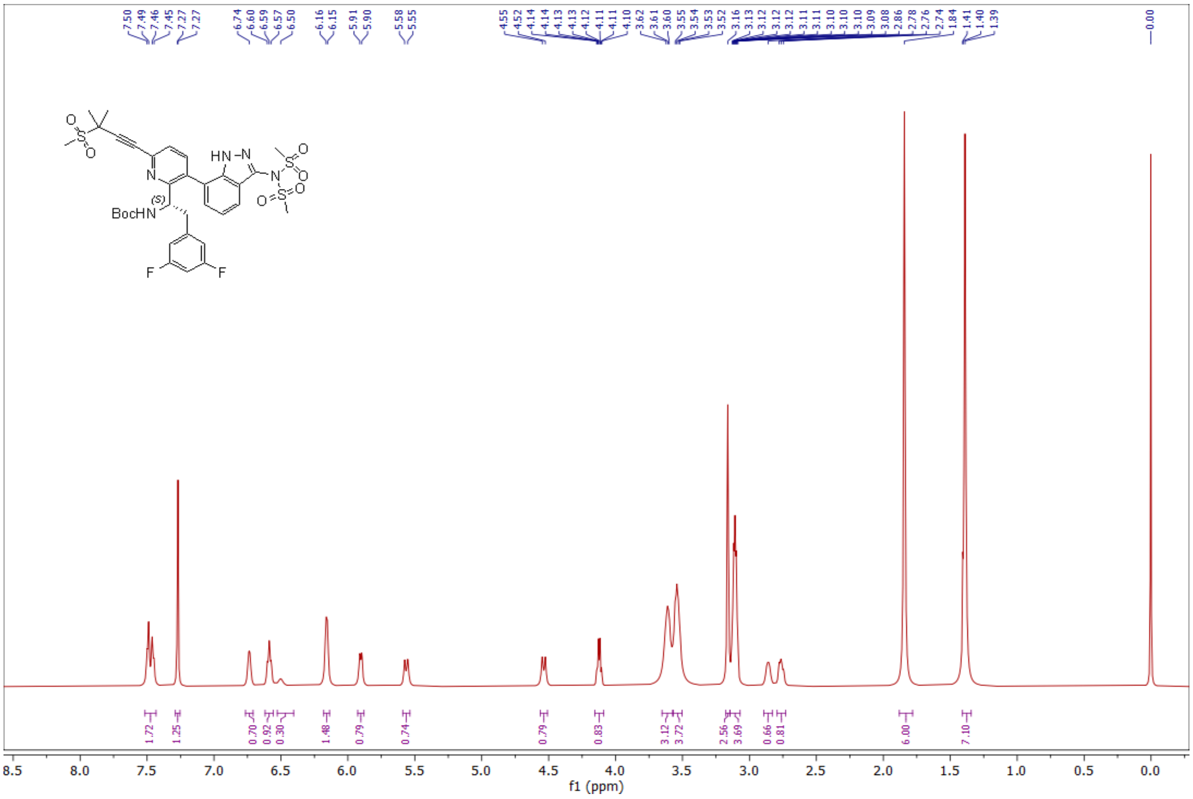

Figure S26: ^{1}H-NMR spectra of compound (**30a**).

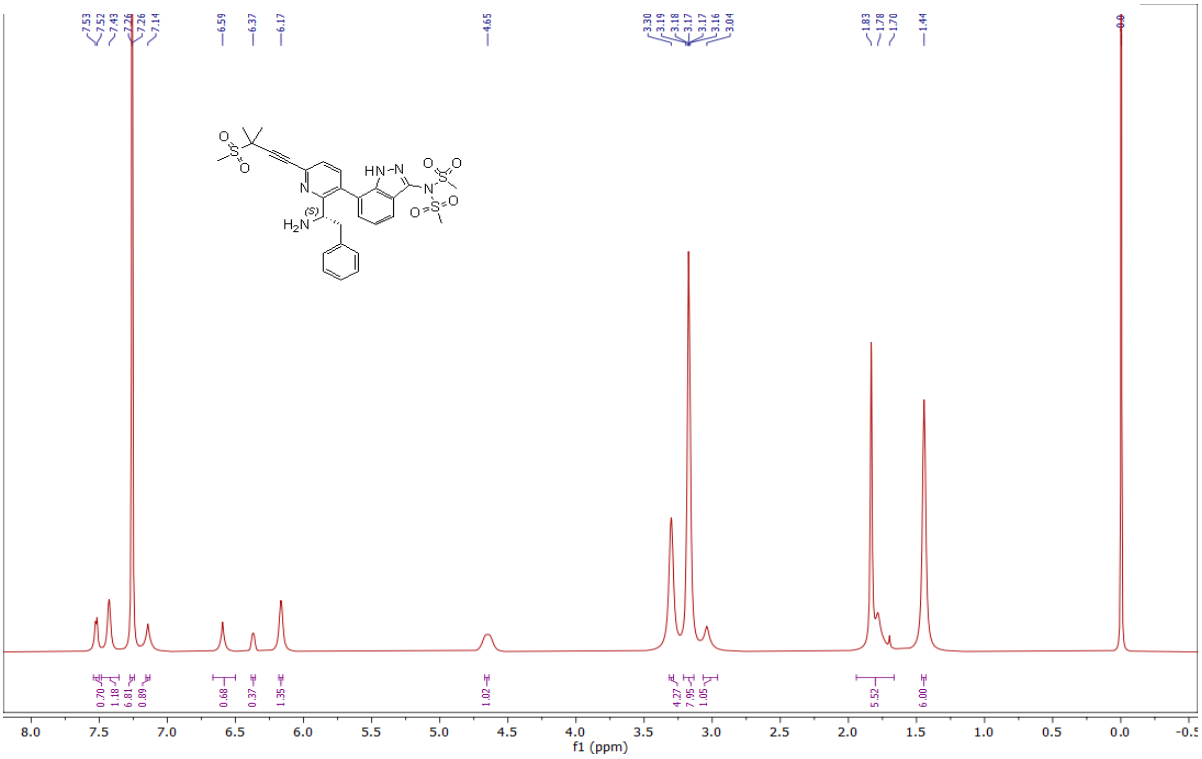

Figure S27: ^{1}H- and ^{13}C-NMR spectra of compound (**30b**).

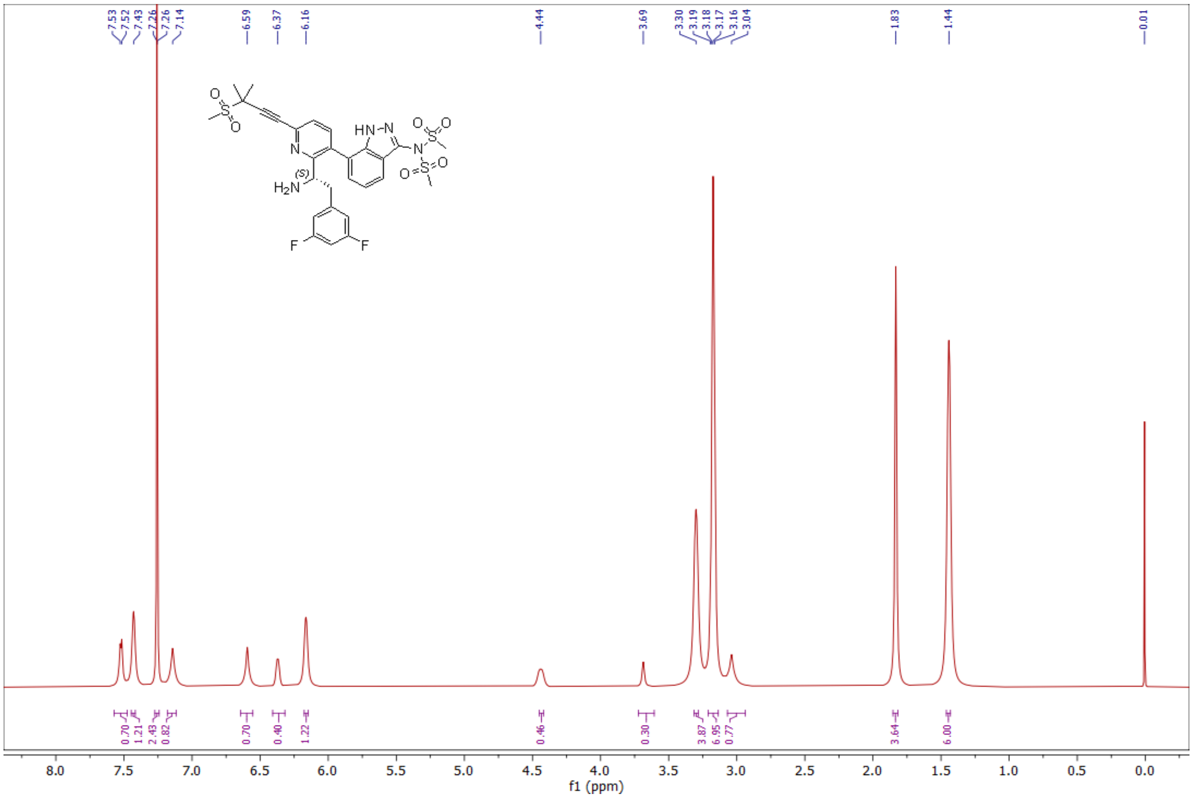

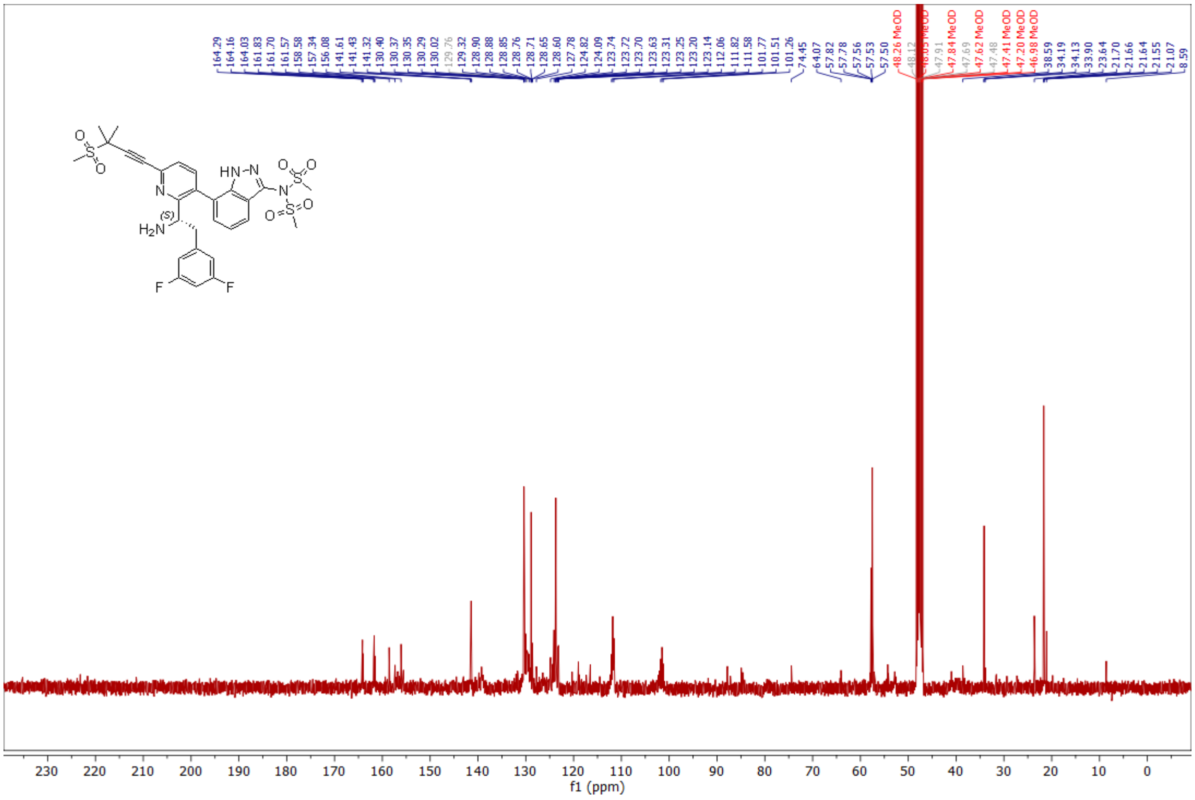

Figure S28: ^{1}H- and ^{13}C-NMR spectra of compound (**6a**).

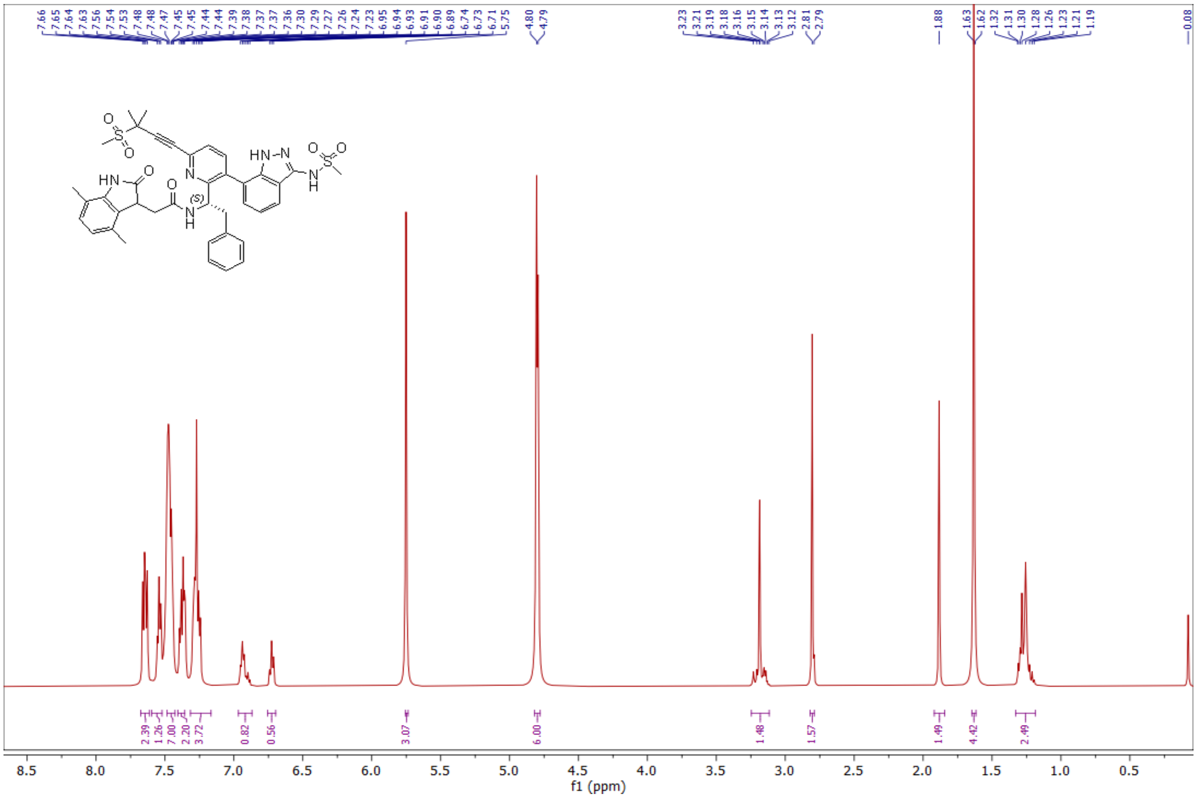

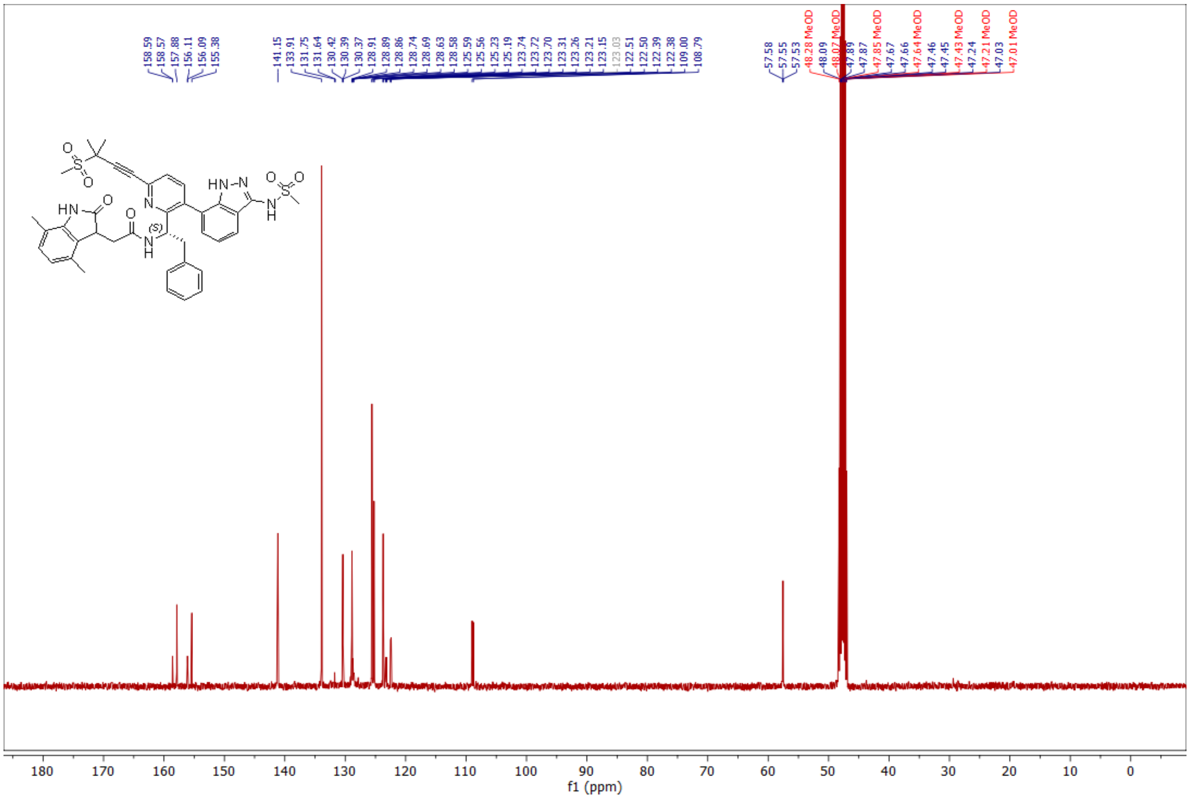

Figure S29: ^{1}H- and ^{13}C-NMR spectra of compound (**6b**).

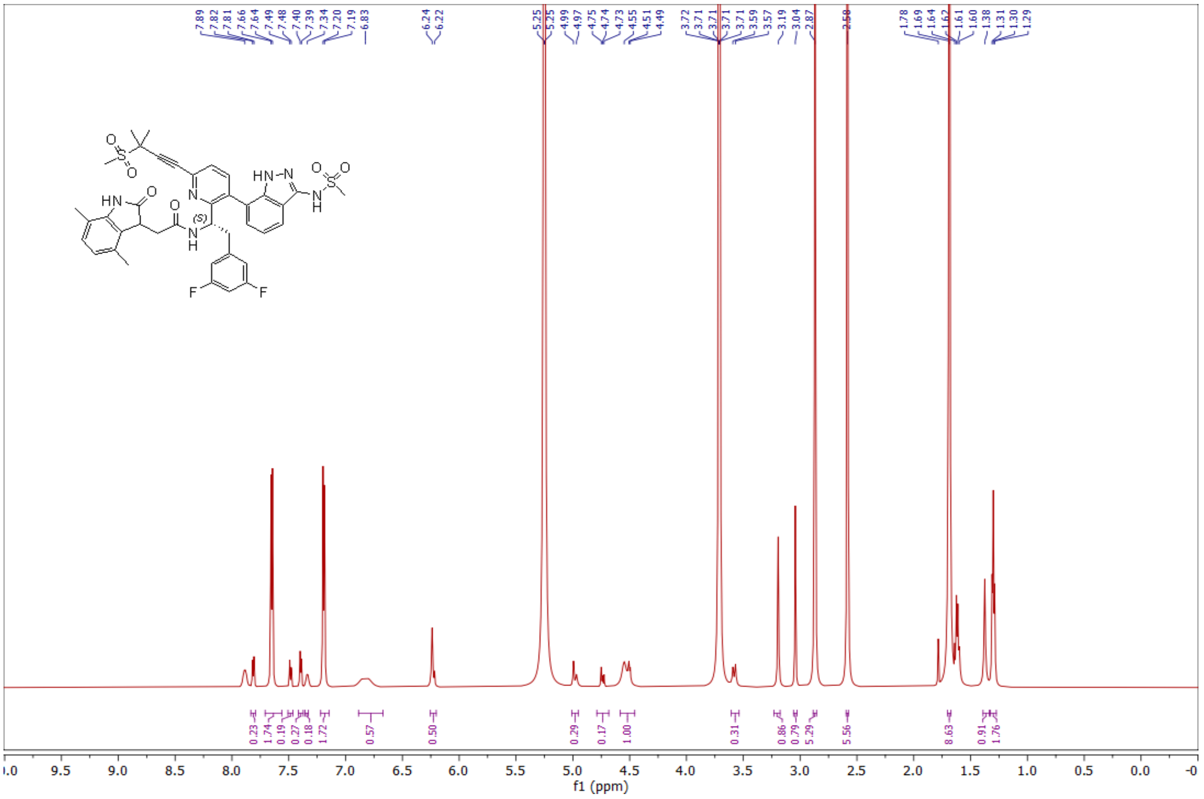

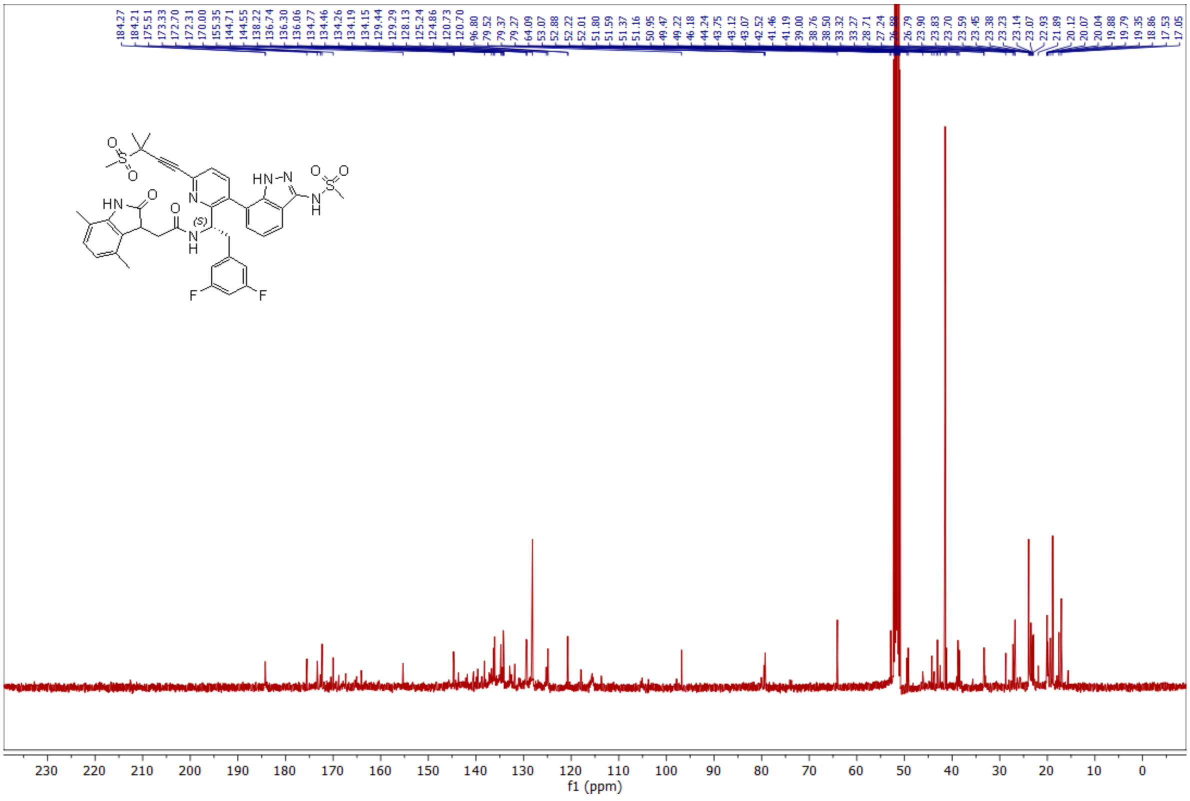

Figure S30: ^{1}H- and ^{13}C-NMR spectra of compound (**5a**).

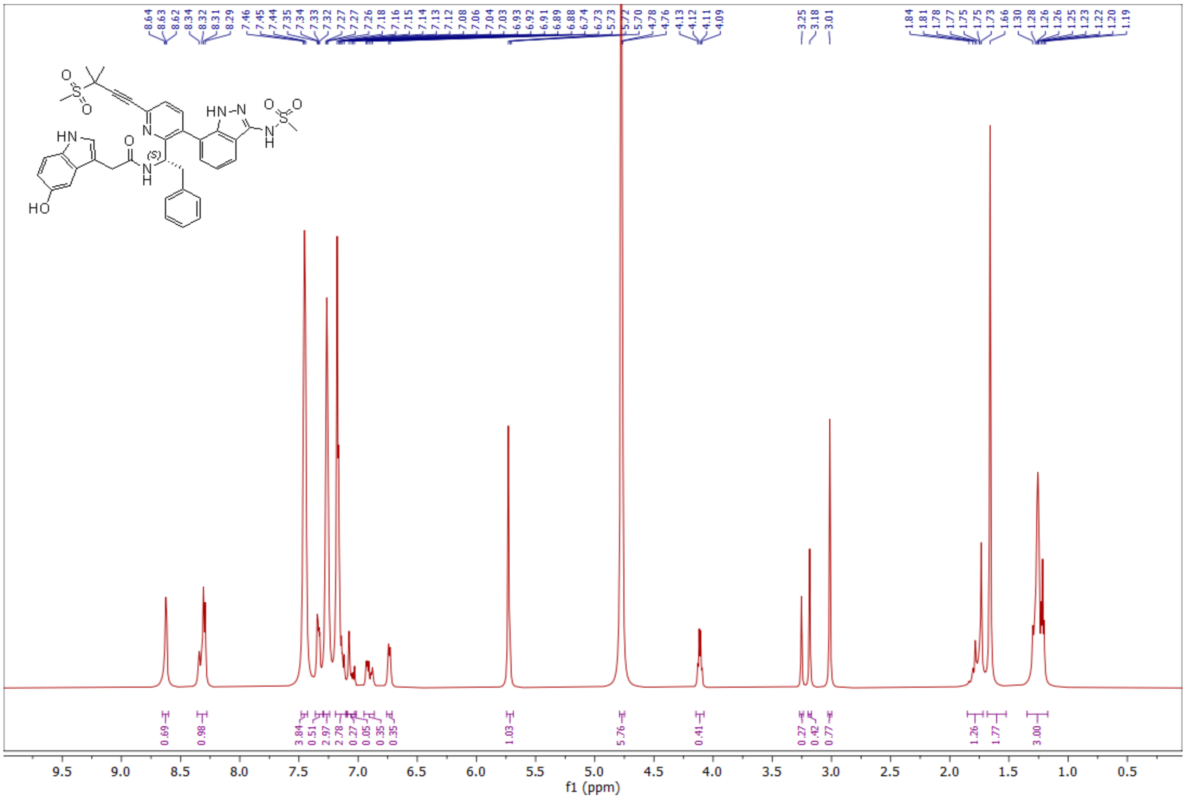

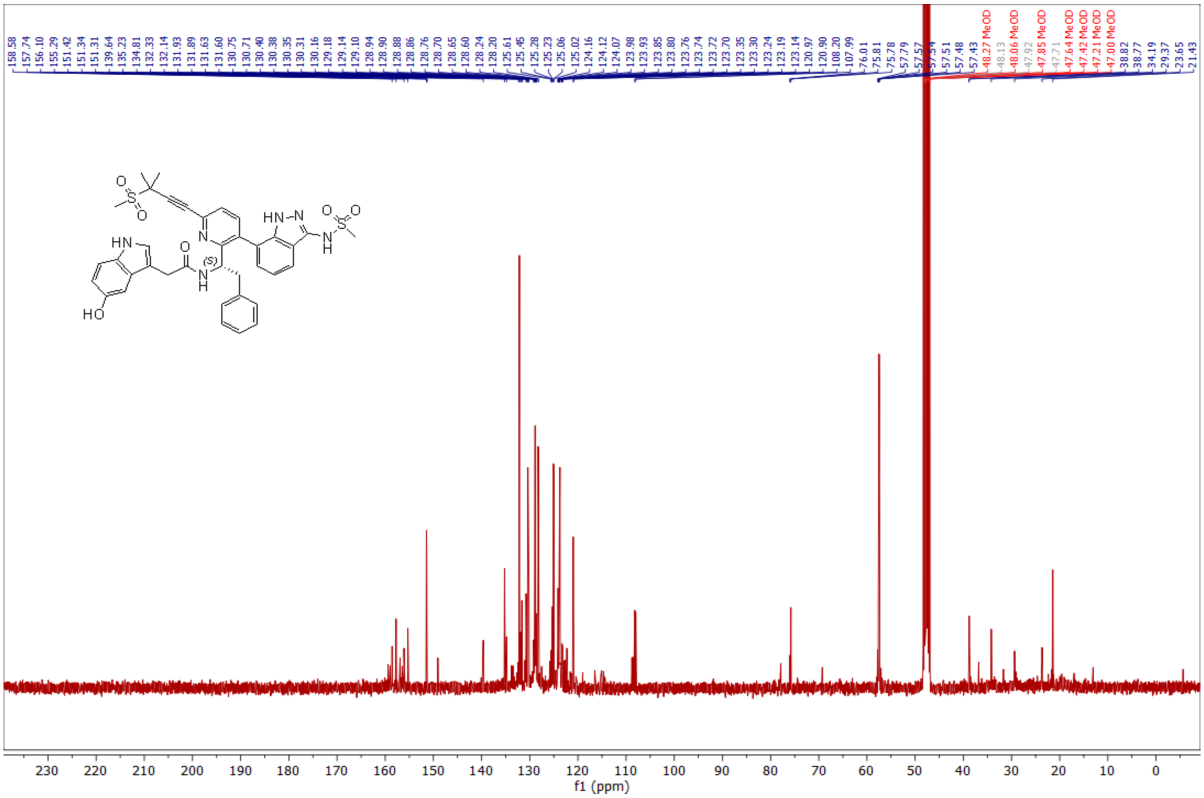

Figure S31: ^{1}H- and ^{13}C-NMR spectra of compound (**5b**).

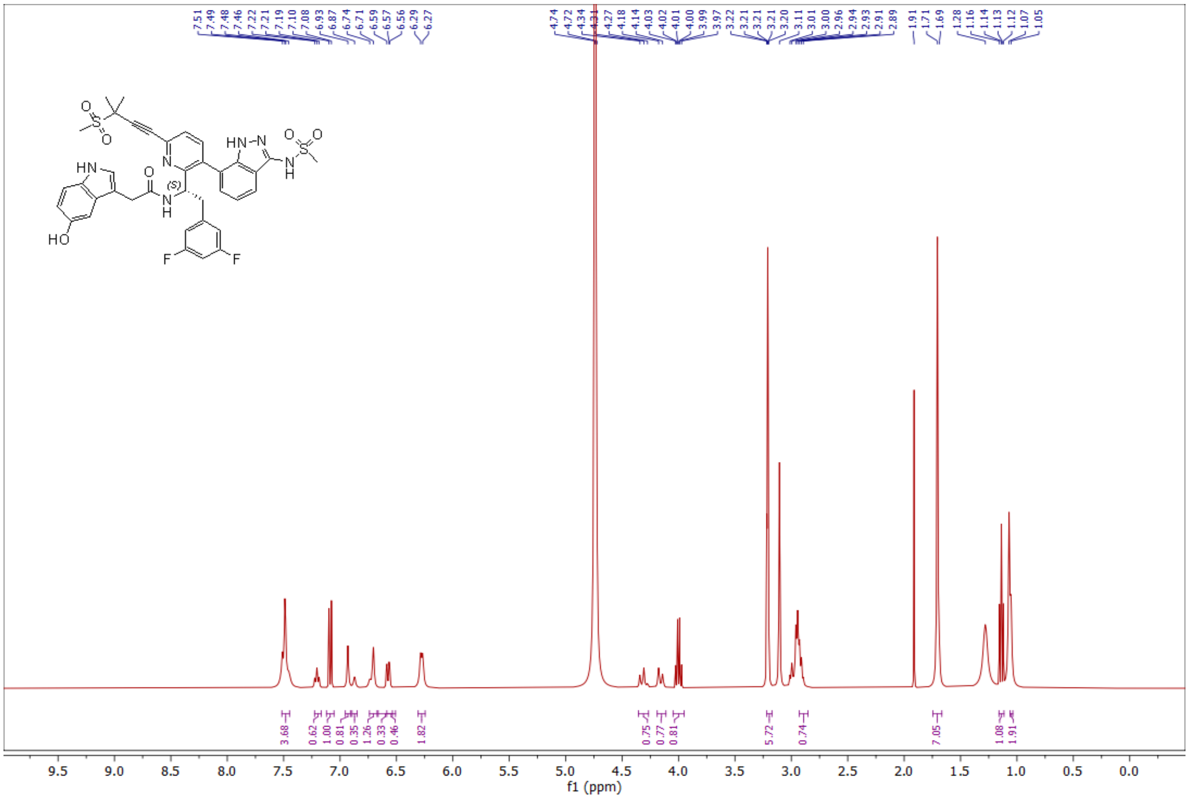

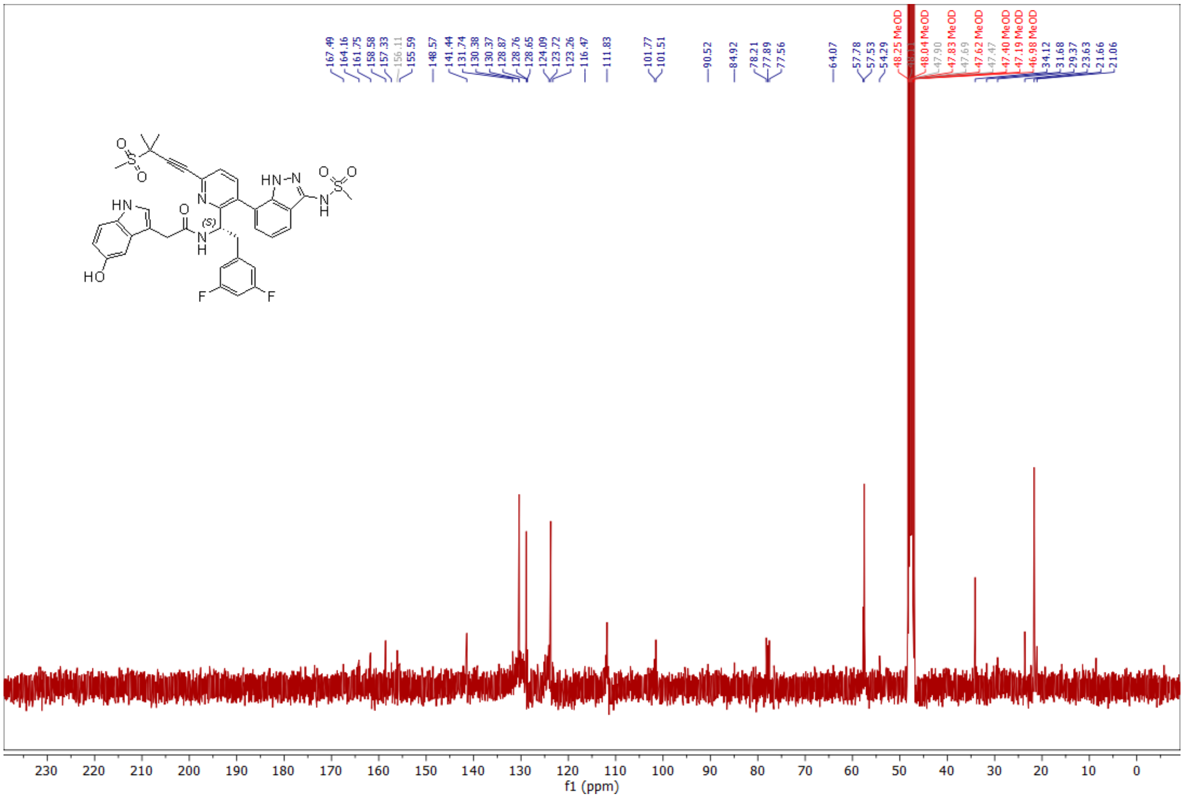

Figure S32: X-Ray spectra of compound (**22a**).

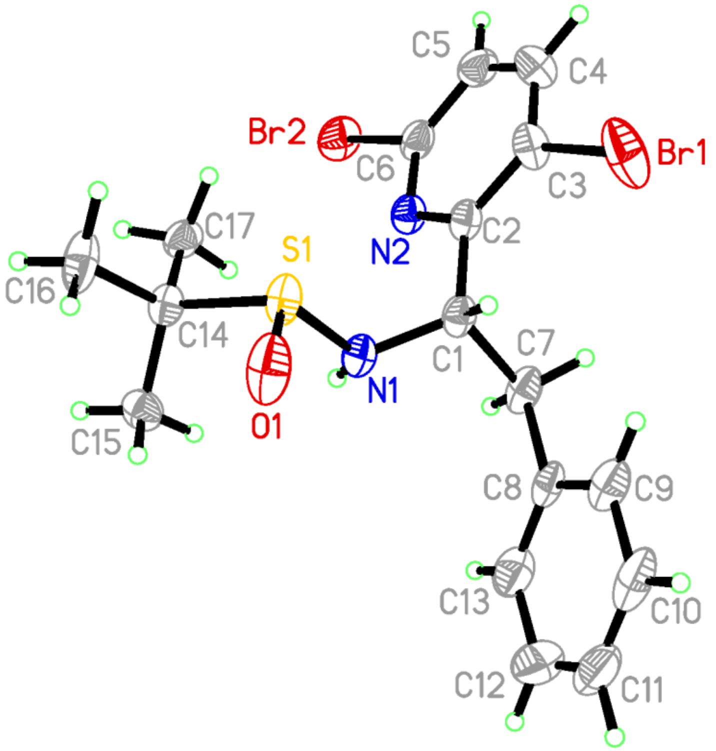

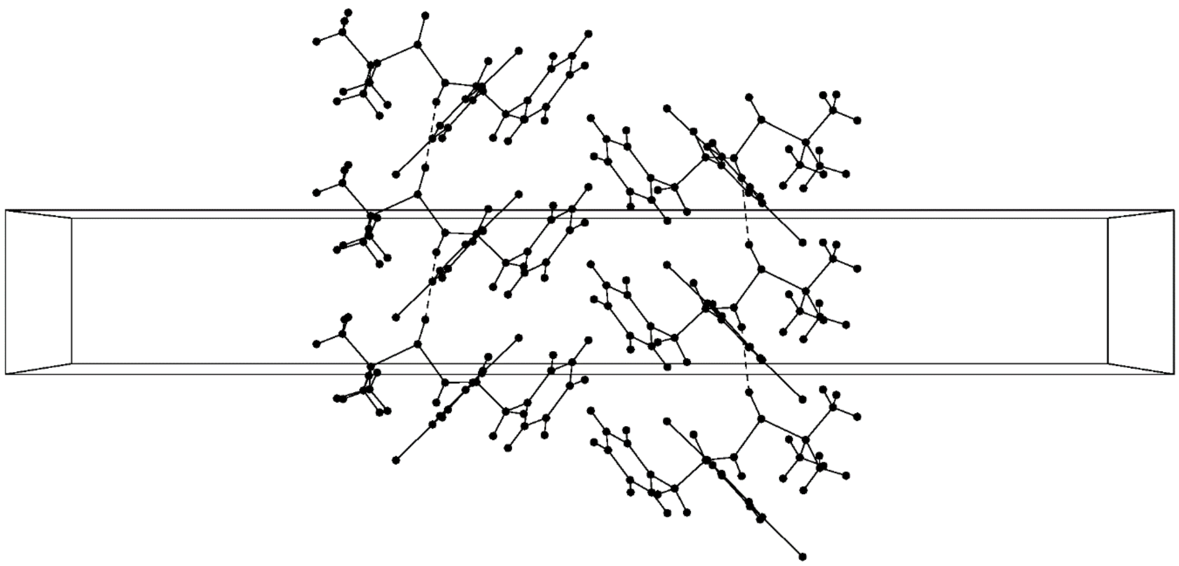

Datablock: 22100z

Bond precision: C-C = 0.0078 A
Wavelength=0.71073
Cell: a=5.3166(10) b=9.0864(13) c=37.881(7)
alpha=90 beta=90 gamma=90

Temperature: 150 K

	Calculated	Reported
Volume	1830.0(5)	1830.0(5)
Space group	P 21 21 21	P 21 21 21
Hall group	P 2ac 2ab	P 2ac 2ab
Moiety formula	C17 H20 Br2 N2 O S	C17 H20 Br2 N2 O S
Sum formula	C17 H20 Br2 N2 O S	C17 H20 Br2 N2 O S
Mr	460.21	460.23
Dx,g cm^{-3}	1.670	1.670
Z	4	4
Mu (mm^{-1})	4.549	4.549
F000	920.0	920.0
F000’	918.56	
h,k,lmax	7,12,50	7,12,50
Nref	4535[2667]	4507
Tmin,Tmax	0.585,0.695	0.468,0.563
Tmin’	0.500	

Correction method = # Reported T Limits: Tmin = 0.468 Tmax = 0.563

AbsCorr = MULTI-SCAN

Data completeness = 1.69/0.99

Theta(max) = 28.325

R(reflections) = 0.0397(4055)

wR2(reflections) = 0.0841(4507)

S = 1.100

Npar = 214

HPLC method
Instrument: Agilent 1260 Infinity HPLC

Column: Eclipse Plus C18, 3.5 µm 4.6×100 mm
Flow rate: 1.0 mL/min
Solvent A: 0.1% TFA in water
Solvent B: 0.1% TFA in acetonitrile
Solvent gradient:

Time (min)	%A	%B
0:00	95	5
2:00	0	100
12:00	0	100
13:00	95	5

Traces for representative compounds (following pages)

Figure S33: HPLC spectra of compound (**5a**).

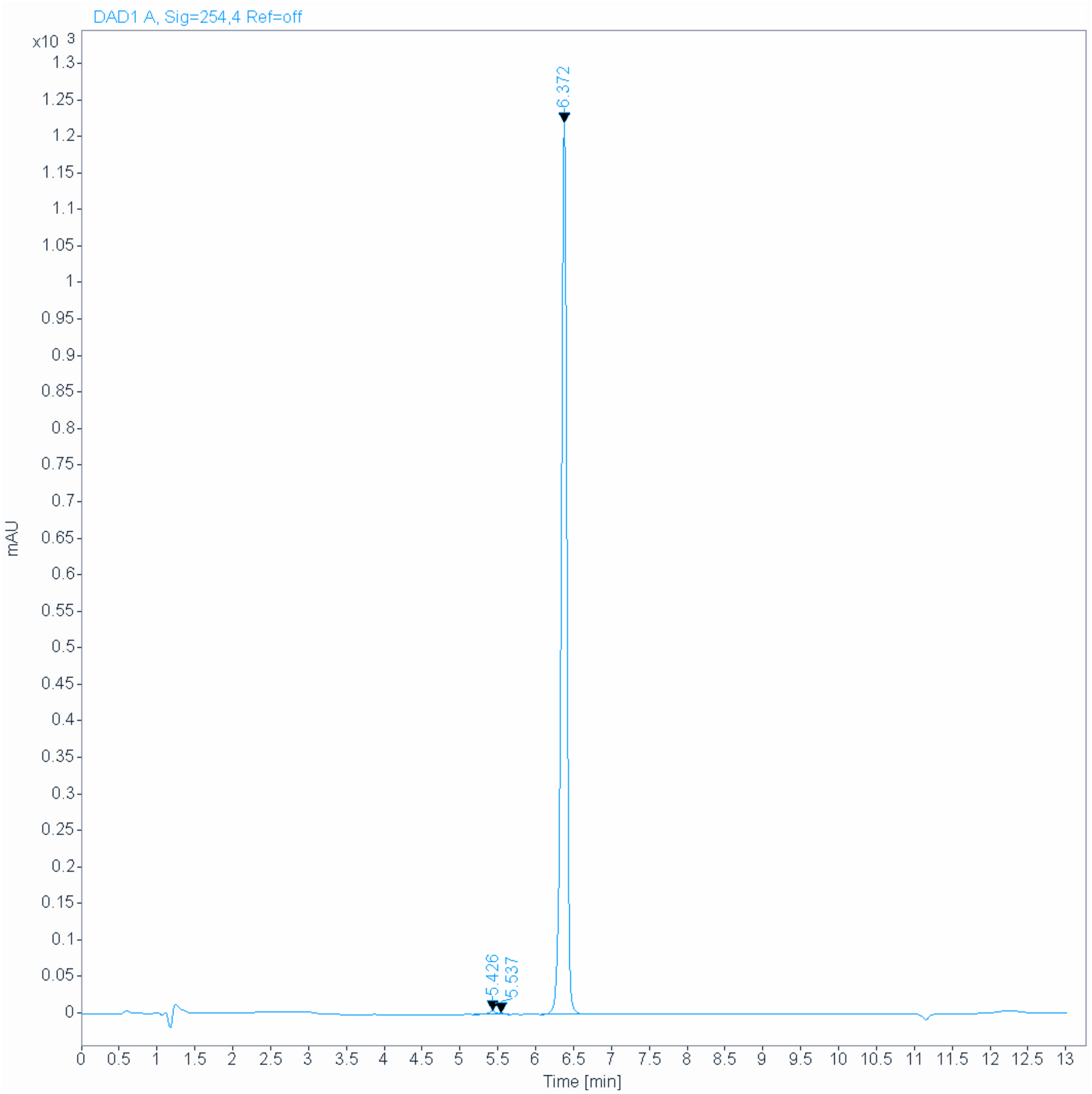

Signal: DAD1 A, Sig=254,4 Ref=off

RT [min]	Type	Width [min]	Area	Height	Area%	Name
5.426	BV	0.0842	30.5321	5.2232	0.4921	
5.537	VB	0.0619	10.0388	2.4431	0.1618	
6.372	BV R	0.0768	6164.3027	1223.1754	99.3461	
		Sum	6204.8737			

Figure S34: HPLC spectra of compound (**5b**).

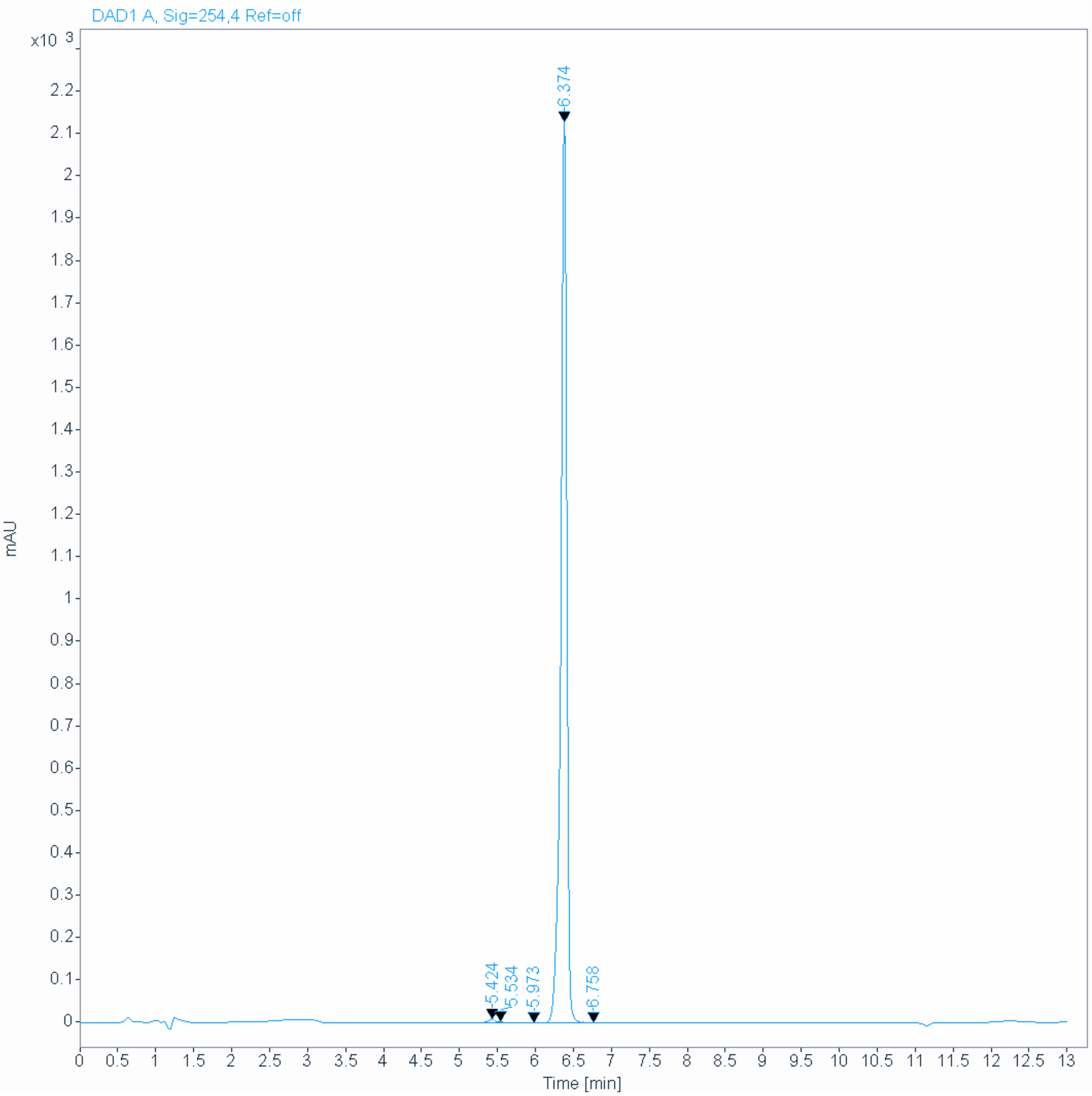

Signal: DAD1 A, Sig=254,4 Ref=off

RT [min]	Type	Width [min]	Area	Height	Area%	Name
5.424	VV R	0.0993	68.9922	9.6642	0.5852	
5.534	VB	0.0652	17.9945	4.2698	0.1526	
5.973	BB	0.0740	5.2464	1.0936	0.0445	
6.374	BV R	0.0819	11690.7578	2134.5505	99.1605	
6.758	VB E	0.0949	6.7358	1.1412	0.0571	
		Sum	11789.7267			

Figure S35: HPLC spectra of compound (**6a**).

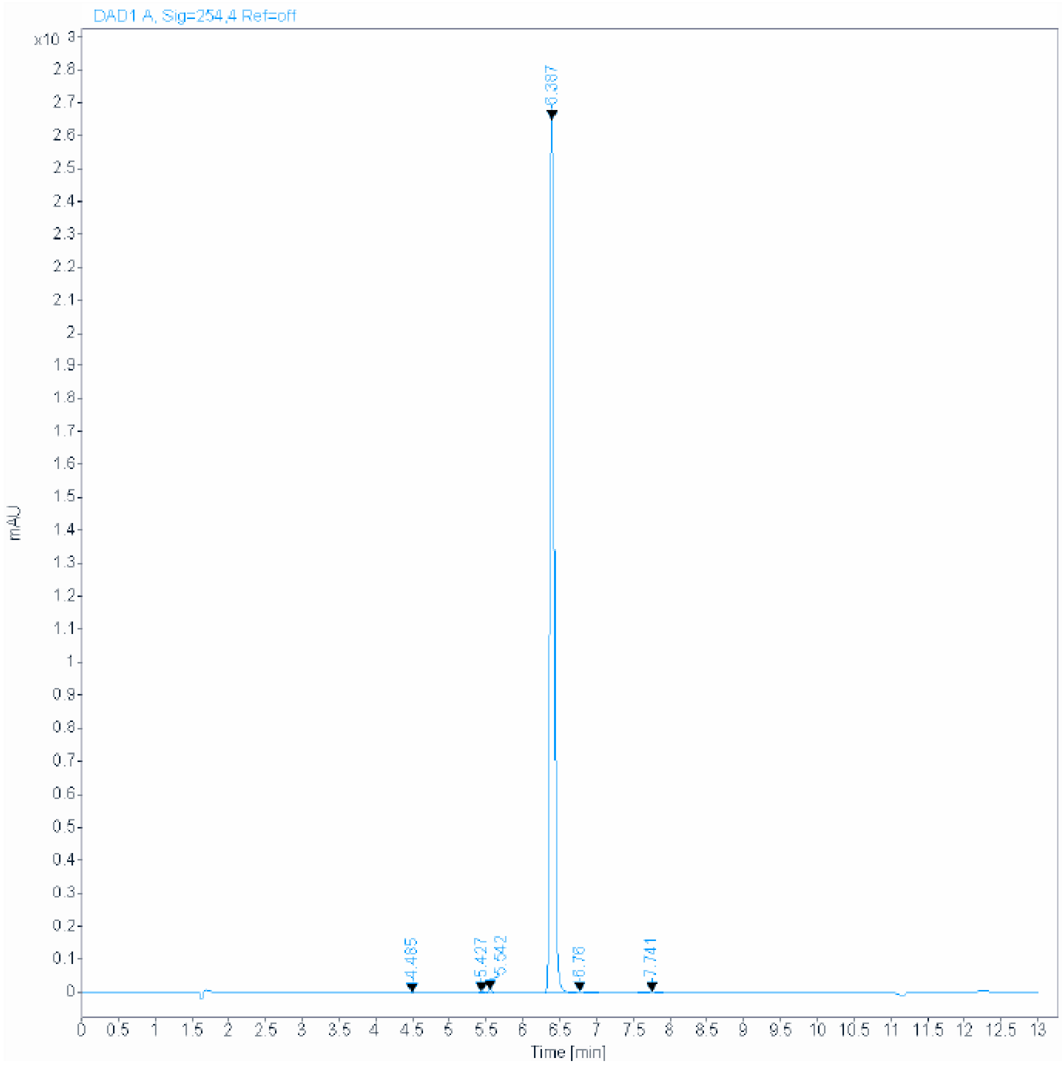

Signal: DAD1 A, Sig=254,4 Ref=off

RT [min]	Type	Width [min]	Area	Height	Area%	Name
4.485	BB	0.1498	20.8226	1.9014	0.1858	
5.427	BV	0.0654	15.0335	3.5529	0.1341	
5.542	VV R	0.0653	45.5451	10.3498	0.4063	
6.387	BV R	0.0666	11094.2607	2663.9761	98.9791	
6.760	VB E	0.0820	20.3720	3.9618	0.1818	
7.741	VV R	0.0661	12.6607	2.9472	0.1130	
		Sum	11208.6946			

Figure S36: HPLC spectra of compound (**6b**).

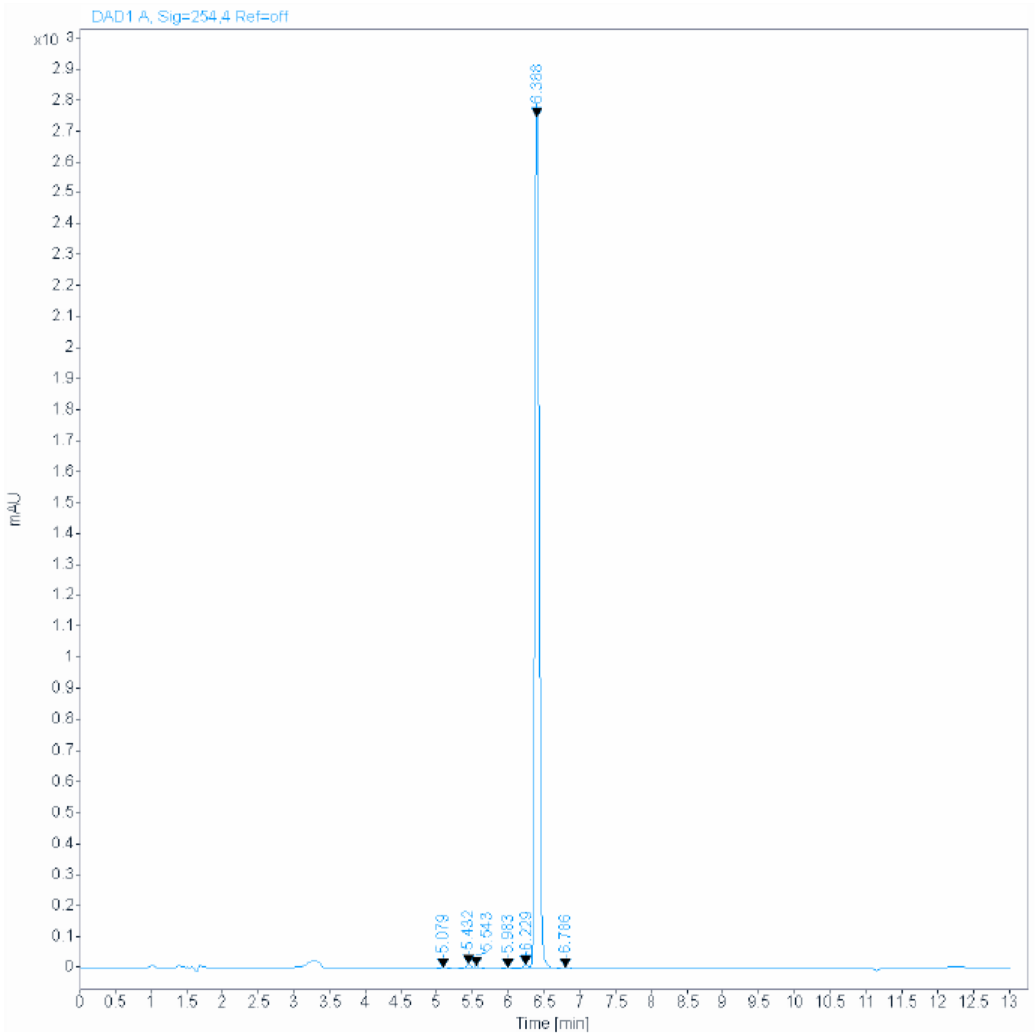

Signal: DAD1 A, Sig=254,4 Ref=off

RT [min]	Type	Width [min]	Area	Height	Area%	Name
5.079	BV	0.0832	8.6481	1.5973	0.0713	
5.432	BV	0.0662	57.4235	13.3539	0.4734	
5.543	VV	0.0620	32.8718	7.9826	0.2710	
5.983	BB	0.0689	6.2361	1.4287	0.0514	
6.229	BV E	0.0699	52.9166	11.8929	0.4362	
6.388	VV R	0.0686	11965.7813	2760.0334	98.6403	
6.786	VB E	0.0858	6.8433	1.3347	0.0564	
		Sum	12130.7206			
